# Salivary and Intestinal Transcriptomes Reveal Differential Gene Expression in Starving, Fed and *Trypanosoma cruzi*-Infected *Rhodnius neglectus*

**DOI:** 10.3389/fcimb.2021.773357

**Published:** 2021-12-17

**Authors:** Tamires Marielem Carvalho-Costa, Rafael Destro Rosa Tiveron, Maria Tays Mendes, Cecília Gomes Barbosa, Jessica Coraiola Nevoa, Guilherme Augusto Roza, Marcos Vinícius Silva, Henrique César Pereira Figueiredo, Virmondes Rodrigues, Siomar de Castro Soares, Carlo José Freire Oliveira

**Affiliations:** ^1^Laboratory of Immunology and Bioinformatics, Institute of Biological and Natural Sciences, Federal University of Triangulo Mineiro, Uberaba, Brazil; ^2^Biomedical Research Center, The University of Texas at El Paso, El Paso, TX, United States; ^3^Veterinary School, Department of Preventive Veterinary Medicine, Federal University of Minas Gerais, Belo Horizonte, Brazil

**Keywords:** *Trypanosoma cruzi*, triatomine, transcriptome, salivary glands, intestine

## Abstract

*Rhodnius neglectus* is a potential vector of *Trypanosoma cruzi* (Tc), the causative agent of Chagas disease. The salivary glands (SGs) and intestine (INT) are actively required during blood feeding. The saliva from SGs is injected into the vertebrate host, modulating immune responses and favoring feeding for INT digestion. Tc infection significantly alters the physiology of these tissues; however, studies that assess this are still scarce. This study aimed to gain a better understanding of the global transcriptional expression of genes in SGs and INT during fasting (FA), fed (FE), and fed in the presence of Tc (FE + Tc) conditions. In FA, the expression of transcripts related to homeostasis maintenance proteins during periods of stress was predominant. Therefore, the transcript levels of Tret1-like and Hsp70Ba proteins were increased. Blood appeared to be responsible for alterations found in the FE group, as most of the expressed transcripts, such as proteases and cathepsin D, were related to digestion. In FE + Tc group, there was a decreased expression of blood processing genes for insect metabolism (e.g., Antigen-5 precursor, Pr13a, and Obp), detoxification (Sult1) in INT and acid phosphatases in SG. We also found decreased transcriptional expression of lipocalins and nitrophorins in SG and two new proteins, pacifastin and diptericin, in INT. Several transcripts of unknown proteins with investigative potential were found in both tissues. Our results also show that the presence of Tc can change the expression in both tissues for a long or short period of time. While SG homeostasis seems to be re-established on day 9, changes in INT are still evident. The findings of this study may be used for future research on parasite-vector interactions and contribute to the understanding of food physiology and post-meal/infection in triatomines.

## Introduction

Triatomines, a group of paurometabolous insects with approximately 140 species, are arthropods that locate vessels and suck blood directly from them. A triatomine can cause direct damage to the host by inserting its mouthparts during feeding; however, this process also necessitates the use of bioactive molecules that aid in the transmission and propagation of pathogens, such as the flagellate *Trypanosoma cruzi* (Tc), the causative agent of Chagas disease (Mesquita et al., 2008; Mendes et al., 2016). Among the many important species of triatomines, *Rhodnius neglectus*, one of 20 species in the genus Rhodnius, is widely distributed in Brazil (Falcone et al., 2020) and plays an important role in the sylvatic maintenance of *T. cruzi* (Tc) in South America (Gurgel-Goncalves et al., 2012).

Triatomine saliva is deposited at the bite site throughout hematophagy and is required for successful feeding (Soares et al., 2006). Platelet aggregation, vasoconstriction, blood coagulation, increased vascular permeability, chemotaxis, and leukocyte immune function are all modulated by saliva (Mesquita et al., 2008; Fontaine et al., 2011; de Araujo et al., 2012; Mendes et al., 2016).

In addition to the salivary glands (SG), the triatomine intestine (INT), which stores and digests blood, is critical during pathogen transmission. In addition to absorbing nutrients and acting as a barrier against aggressor agents, Tc can transform into the infective form and can infect new vertebrate hosts during triatomine feeding and defecation (Garcia et al., 2007). Furthermore, essential molecules found in the triatomine INT can increase blood intake in a shorter period of time, protecting the vector while providing the parasite an opportunity to infect further hosts (Rossignol et al., 1985; Paim et al., 2011).

Despite the importance of SGs and INT in triatomine biology and pathogen maintenance and transmission, little is known about the expression and production of bioactive molecules from these tissues in Tc-infected or non-infected triatomines. Transcriptome studies in other hematophagous arthropods, including ticks and mosquitoes, have revealed that gene expression in these tissues actively changes and can vary depending on factors, such as developmental stage, environmental conditions, physical stimuli, physiological state, presence of infectious agents, and interactions with their hosts (Wang et al., 2009; Wolf, 2013). Understanding the triatomine transcriptome, in other words, appears to be an essential perspective to understand the transition between feeding states and vector potential, among other host-parasite interactions.

Thus, the objective of this study was to use improved bioinformatics tools to analyze the transcriptomes of SGs and INT of *R. neglectus*, whether uninfected or infected with Tc, and map the molecules related to the presence of the parasite in response to the vector’s bloodmeal.

## Materials and Methods

### Insects, Feeding, and Tissue Collection

The adults of both sexes of *R. neglectus*, reared at the Federal University of Triangulo Mineiro Triatomine Insectary (Uberaba, Brazil), were fasted for 30 days. The insects were weighed and separated into groups of 10 for the following purposes: Prolonged Fasting Group (FA): insects that did not feed (starving); Fed Group (FE): insects that fed for 2 h in an artificial feeder with human blood; and Fed and Infected Group (FE + Tc): insects that fed for 2 h in an artificial feeder with human blood infected with trypomastigotes of *T. cruzi* Colombiana strain (1 × 10^6^ parasites/mL) previously cultivated for 30 days in MK-2 cells. The success of artificial feeding was determined by weighing the triatomines before and after feeding. SGs and INTs were collected two days later from all three groups. SGs and INTs were also collected 9 days after feeding from FE + Tc to estimate the duration of potential transcriptional changes caused by the presence of Tc. SGs and INTs tissues were placed in 200 µL and 400 µL of RNAlater, respectively (Qiagen, Valencia, CA), and stored at 4°C for 2 days before being stored at -80°C until further analysis.

### Quantitative Polymerase Chain Reaction (qPCR)

The presence of Tc in the intestinal parenchyma of the FE + Tc group was confirmed by qPCR analysis. The Promega blood-tissue extraction kit (ReliaPrep™ gDNA Tissue Miniprep System) was used according to the manufacturer’s guidelines. The primers Cruzi 1 (5’-ASTCGGCTGATCGTTTTCGA-3’) and Cruzi 2 (5’-AATTCCTCCAAGCAGCGGATA-3’), labeled with 5(6)-carboxyfluorescein) and 3BHQ1™ (black hole quencher), were used to amplify a 166 bp Tc satellite DNA fragment (Piron et al., 2007). All the FE + Tc samples were positive for Tc.

### Extraction of Total RNA, Construction of Library, and Transcriptome Sequencing

RNA extraction was carried out using the RNAeasy Mini Kit (50) (Qiagen, Germantown, USA; Cat No/ID: 74104) according to the manufacturer’s instructions. The material was quantified using Qubit RNA (Thermo Fisher, Eugene, USA), and the quality was verified and validated using TapeStation (Agilent, California, USA). Library construction was carried out using the standard protocol of the TruSeq Exome kit (California, USA) (formerly TruSeq RNA Access Library Prep Kit, Illumina), albeit without the second hybridization stage. The run was performed on a paired end with 101 base pair (bp) readings.

### Bioinformatics Analysis

Sequencing quality parameters were evaluated using FastQC (v0.11.7) (https://www.bioinformatics.babraham.ac.uk/publications.html) (Wingett and Andrews, 2018). Reads were trimmed at the ends and stopped at the base with a ≥ 20 Phred score. Reads with total bp > 30% with < Phred 20 or bp > 15% with ≤ Phred 15 were removed. Barcode sequences were trimmed using Trimmomatic (v0.36) (http://www.usadellab.org/cms/?page=trimmomatic) (Bolger et al., 2014), and random sequencing errors were corrected using Rcorrector (v1.0.4) (https://gigascience.biomedcentral.com/articles/10.1186/s13742-015-0089-y) (Song and Florea, 2015), both through the Oyster River Protocol workflow (ORP v2.2.8) (https://oyster-river-protocol.readthedocs.io/en/latest/strandexamine.html) (MacManes, 2018).

*De novo* assembly was performed using a multi-k-mer (multi-assembler) approach in two steps, using Oyster and Orthofuser workflows. In the first stage, Orthofuser was used to determine the unique partial reference formed by deduplicated contigs from each sample assembly. In the second stage, the partial reference was formed by contigs assembled from the junction of the reads of all samples, normalized to a maximum of 1,000 reads of identical sequence representation. Finally, both references were merged, the contigs were again deduplicated, and those that were not mapped by Salmon (v0.13.1) (https://github.com/COMBINE-lab/salmon) were removed (Patro et al., 2017). Quality assessment of the final assembly was performed using the Oyster River Strand Exam Tool, Transrate (v1.0.3) (https://hibberdlab.com/transrate/index.html) (Smith-Unna et al., 2016), and BUSCO (v4.1.3) (https://gitlab.com/ezlab/busco/-/releases#4.1.4) (Waterhouse et al., 2018).

For transcript annotation, NCBI databases were used (non-redundant proteins – NR complete, including PIR, PDB, and RefSeq), SwissProt, UniProt, SMART, Pfam, KOG, CDD, PRK, TIGR, GO-SeqDB, and MEROPS. Protein matching with the highest bitscore (E-value < 10^-4^) for each transcript was considered using Diamond (v2.0.5) (https://github.com/bbuchfink/diamond) (Buchfink et al., 2015) and its standard composition correction was performed. The data were plotted in a spreadsheet with the script in Visual Basic Advanced, EMBLtable, created and provided collaboratively by Dr. José Marcus C. Ribeiro. Other results have also been reported.

To predict the putative protein segments of the open reading frame (ORF) translated by the transcripts, three prediction tools were used: TransDecoder (v5.5.0 – workflow Pfam-blastp) (https://www.nature.com/articles/nprot.2013.084) (Haas et al., 2013), Augustus (v3.3.3, *training set* Rhodnius) (https://github.com/Gaius-Augustus/Augustus) (Stanke et al., 2006), and ORFfinder (v0.4.3) (https://www.ncbi.nlm.nih.gov/orffinder/) (Wheeler et al., 2003). The annotation results were used to precisely determine the coding regions (CDS) and choose among the most extended protein segments. Among the segments predicted with ORFfinder of transcripts that did not have an annotation match, only the four longest transcripts were selected. From these segments, due to CDS imprecision, only the longest segment was used to determine the presence of a signal peptide (SP+ or SP-), using SignalP (v5.1) (http://www.cbs.dtu.dk/services/SignalP/abstract.php#5.0) (Almagro Armenteros et al., 2019a).

Manual global functional clustering of the transcripts was performed using the annotation results. Potential mitochondrial peptides were classified using TargetP (v2.0) (http://www.cbs.dtu.dk/services/TargetP/cite.php) (Almagro Armenteros et al., 2019b), and transmembrane peptides were predicted using TMHMM (v2.0) (https://services.healthtech.dtu.dk/service.php?TMHMM-2.0) (Moller et al., 2001). The detailed functional annotation of transcripts was performed by comparing the coding region against Kegg Orthology’s PATHWAY and BRITE databases using the online tool GhostKOALA (v2.2) (https://www.kegg.jp/ghostkoala/) (Kanehisa et al., 2016). The considered annotation had the highest GHOSTscore among matches, corresponding to the predicted ORFs of the same transcript. Finally, the detection of important sites, domains, and protein families was performed on representative ORFs of transcripts without matches or translatable into unknown or hypothetical proteins using the complete analysis of InterProScan (v5.51-85.0) (https://www.ebi.ac.uk/interpro/download/), taking into account the lowest E-value for each transcript, with a cut-off value of 10^-4^.

The number of reads aligned to the transcripts by Salmon was normalized. The differential analysis between the experimental conditions was performed using DESeq2 (v1.28.1; http://bioconductor.org/packages/release/bioc/html/DESeq2.html) (Love et al., 2014) and tximport (v1.16.1) (https://bioconductor.org/packages/release/bioc/html/tximport.html) (Soneson et al., 2015) packages, both for R. Orthologous transcripts to the biological network of *Rhodnius prolixus* (v11.0) obtained from StringDB (https://pubmed.ncbi.nlm.nih.gov/23203871/) (Franceschini et al., 2013) were identified by comparing deduplicated ORFs against protein sequences of *R. prolixus*, using Orthofinder (v.2.2.1) (https://github.com/davidemms/OrthoFinder) (Emms and Kelly, 2015). The highest bitscore defined the homologous pair sequences from the ortholog groups by directly aligning the respective transcripts against the network proteins using Diamond. Only the protein-protein interactions evidenced experimentally and with a combined score of at least 600 were considered. The final model of the network was set in Cytoscape (v3.8.2) (https://pubmed.ncbi.nlm.nih.gov/14597658/) (Shannon et al., 2003).

## Results

### *R. neglectus* INT and SG Assembly and Transcriptome Quality

**Figure 1:**
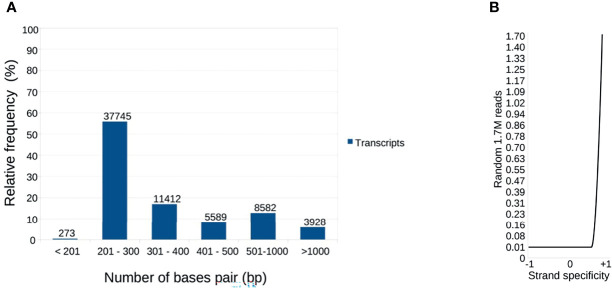
Distribution of nucleotide sequences considered for global reference. **(A)** The transcripts were distributed according to the bp. A total of 67,529 transcripts were assembled from 37,873,676 paired-reads of the salivary gland and intestine. The distribution shows that the majority (55% approximately) has between 201-300 bp. Minimum size: 133 bp; Maximum size: 28,047 bp. **(B)** Distribution of the reads used in the assemblies, according to the reading direction evaluated by the Oyster River Strand Exam Tool. Average: +0.887531.

A total of 37,873,676 paired-reads (90.23-98.92% ≥ Q30) were generated, with a Gaussian mean of 100 bp (85-102 bp). After processing, 67,529 transcripts were assembled, with an average length of 417 bp. A total of 3,928 transcripts were > 1,000 bp aligned with at least 1,000 paired-reads (**Figure 1A**).

Reading assertiveness and transcript completeness were analyzed. The assembly presented a unimodal distribution, right-skewed, with a mean of 0.887 (0.857-1.0, **Figure 1B**), as expected for paired-end assemblies (https://oyster-river-protocol.readthedocs.io/en/latest/strandexamine.html). Combining the two assembly steps with ORP allowed us to exclude uncovered contigs and assemble approximately 5,000 contigs with greater coverage than the standard workflow. The Transrate p good mapping was 0.82, p fragment mapping was 0.88, and p good contigs was 0.7, indicating good alignment quality (Smith-Unna et al., 2016). The contigs were aligned against sequences from the universal arthropod bank of BUSCO. A total of 55.6% (414) were entirely mapped by a single contig and 2.1% (16) by two or more contigs. 42.3% (315) correspond to “fragmented” or partially recovered sequences (Waterhouse et al., 2018) but did not compromise the annotations performed.

### ORFs Characterization and Taxon-Functional Annotation

**Figure 2:**
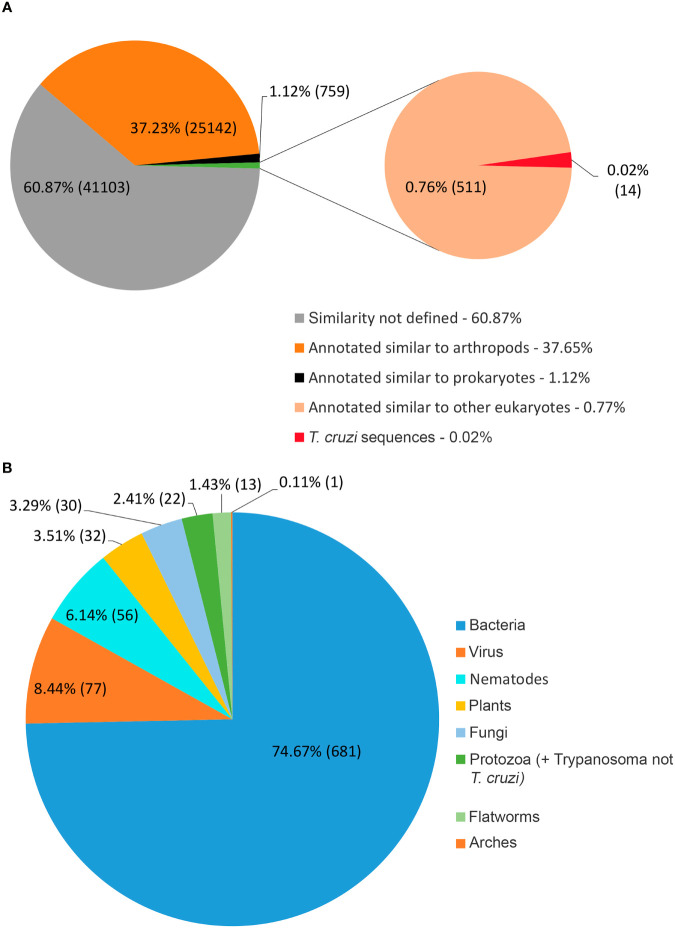
Distribution of transcripts according to annotated taxonomic similarity. **(A)** Annotated distribution of transcripts generated by pairing against sequences from public databases, highlighting arthropod annotations and those not taxonomically defined, constituted by new transcripts identified by the multi k-mer methodology. **(B)** Annotated found that resemble symbiont/residual proteins. The most significant transcripts are similar to bacterial proteins (74.7%), consistent with the salivary gland and intestine findings.

**Figure 2A** shows that 37.2% of the *R. neglectus* transcripts were similar to arthropod transcripts, 0.8% to other eukaryotes, and 60.9% showed no similarity to any taxonomic group. Among the annotated proteins from symbionts and residuals, 681 (1.0%) were similar to bacteria, 77 (0.11%) to viruses, 14 (0.02%) to Tc (FE + Tc), 22 (0.03%) to other protozoa, and 30 (0.04%) to fungi (**Figure 2B**). The prediction of ORFs is important for indicating the molecular role of the obtained transcripts (Finkel et al., 2018). From the total transcripts (**Table 1**), 904 may produce known secreted isoforms, while 16,453 may generate housekeeping proteins. The proportion of transcripts predicted to produce secreted peptides and housekeeping proteins was higher in SG than in INT. Of the transcripts producing hypothetical or unknown proteins, 349 were SP+ and 8,930 were SP-. Only 263 transcripts (0.4%) were classified as DNA transposable elements, and 4,256 transcripts that showed no similarity to any taxonomic group could still generate an SP+ sequence without annotation match.

**Table 1:** Classification of assembled and translatable transcripts into coding sequences (CDS).

Isoform category	Total transcripts			Transcripts with matched CDS group pattern		
	Total	SG	INT	Total	SG	INT
Protein predicted as secreted	904 (337)	806 (313)	794 (313)	896 (329)	801 (308)	786 (305)
	1.3% (0.5%)	1.6% (0.6%)	1.2% (0.5%)	3.2% (1.2%)	3.5% (1.3%)	2.9% (1.1%)
Housekeeping^[1]^	16453 (337)	13673 (313)	16003 (313)	16331 (329)	13590 (308)	15885 (305)
	24.4% (0.5%)	27.6% (0.6%)	24.5% (0.5%)	58.7% (1.2%)	59.2% (1.3%)	58.9% (1.1%)
Unknown/hypothetical protein predicted as secreted	349 (136)	288 (119)	339 (135)	327 (114)	273 (104)	317 (113)
	0.5% (0.2%)	0.6% (0.2%)	0.5% (0.2%)	1.2% (0.4%)	1.2% (0.5%)	1.2% (0.4%)
Unknown/hypothetical non-secreted protein	8930 (136)	7225 (119)	8693 (135)	8742 (114)	7099 (104)	8510 (113)
	13.2% (0.2%)	14.6% (0.2%)	13.3% (0.2%)	31.5% (0.4%)	30.9% (0.5%)	31.6% (0.4%)
Transposable Elements	263 (4)	202 (3)	258 (4)	241 (2)	186 (1)	236 (2)
	0.4% (0.01%)	0.4% (0.01%)	0.4% (0.01%)	0.9% (0.01%)	0.8% (0.005%)	0.9% (0.01%)
Unmatched transcript with ORF predicted as secreted	4256 (4253)	2897 (2897)	4094 (4091)	206 (203)	162 (162)	196 (193)
	6.3% (6.3%)	5.9% (5.9%)	6.3% (6.2%)	0.7% (0.7%)	0.7% (0.7%)	0.7% (0.7%)
Unmatched transcript with ORF nonsecreted	41100 (4253)	27695 (2897)	39793 (4091)	1699 (203)	1412 (162)	1627 (193)
	60.9% (6.3%)	56.0% (5.9%)	60.8% (6.2%)	6.1% (0.7%)	6.1% (0.7%)	6.0% (0.7%)
**Total transcripts**	67529	49457	65435	27796	22949	26946

TransDecoder, Augustus, and ORFfinder were used to predict the open reading frames (ORFs). Of the 67,529 total transcripts, only 27,796 (41.2%) have a CDS pattern defined by homology with the ORFfinder and/or identified/optimized by TransDecoder or Augustus (Transcripts with matched CDS group pattern). Unannotated transcripts are represented as unmatched. *(): transcripts with secretable and non-secretable signal predicted isoform segments. ^1^transcripts with known predicted non-secretable + residual/symbiont-like translatable segments. SG, salivary gland; INT, intestine.

**Figure 3:**
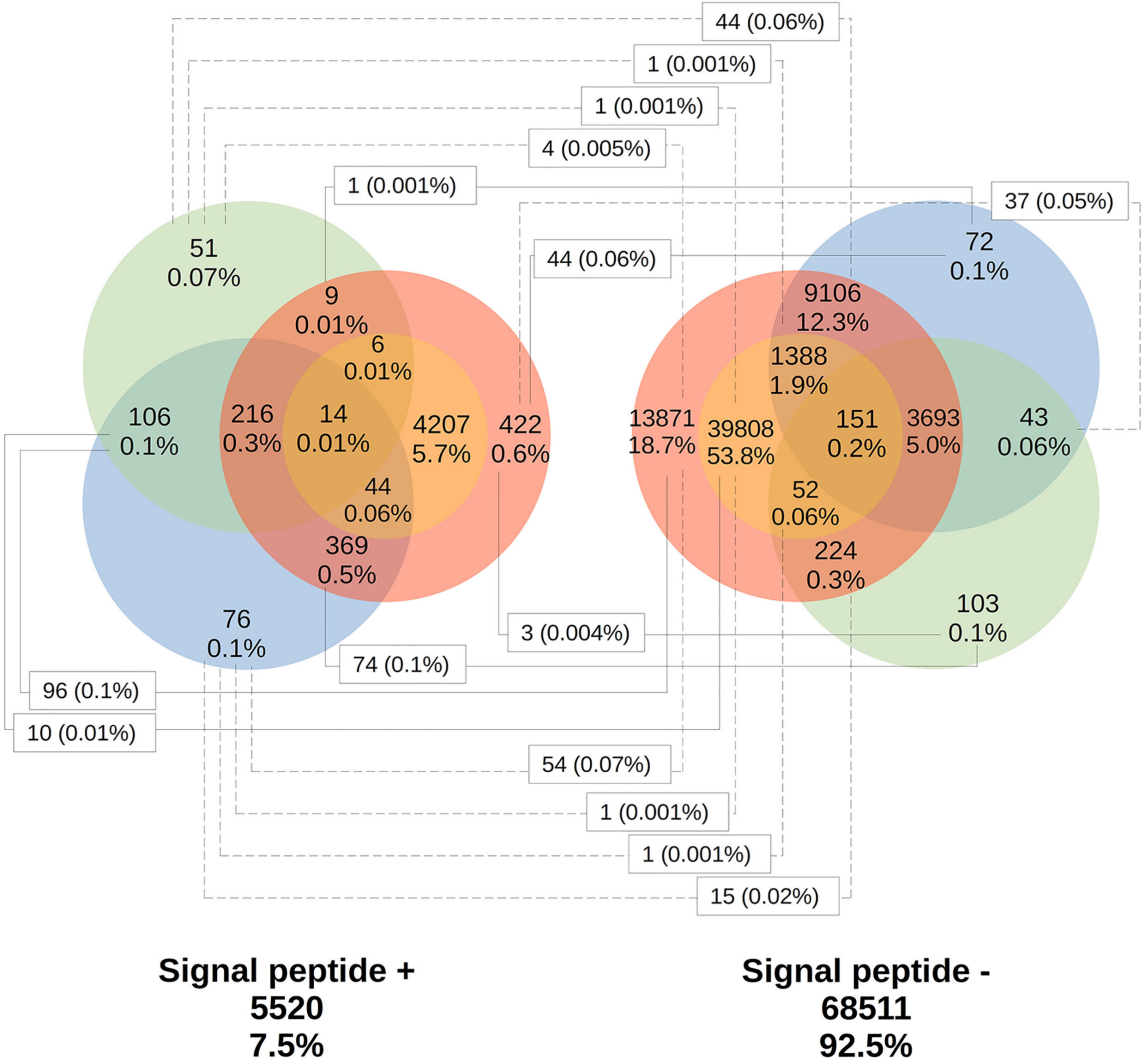
Total distribution of ORFs predicted by the existence of signal peptide. Using TransDecoder, Augustus, and ORFfinder, the total ORFs for the transcripts is 74,031. 27,910 (37.9%) with ORFfinder (Matched ORF), in red; 45,670 (61.7%) with ORFfinder (Unmatched ORF), in yellow; 15,278 (20.7%) with TransDecoder, in blue, and 4,668 (6.3%) with Augustus, in green. Only 7.5% of the ORFs have the presence of signal peptide (SP+) against 92.5% with SP-. *:_ Corresponding ORFs from the same transcript, but with different amino acid sequences and SP positivity.

**Figure 4:**
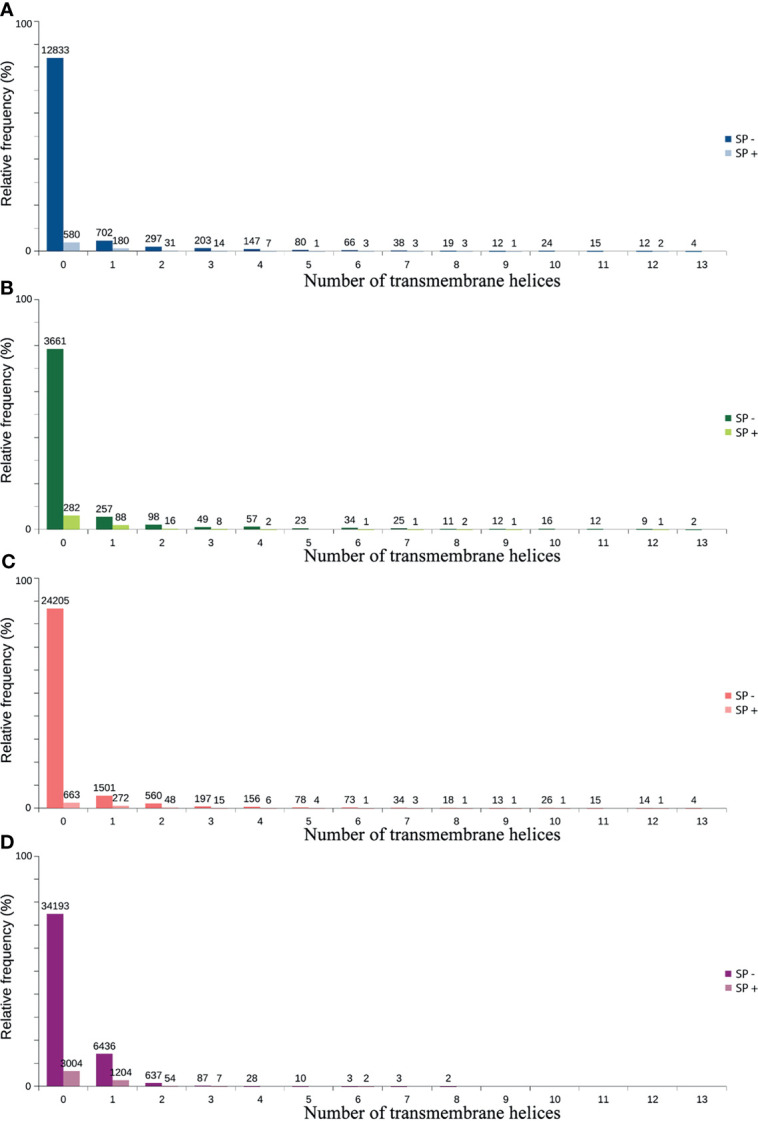
Predicted distribution of ORFs according to the number of transmembrane helices (TMHMM) and the presence of signal peptide (SP+ or SP-). **(A)** TransDecoder 15270 (20.7%). **(B)** Augustus 4668 (6.3%). **(C)** ORFfinder (Matched ORF) 27910 (37.9%). **(D)** ORFfinder (Unmatched ORF) 45670 (61.7%).

**Figure 5:**
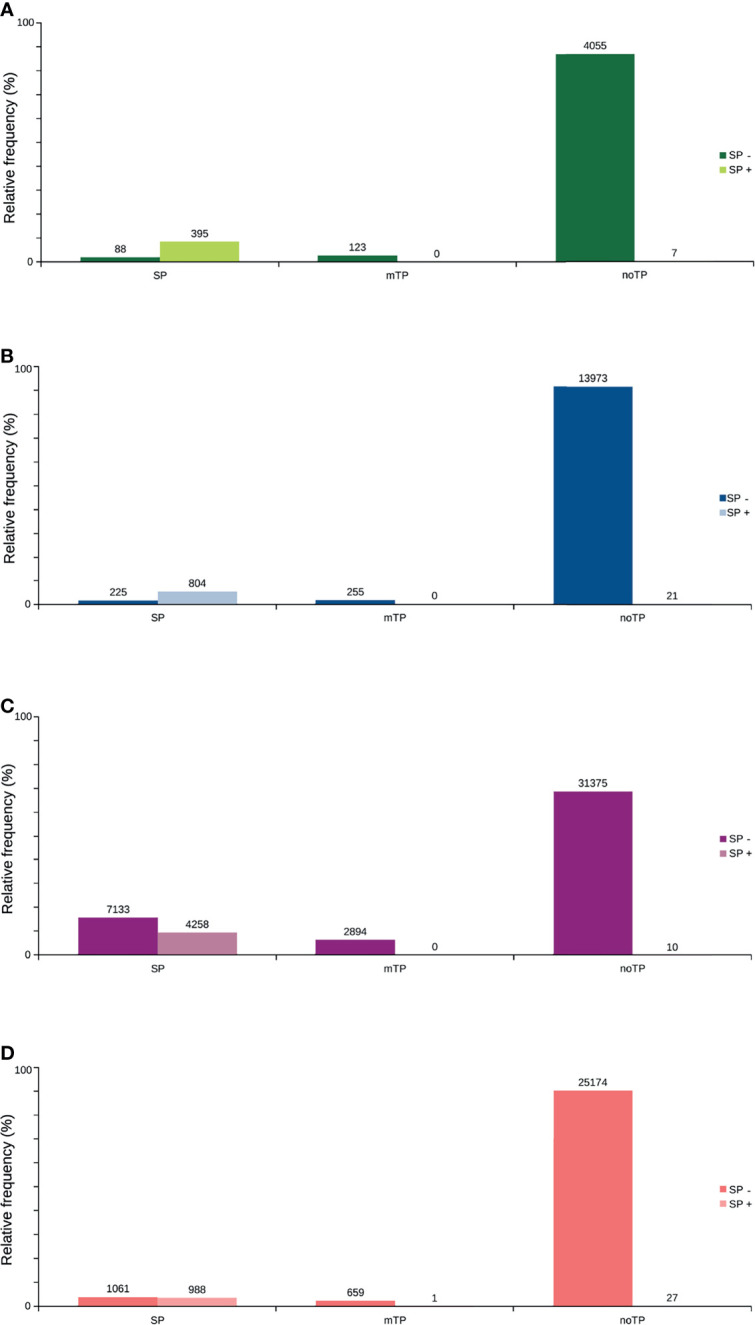
Predicted distribution of ORFs according to the N-terminus predicted by TargetP, with the presence or not of signal peptide (SP+ or SP-) predicted by SignalP. **(A)** TransDecoder 15,270 (20.7%). **(B)** Augustus 4668 (6.3%). **(C)** ORFfinder (Matched ORF) 27,910 (37.9%). **(D)** ORFfinder (Unmatched ORF) 45670 (61.7%). ORFs are capable of addressing mitochondria (mTP); with signal peptide (SP), and without signal or mitochondrial targeting peptide (noTP), according to TargetP.

Only 41.2% had homology defined CDS patterns or were identified/optimized by TransDecoder or Augustus (transcripts with matched CDS group patterns). From these, 58.7% can be classified as housekeeping, followed by 31.5% as Unknown/hypothetical non-secreted peptide generators, indicating that many CDSs are already known (**Supplementary Material Data Sheets**), even if their function is undefined. The total number of ORFs predicted by the tools was 74,031 (**Figure 3**), and only 7.5% of them were SP+. In general, ORFs without SP do not have a transmembrane helix, and their prediction can reach 98% of assertiveness (Krogh et al., 2001). This indicates that these proteins are likely to be present in the intracellular environment (**Figure 4**). Among the ORFs with only one helix, 8,896 were SP-, as expected, only 744 were SP+, and up to 1,386 may be associated with mitochondrial targeting (**Figure 5**).

**Figure 6:**
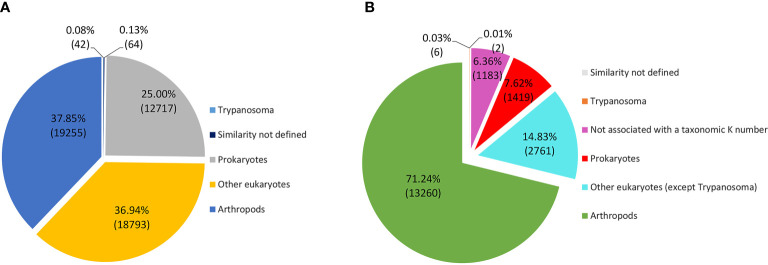
Taxonomic annotation of transcripts according to the Kegg Orthology GhostKOALA database. **(A)** Total transcripts according to taxonomy. **(B)** Functionally annotated transcripts according to taxonomy.

**Figure 7:**
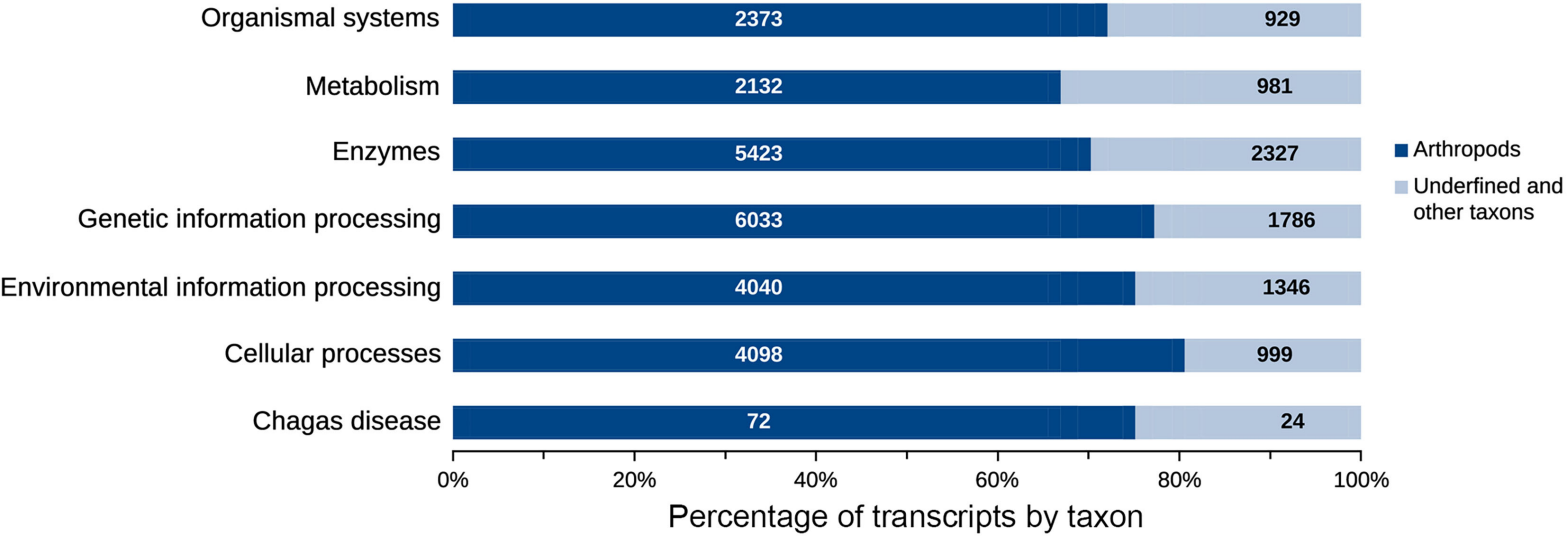
Transcripts distribution according to functional classes. The genetic information processing (7,819) and enzymes (7,750) comprise most of the transcripts present in the salivary glands and intestine of *R. neglectus*. However, known to arthropods, functional transcripts of genetic information processing and cellular processes are the most diverse of the triatomine.

The taxonomic homology of the transcripts analyzed by GhostKOALA (**Figure 6A**) indicated that most transcripts were orthologous to arthropods (37.9%), followed by other eukaryotes (36.9%), Trypanosoma (0.1%), and prokaryotes (25.0%). However, when evaluating only functionally annotated transcripts, the proportion of orthologs to arthropods increased to 71.2%, while the orthologs to other taxa were smaller. Furthermore, 6.3% of functionally annotated transcripts had undefined taxonomic orthologs (**Figure 6B**). Most transcripts, both glandular and intestinal, were related to genetic information processing (24%), followed by translation into enzymes (23%) (**Figure 7**). To avoid asserting unproven findings in arthropods, functional classes were analyzed using only arthropod orthologs.

**Figure 8:**
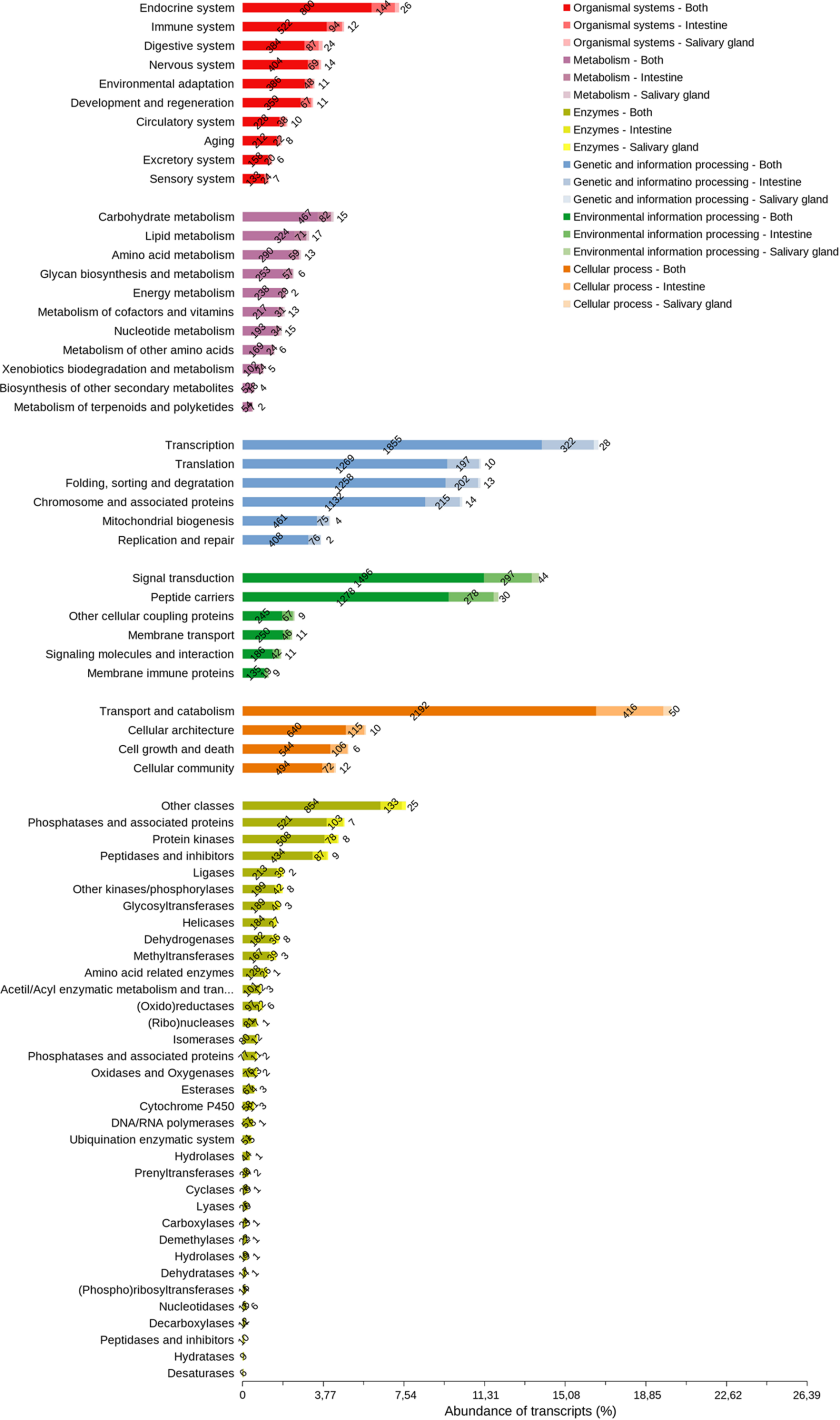
Arthropod orthologous distribution of *R. neglectus* intestine and salivary gland transcripts according to function. Other cellular coupling proteins: G protein-coupled receptors; Glycosaminoglycan binding proteins; GTP-binding proteins; Nuclear receptors. Peptide carriers: Exosomes; Transporters.

The majority of cellular process transcripts (**Figure 8**) are related to the translation of transport and catabolism components (2,658), primarily in INT, which is more diverse than in SG. The diversity of the components involved in transcription was highlighted in the processing of genetic information (2,205), whereas those involved in signal transduction (1,837) and peptide carriers (1,587) in the processing of environmental information are more diverse. Phosphatase transcripts and associated proteins (631), protein kinases (594), peptidases, and inhibitors (530) have a similar diversity of components in INT and SG. Protein transcripts with endocrine functions (970) and immunological functions (628) were the most diverse among the system components found in these tissues, and carbohydrate metabolism transcripts (565) were more diverse than those from other metabolic pathways.

### *R. neglectus* Salivary and Intestinal Secretomes

When looking at the proteins most commonly secreted in arthropods, it was possible to find a greater diversity of lipocalin transcripts (70) in *R. neglectus* SG and INT (**Supplementary Figure 2**), highlighting similarities to the so-called lipocalin precursors 4, 5, 6, and 7 as well as other procalin, triabin, and pallidipin precursors, followed by unknown transcripts of peptidases and associated inhibitors (54), and unknown secreted proteins (31). In contrast, the diversity of lipocalins and nitrophorins is higher in SG (**Figure 11**), consistent with previous findings (Santiago et al., 2018). Interestingly, there were many primary membrane-bound or intracellular protein transcripts predicted with signal peptide or with only one transmembrane helix (**Supplementary Figure 2**). The most diverse were those related to channels and (co)transport (37), predominantly in the INT (**Supplementary Figure 2**), the modification and assembly of peptides (27), and adhesion, cytoskeleton, and associated regulators (18).

### Differential Expression According to Feeding Status and *T. cruzi* Infection

In addition to their high diversity, four transcripts similar to lipocalin AI-5 precursor were among the 20 most expressed transcripts in SGs under any experimental condition, while those similar to nitrophorin 1 precursor were among the 20 most expressed transcripts in SGs after feeding (**Supplementary Table 1**). Cathepsin B was one of the top 20 most expressed genes in INT and showed increased transcription levels (**Supplementary Table 1**). Despite being present in the INT of FE + Tc, lysozyme 1 was not found among the 20 most expressed transcripts on day 2.

Furthermore, transcripts similar to hypothetical proteins were observed among the 20 most expressed transcripts, GE061_03760 (which has no known family/domains) and GE061_06167 (tryptophan aminotransferase-related protein 1 domain) were present in all groups, except FE + Tc on day 9, and GE061_01450 (consensual disorder domain) present only in FA. Two transcripts from the unknown family/domain identified in the PANTHER database as PTHR33626 were also found, but they were similar to the hypothetical protein GE061_03717 in both SG and INT, regardless of the experimental conditions.

**Figure 9:**
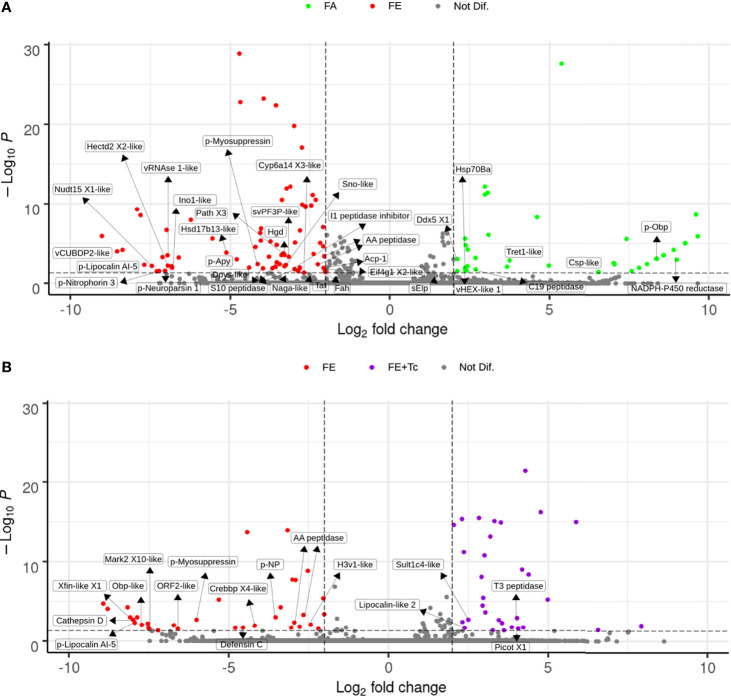
Differential expression of individual transcripts in the intestine according to blood supply and *T. cruzi* infection. **(A)** Volcano Plot of transcriptional expression in the intestine of fasted *vs.* fed *R. neglectus*. **(B)** Volcano Plot of transcriptional expression in the intestine of fed uninfected *vs.* fed infected *R. neglectus*. *Dots not assigned: unknown/hypothetical proteins. *padj < 0.05 (-Log_10_P < 50^-3^); Log_2_ fold change < -2 or > 2. *-like: transcript similar to sequence previously determined as similar to segment translated into a respective protein. *AA peptidase: subfamily AA unassigned peptidase; Acp-1: acid phosphatase-1; C19 peptidase: family C19 unassigned peptidase; Cathepsin D: cathepsin D; Crebbp X4-like: CREB-binding protein isoform X4; Csp-like: chemosensory protein; Cyp6a14 X3-like: cytochrome P450 6a14 isoform X3; Ddx5 X1: ATP-dependent RNA helicase DDX5 isoform X1; Dpys-like: dihydropyrimidinase; Eif4g1X2-like: eukaryotic translation initiation factor 4 gamma 1 isoform X2; Fah: fumarylacetoacetase; H3v1-like: histone H3v1; Hectd2 X2-like: E3 ubiquitin-protein ligase HECTD2 isoform X2; Hgd: homogentisate 1,2-dioxygenase; Hsd17b13-like: 17-beta-hydroxysteroid dehydrogenase 13; Hsp70Ba: major heat shock 70 kDa protein Ba; I1 peptidase inhibitor: family I1 unassigned peptidase inhibitor; Isyna-like: inositol-3-phosphate synthase; Lipocalin-like 2: lipocalin protein 2; Mark2 X10-like: serine/threonine-protein kinase MARK2 isoform X10; Naga-like: Alpha-N-acetylgalactosaminidase; Nudt15 X1: nucleotide triphosphate diphosphatase NUDT15 isoform X1; Obp-like: odorant-binding protein; p-Apy: 79 kDa salivary apyrase precursor; Path X3: proton-coupled amino acid transporter protein pathetic isoform X3; Picot X1: inorganic phosphate cotransporter isoform X1; p-Lipocalin AI-5: lipocalin AI-5 precursor; p-Myosuppressin: myosuppressin precursor; p-Neuroparsin 1: neuroparsin 1 precursor; p-Nitrophorin-3: Nitrophorin-3 precursor; p-NP: nonstructural protein precursor; p-Obp: odorant-binding protein precursor S10 peptidase: family S10 unassigned peptidase; sElp: secreted Esterase/lipase protein; Sno-like: senecionine N-oxygenase; Sult1c4-like: sulfotransferase 1C4; svPF3P-like: secreted venom protein family 3 protein; T3 peptidase: family T3 unassigned peptidase; Tat: tyrosine aminotransferase; Tret1-like: facilitated trehalose transporter Tret1; vCUBDP2-like: venom CUB domain protein 2; vHEX-like 1: venom hexamerin protein 1; vRNase1-like: venom ribonuclease 1; Xfin-like X1: zinc finger protein Xfin-isoform X1.

Significant changes in the expression of intestinal transcripts were detected in fed *R. neglectus* (**Figure 9A**), including a significant decrease in the gene expression of NADPH P450 reductase proteins, odorant-binding precursor protein (p-Obp), chemosensory-like protein (Csp-like), Tret1-like, DEAD-box helicase 5, hexamerin-like protein 1, Hsp70Ba, a member of the C19 peptidase family, and 24 unknown protein transcripts. On the other hand, there was a significant increase in the transcriptional levels of 20 known proteins and approximately 38 unidentified proteins, where expression levels were 2-8 times higher. These known proteins included: Cyp6a14 (similar to Isoform X3), tyrosine aminotransferase (Tat), three peptidases, myosupressin precursor (p-myosuppressin), hormone neuroparsin precursor (p-neuroparsin), an Ino1-like peptide a member of the AA peptidases, lipocalin AI-5, and nitroporin 3 precursors.

However, in the presence of Tc, the gene expression of the proteins cathepsin D, defensin C, CREB-binding protein (CREBB), histone H3v1, the same member of the AA peptidases as mentioned above, lipocalin AI-5, p-myosuppressin, and 20 unknown transcripts was significantly decreased. Simultaneously, a significant increase in the expression of 27 unknown protein genes was observed, highlighting the sulfotransferase-like peptide Sult1c4 (Sult1c4-like), lipocalin-like 2, and an unidentified T3 peptidase.

It is possible that viruses reside in the intestinal microbiota. Transcripts corresponding to viral genes were found (**Figure 2**), including those from the triatomine pathogen *Triatoma virus* (TrV) (**Supplementary Table 2**), which inevitably affects both wild and colony triatomines (Muscio et al., 1988; Muscio et al., 1997; Marti et al., 2013). Furthermore, our results suggest that the presence of *T. cruzi* in *R. neglectus* reduces the expression of transcripts from ORF 2 (Czibener et al., 2000; Ankavay et al., 2019) and nonstructural protein precursors, both from TrV (Agirre et al., 2011), possibly due to the competitiveness of these biological agents.

**Figure 10:**
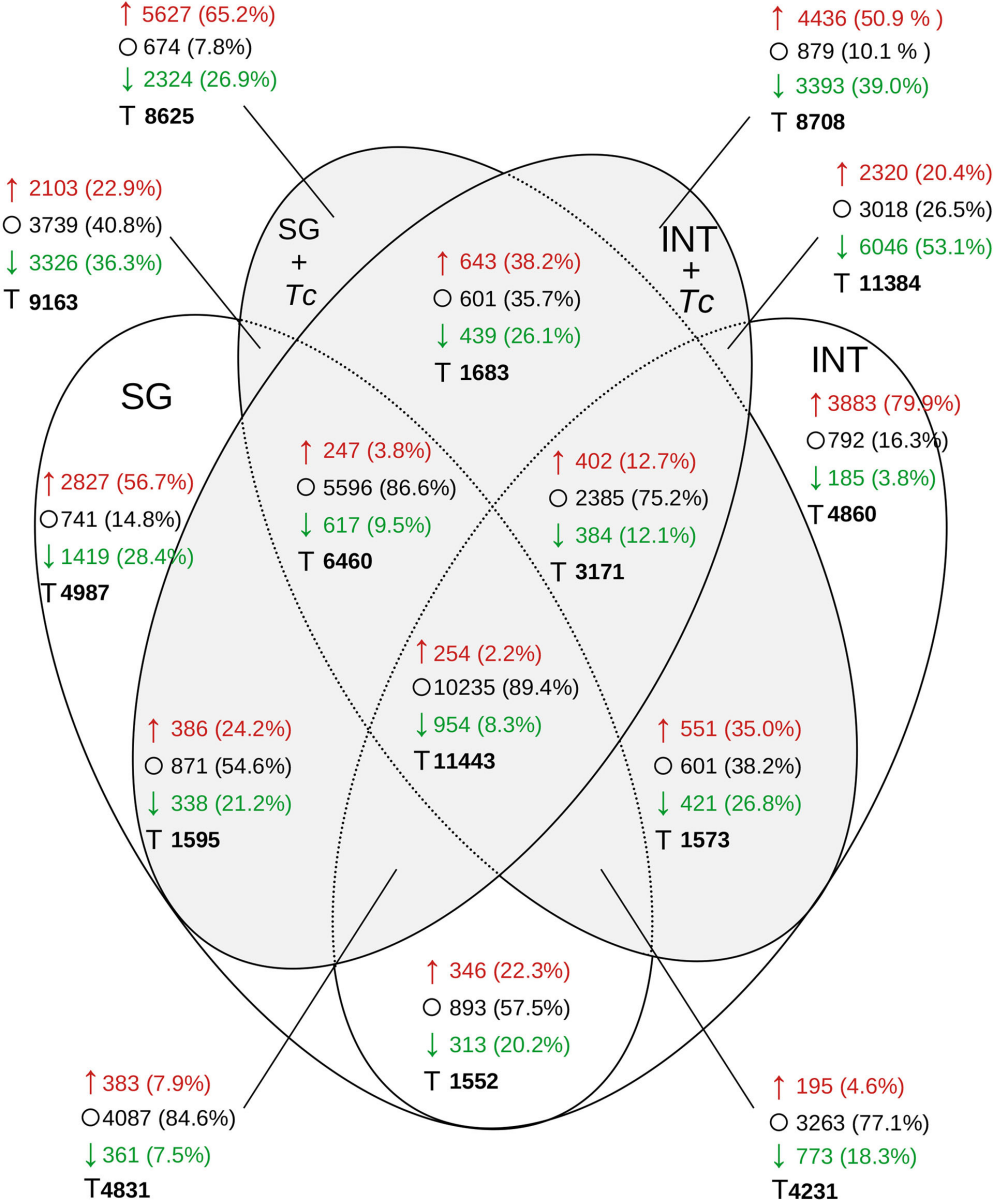
Distribution of *R. neglectus* transcripts per expression level on day 2, after feeding compared to the prolonged fasting. Transcriptional expression comparison in SGs and INT of *R. neglectus*, according to the presence of infection by *T. cruzi*, after hematophagy. *↑, 2 times upregulated; ○, Not changed; ↓, 2 times downregulated; T, Total. SG, Salivary Gland; I, Intestine; Tc, *T. cruzi*.

When the global distribution of transcripts with at least 2-fold down/upregulated expression between post-feed in FE and FE + Tc was analyzed (**Figure 10**), similar levels were observed in FE INT (4,068) or in SGs (4,246) and only 659 occurred simultaneously in both. During infection, this amount increased by 64% in both tissues simultaneously (1,082), but the transcripts differed between tissues. It is also interesting to note that the expression profile shared between any experimental condition had 11,443 transcripts but feeding and/or infection did not alter expression in 10,235 (89.4%). Even with less diversity, the majority of SG transcripts showed no change in expression after feeding. For tissue-specific transcriptional changes, regardless of the infection status, the expression of 8,366 transcripts in the INT and 5,429 transcripts in the SGs changed. In the presence of Tc, there were 7,829 and 7,951 transcripts in the INT and SG, respectively. Despite the fact that the infected INT had 14.2% more transcripts showing 2-fold increase compared to that in fasting (4,436), the number of transcripts showing less than 2-fold increase improved from 3.8% to 39% (3,393).

**Figure 11:**
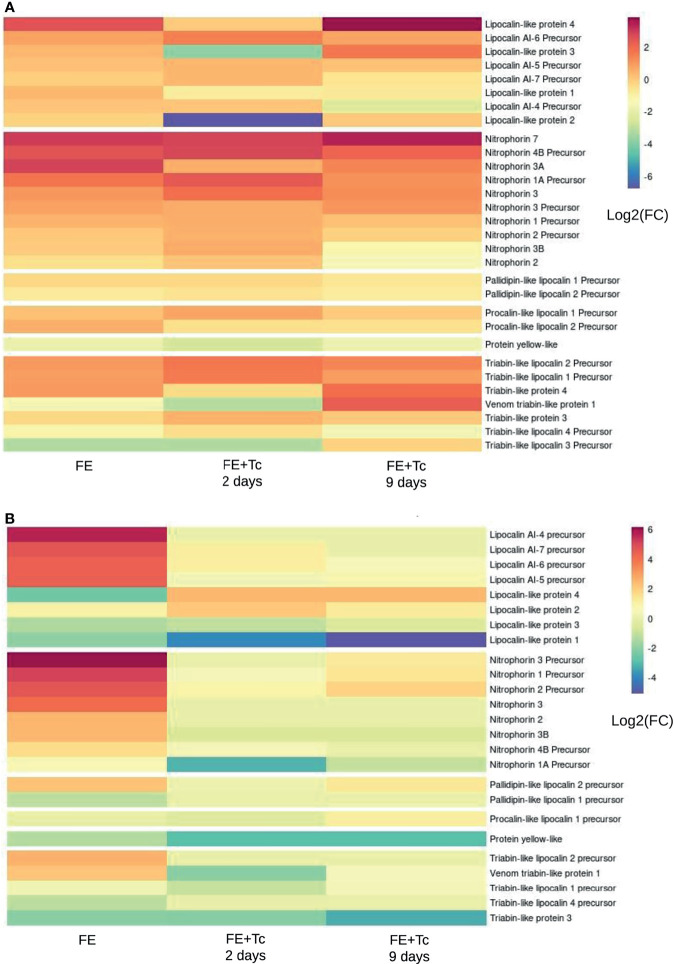
Transcripts translatable into lipocalins, nitrophorins, and derivatives. **(A)** Differential expression of individual transcripts according to the blood supply and infection by *T. cruzi* in the salivary gland of the insect in relation to prolonged fasting. **(B)** Differential expression of individual transcripts according to blood feeding and *T. cruzi* infection in the insect’s intestine in relation to prolonged fasting. *FC, fold change.

In general, the protein profile expressed in SGs FE + Tc differs from that in FE. The Kazal domain peptide Pr13a is one of the most expressed transcripts in the FA SGs (**Supplementary Figure 4A**), whose mean expression was similar in FE but decreased in FE + Tc on day 2 and markedly increased on day 9. Among the main triatomine kratagonists (**Figure 11**), there was a significant increase in the gene expression of the *R. prolixus* lipocalin 4-like protein in *R. neglectus* SG. However, the expression remained the same in FE + Tc at day 2, reaching a higher value only on day 9. The expression of the *Pristhesancus plagipennis* lipocalin AI-4 precursor-like protein remained unchanged at day 2. However, with Tc infection, the mean transcriptional expression of proteins similar to lipocalins 2 and 3 (*R. prolixus*) showed 64 and 16-fold decrease, respectively, whereas proteins similar to AI-6 precursor (*P. plagipennis*) showed 4-fold increase on day 2 compared to those in prolonged fasting, with values only recovering to this level on day 9.

There was a significant increase in the expression of proteins similar to those of nitrophorins 7 and 3A and similar nitrophorins 1A and 4 B precursors (all from *R. prolixus)*, in post-meal SG. The expression pattern of nitrophorin 7 remained unchanged until day 9. However, there was an increase in the nitrophorin 1A and 4 B transcript levels, which decreased on day 9. During infection, the mean transcription of nitrophorin 3A returned to FA levels. Segments similar to triabin 4 (from *P. plagipennis*) and triabin 1 and 2 precursors (from *R. prolixus*) had higher gene expression within 2 days after feeding, whereas triabin 3 (*P. plagipennis*) and triabin 3 and 4 precursors (*R. prolixus*) had lower expression. With the exception of the triabin 3 precursor transcripts, which were up to 16 times smaller, the altered mean levels were discrete.

In the SGs of FE + Tc, the expression of triabin 1 and 2 precursors was even higher at day 2, matching the uninfected SGs only on day 9. In contrast, triabin 4 maintained an average starvation level 2 days after feeding, increasing only on day 9. A transcript similar to triabin 1 (from the venom gland of *P. plagipennis*), whose mean increase and decrease in levels are more significant, exhibited similar behavior. On the other hand, regardless of parasite presence, the mean gene expression of triabin 3 and 4 precursors tended to equal the expression of FA on day 9. Regardless of the condition, the average expression profile of pallidipin and procalin transcripts found in SGs was the only one that did not change significantly. The mean expression of yellow protein transcripts remained constant under all conditions, albeit lower than that during fasting.

Overall, most of the most transcriptionally expressed *R. neglectus* housekeeping proteins on day 9 of infection had mean levels similar to those of prolonged fasting (**Supplementary Figure 5**). The reduction in expression was greater in FE + Tc than in FE for housekeeping genes, which exhibited lower expression after 2 days, and continued to be reduced on day 9. Among the most expressed transcripts and translatable into unknown/hypothetical non-secretable segments (**Supplementary Figure 7**), the most expressed transcripts after feeding had a domain similar to phospholipase A2. It is also interesting to highlight the sharp decrease in the transcriptional levels of three proteins in FE + Tc, one without a known domain (LOC111056117), which also decreased in FE, and two with domains similar to the binding of chitin (LOC106674348) and sodium-coupled monocarboxylate transporter (LOC106677625), whose levels increased on day 9. Two of the 50 least expressed proteins, with domains similar to lipopolysaccharide (LPS)-induced tumor necrosis factor-alpha factor (CAA9997534.1) and a disordered protein region, DPR (BAN20224.1), also showed a decreased expression only in FE + Tc on day 2 but increased expression on day 9. This expression profile was also observed in 11 unmatched transcripts among the most expressed non-secreted peptides (**Supplementary Figure 9**), with only four having identifiable domains and being similar to, in the decreasing order of expression, histone linker H1/H5, DPR, tropomyosin, and DPR.

A transcript with a coil motif, similar to BAN20609.1, was among the unknown/hypothetical secreted proteins that showed the highest transcriptional expression after feeding, and it was more highly expressed in FE and FE + Tc. Six proteins (including ATU83012.1, ATU83020.1, LOC106661678, and GE061_22476), two of which contained identifiable domains to the crystallin family (ATU82838.1) and MBF2 transcription activator (ABR27885.1), showed decreased expression on day 2 after infection and increased expression on day 9. On the other hand, the majority of the 50 most expressed and secreted unmatched transcripts had higher expression in FE + Tc than in FE. With the exception of the first two, the 50 least expressed and secreted unmatched transcripts (**Supplementary Figure 8**) had an inverse profile, and the transcript with the DPR domain exhibited the same profile as the unknown/hypothetical secreted proteins described earlier.

It is worth noting that lysozymes in INT showed higher gene expression in FE and FE + Tc on the 9th day, but with a mean decrease in FE + Tc on day 2 (**Supplementary Figure 10A**). The transcriptional expression of juvenile hormone (JH) differed slightly between FA and FE. On day 9, however, there was a sudden decrease in the expression of JH, matching the fasting level. Acid phosphatase gene expression increased in FE but decreased in FE + Tc, increasing only on day 9, whereas vitellogenin levels showed marked increase in SGs in FE + Tc on day 9 (**Supplementary Figure 4A**). Cathepsins, defensins, and snake venom cystatin-like cystatin had low transcription levels in FE and even lower in FE + Tc on day 2, with cathepsins slightly higher on day 9 (**Supplementary Figure 4B**).

In INT, p-myosuppressin and neuroparsin 1 were among the most expressed transcripts after feeding compared to starvation, with the same expression profile as previously mentioned (**Figure 9**). Some transcripts among the 50 highest expressed in the INT but not among the 50 highest expressed in SG had increased expression in FE and decreased expression in FE + Tc. These include: a Venom cub domain protein 2-like transcript (from *P. Plagipennis* venom - Walker, 2017) related to developmental processes (Bork and Beckmann, 1993), an antigen-5-like transcript described in SGs from *T. brasiliensis* (Santos et al., 2007) and *R. prolixus* (Ribeiro et al., 2004), and transcripts similar to salivary platelet aggregation inhibitors, as was identified for salivary platelet aggregation inhibitor 1 (T1HDI2) in the INT of *R. prolixus* post-feeding (Ouali et al., 2021); other transcripts, such as those similar to the venom glycin-rich peptide Pp23a, pacifastin, diptericin, and acetyl CoA synthetase, showed lower expression in FE than in FE + Tc at day 2. Among the 50 least expressed transcripts in the INT were transcripts similar to venom polipophorin-like protein 1 and venom peptide Pp26a, both of which showed a more significant decrease in expression on day 9. Transcripts similar to p-Obps showed a greater reduction only on day 2 while Niemann-Pick C1 (NPC1), showed a considerable reduction after day 2 of infection.

As in SG, nitrophorins and triabins displayed higher transcriptional expression in the INT of uninfected *R. neglectus* than in that of the infected group (**Figure 11B**). Lipocalin 4, which had higher transcriptional levels in the SG of FE + Tc than in that of FE on day 2, was higher in the INT of FE than in that of FE + Tc on day 2. Pallidipin 1 was expressed more subtly in the INT, but its expression did not differ between fasting and infection and did not differ across any condition in SGs. Under the same conditions, the expression of lipocalin AI-6 precursor was higher in the SGs of FE + Tc than in those of FE and lower in the INT under the same conditions. Only in FE the lipocalins AI-7 and 5 precursors were transcriptionally overexpressed. Lipocalin 3 had lower transcriptional expression in FE and FE + Tc, even on day 9, whereas lipocalin 2 exhibited lower expression in the SG of FE + Tc on day 2, but expressed a higher level of transcription in INT under the same conditions. Under all experimental conditions, both the most and the least expressed housekeeping protein transcripts had very similar expression profiles in INT and SGs, though they were not the same between the two tissues (**Supplementary Figure 11**). In FE +Tc, the overall mean transcriptional expression of secreted or non-secreted *R. neglectus* transcripts tended to match that of the prolonged fasting state on day 9.

### Transcriptional Profile of SG and INT Biological Networks of *R. neglectus*

**Figure 12:**
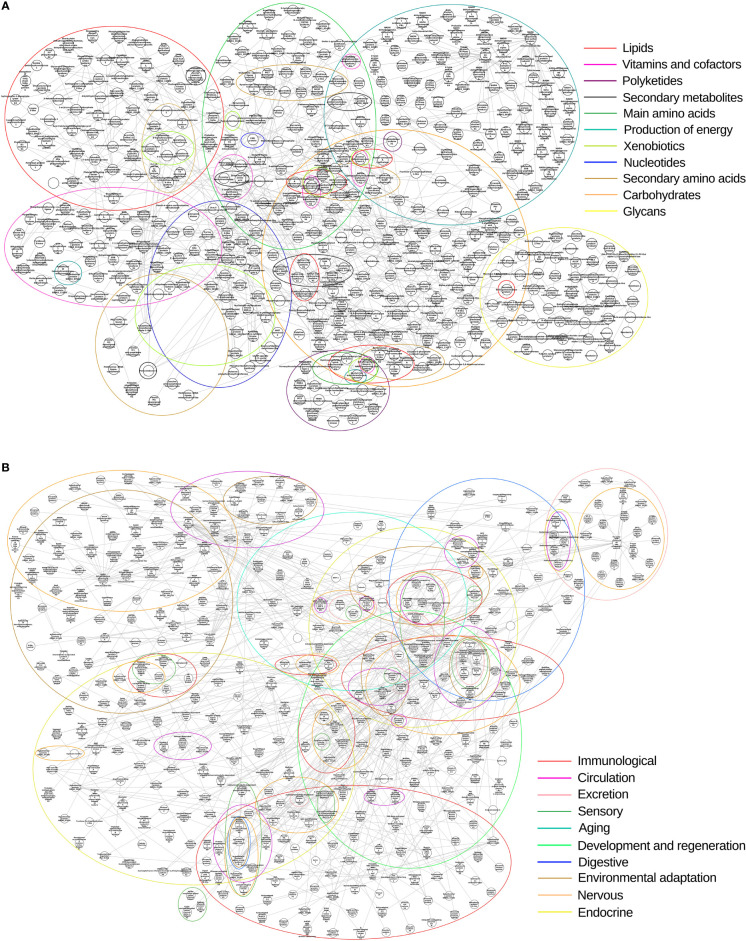
Biological network of *R. neglectus*. **(A)** Biological network of biosynthesis and intratissue metabolism pathways. **(B)** Biological network of systemic component pathways. Identified homologous component clusters with degree > 0. Unmatched transcripts are represented in unnamed nodes.

We clustered the subnetworks of metabolic pathways and systemic components based on transcript annotation to assess the interactions and expression of nodes (**Figure 12**). Regardless of the experimental conditions, the majority of the protein transcripts from metabolic/biosynthesis and systemic pathways were with higher mean expression after feeding, with the exception of secondary metabolites, carbohydrate, immune, developmental and regeneration, and endocrine pathways on day 2 of infection in SGs (**Supplementary Figure 16**). However, many nodes had reduced expression in the SGs: all metabolic pathways, the endocrine pathway on day 2 of infection, and the immunological pathway on day 9. However, the metabolic pathways of INT showed reduced transcriptional expression only on day 9 (**Supplementary Figure 17**). Most components of the immunological pathway also showed decreased expression in the SGs of FE and in the SGs and INT of FE + Tc on day 9. This was also true of the development and regeneration pathway in SGs. In the endocrine pathway, this pattern was observed during infection and on day 9 in the INT. This was also observed in the environmental adaptation pathway of both tissues, but only on day 9.

The following proteins are considered critical because they have a higher node degree and therefore deserve greater attention in future research: the hypothetical proteins similar to GE061_15329, which are related to the nervous, aging, and immune systems of *R. neglectus*; GE06113134, which is related to the sensory, nervous, immune, and endocrine system; GE06122676, which is involved in the nervous and sensory system; and WR2507110 isoform B, which is involved in all systems, except for aging, excretion, and development and regeneration.

## Discussion

### Multi-K-Mer Approach for Discovery of New Sequences in Arthropod Transcriptomes

The ability to identify more sequences (known or new) using the multi-k-mer approach enabled the identification of a wide range of previously unknown *R. neglectus* transcripts (**Figure 2** and **Table 1**) because the multi-k-mer assembly allows for the identification of less frequent and more complete contigs by maximizing the presence of sample reads. Some regions are assembled with lower K-mer values, as is the case for genes with low expression levels (Gruenheit et al., 2012), while other regions are favored with higher K-mer values, since the distribution of reads is different from other positions within the same analyzed sequence (Miller et al., 2010; Feldmeyer et al., 2011; Durai and Schulz, 2016). Thus, the multi-k-mer method enables several regions to be obtained for a final reference (Miller et al., 2010; Surget-Groba and Montoya-Burgos, 2010; MacManes, 2018).

When these assembled sequences were compared to the SG transcripts of *R. neglectus* assembled by Santiago et al. (2016) using the traditional method (**Supplementary Figure 1**), the superior yield obtained by the multi-k-mer method is evident. 97.2% (1,739) of the peptide sequences amenable to construction/availability with the work of these authors are also present in our work. However, we were able to assemble/predict thousands of other diverse contigs and isoforms of transcripts and ORFs, even considering only the sample reads from the SGs (**Figures 1**–**3**).

### Populations of Transcripts Translatable Into Secretable and Non-Secretable Peptides in *R. neglectus* SGs and INT

The ratio of translatable transcripts in secreted and housekeeping peptides in *R. neglectus* SGs and INT (**Table 1**) was similar to that found in the literature for other triatomines. *T. brasiliensis* (Santos et al., 2007) had the most secreted proteins in SGs, followed by: *R. prolixus* (Ribeiro et al., 2004), *Panstrongylus lignarius* (Nevoa et al., 2018), and *Triatoma dimidiata* (Kato et al., 2010); with housekeeping following in second in the number of transcripts found in SGs of *T. dimidiata* and *R. prolixus* (Ribeiro et al., 2004; Kato et al., 2010). In *R. neglectus* SGs, a higher number of transcripts of proteins classified as housekeeping were also found than in the other classes analyzed (Santiago et al., 2016). Other hematophagous arthropods share this profile. Among the glandular transcripts of the fed tick *Ornithodoros rostratus*, those representing secreted proteins were found to be more diverse in the SG than in the gut, while housekeeping proteins were more diverse in the gut (Araujo et al., 2019). This was also observed in the evaluation of the transcriptome and proteome analyses of *Ixodes ricinus* SG and gut following a bloodmeal (Schwarz et al., 2014a).

Transmembrane proteins are extremely important for the cell, as they can initiate various signaling processes and transport various substances across the membrane (Moller et al., 2001). Interesting some SP+ ORFs were detected containing a transmembrane helix (**Figure 4**). This may be due to the differences in accuracy between the software used and the possibility that certain transmembrane proteins present signal peptide but are not secreted in the vast majority of cases. This occurs with for example, some receptors, and the presence of the SP is believed to assist the hydrophilic N-terminus in crossing the membrane (Liu et al., 2020). However, some of these SP+ proteins also appear to represent certain precursor molecules prior to secretion, as can be seen among the molecules that are supposed to make up the glandular or intestinal secretomes (**Supplementary Figure 2**). As such, for the SP- (or even SP+) ORFs and with two or more transmembrane helices, it is suggested that they may indeed be crowded into the cell membrane (Ferguson and Williams, 1988; Nielsen, 2017; Liu et al., 2020). Finally, SP+ and the absence of a transmembrane helix may represent potentially secreted proteins (4,529) (Nielsen, 2017).

The fragmentation of a certain number of nucleotide sequences observed in the results of the BUSCO analysis indicated that the SP may not have been identified in them. Furthermore, it is possible that some transcripts could not be selected, as shown in our results. Thus, the expected prediction of secreted molecules in each group or class may be higher in the saliva and intestinal lumen of *R. neglectus* (**Table 1** and **Supplementary Figure 2**).

Therefore, the majority of the predicted ORFs are disparately noTP, possibly because proteins related to essential intracellular processes may be responsible for this group showing high affluence. However, it is possible to observe many ORFs defined as SP by SignalP, which were classified, on the other hand, as having SP by TargetP (**Figure 5**). As it has a recent revision, implements deep neural networks in its predictive analyses, and has better sensitivity and specificity rates, we prefer to define the SignalP classification as the most assertive for further analyses (Almagro Armenteros et al., 2019a).

### Compensatory Mechanisms in Prolonged Fasting to Blood Availability: Base Expression in *R. neglectus* SGs and INT

A detailed analysis of the behavior of transcripts against the bloodmeal (FA × FE) allowed us to observe what changes occur when the insect feeds. In starving, the findings are particularly important and expected because they are directly related to the feeding process and survival during periods of food scarcity. Among the mechanisms important in maintaining physiology during this period, we found that Tret1-like and hexamerins were most expressed during prolonged fasting (**Figure 9A**). Tret1-like is a transporter protein that mediates the transfer of trehalose from the fat body to other tissues, controlling the levels of this sugar in the hemolymph. Elevated levels of enzymes that catabolize trehalose have already been detected during the fasting period in *R. prolixus* ovaries (Leyria et al., 2020a), which shows the importance of these molecules for the organ balance of triatomines in this phase.

Hexamerins are proteins essentially related to the storage of amino acids during nutrient-starved stages and during the development of the insect until it reaches the adult stage. The amino acids stored by this protein are made available in the hemolymph according to the demand of the insect (Beintema et al., 1994; Hathaway et al., 2009). These proteins are also believed to have other functions as they are found in various tissues of the *Locusta migratoria* grasshopper (Li et al., 2017), such as the ability to bind and transport hormones, such as the JH (Spit et al., 2016; Li et al., 2017), related to growth and reproduction, and may participate in controlling the production of digestive enzymes (Noriega et al., 1997; Bian et al., 2008). The increase in transcript expression of this protein may be explained by the provision of alternative energy during starvation, in which there is nutrient scarcity and decreased JH expression (**Supplementary Figures 4A, 10A**).

Driven by hunger, *R. neglectus* needs to search for a host to feed, which may explain the increased expression of the transcript for Csp-like (**Figure 9A**), a protein related to the ability to perceive chemical substances that are important for the insect, in feeding, reproduction, and survival, and has already been identified in several triatomine tissues (Ribeiro et al., 2014; Marchant et al., 2016; Martinez-Barnetche et al., 2018).

In order to prepare for food receptivity while still fasting, genes involved in diet processing were found to be higher during this period (**Figure 9A**), highlighting that NADPH P450 reductase is present during metabolism and food detoxification (Scott and Wen, 2001; Feyereisen, 2006; Mamidala et al., 2011); p-Obp is required for the maintenance of intestinal physiology and for nutrient transport (Ribeiro et al., 2014), but its true importance in hematophagy still remains unknown (Ribeiro et al., 2012); an unknown C19 peptidase, a family that deconjugates ubiquitin (Rawlings and Barrett, 1994; Eletr and Wilkinson, 2014) of proteins, which should be protected from higher blood temperatures or should not yet be degraded as they will have some role in digestion (Feder and Hofmann, 1999; Ouali et al., 2020; Ouali et al., 2021); and Hsp70Ba. Heat shock proteins are highly conserved among living organisms, and HSP70 plays an essential role in protein folding and transport, and are present in abundance under conditions of cellular stress (Karlin and Brocchieri, 1998). The finding of Hsp70Ba overexpression in starving insects is consistent with the stressful situation that fasting can bring to the beetle, and a member of its family has already been found to be transcriptionally highly expressed in fasting *T. infestans* (Kollien and Billingsley, 2002) and *R. prolixus* (Leyria et al., 2020b).

Although the overall gene expression was slightly lower during prolonged fasting (**Figure 10**), a period in which both metabolic pathways and systemic component pathways in each tissue had lower expression (**Supplementary Figures 16A1, B1, 17A1, B1**), the existence of a certain level of transcription and translation of several genes, secreted or not, is still required. Thus, we can cite the increased expression of the enzyme Ddx5 X1 (DEAD box helicase 5; **Figure 9A**), also known as p28, which has a function related to RNA processing (Legrand et al., 2019), such as transcription, splicing, mRNA biogenesis, mRNA export, ribosomal biogenesis, and others (Cheng et al., 2018; Taschuk and Cherry, 2020).

### Feeding Promotes Expression of Important Molecules to Degrade Food and Compete With Microbiota for Nutrients to Prevent Early Interruption of Hematophagy by Vertebrate Host

Triatomines are insects that feed once on the host and consume a large amount of blood. As a result, the insect does not need to be exposed to feed many times; however, with the accentuated weight gain from recent feeding, locomotion is impaired, putting it in danger. To remedy this issue, triatomines have evolved the ability to process food quickly. For example, *R. prolixus* can remove approximately 50% of ingested blood within 3 hours after feeding (Martini et al., 2007). This is only possible because diuretic hormones and serotonin are released shortly after blood ingestion, causing the blood meal to concentrate and water to be eliminated from the body (Te Brugge et al., 2002). This could possibly explain why the transcripts related to endocrine functions of *R. neglectus* were the most diverse in the INT and SGs (**Figure 8**).

Functional coherence is evident when observing the predominant expression of transcripts related to the function performed by the analyzed tissue. The INT of triatomines has intense cellular catabolism and transport activity due to the digestive process (Ribeiro et al., 2014; Leyria et al., 2020b). During digestion, the insect must be able to extract all the nutrients it needs from the ingested blood, and then transport these molecules to the hemolymph to be used (Leyria et al., 2020b). Therefore, diverse findings of such transcripts are also expected in *R. neglectus* INT and SGs (**Figure 8**).

In other hematophagous arthropods, high amounts of enzyme transcripts are common in both SG and INT (Araujo et al., 2019). In ticks, enzymes in the SGs assist in the degradation of host tissues during the bloodmeal, while intestinal enzymes are directly related to the digestion of the diet (Araujo et al., 2019). The high numerical diversity in *R. neglectus* enzyme classes may be related to the high phosphorylation/dephosphorylation demands in physiological processes, peculiar to cell maintenance (Ribeiro et al., 2014). One example is inositol-3-phosphate synthase (Ino1), which participates in the production of inositol-containing compounds, including phospholipids (Majumder et al., 1997; Frej et al., 2016). The transcriptional increase (**Figure 9A**) suggests the increased demand of this enzyme during the elevation of metabolic rate/lipid synthesis after feeding (**Supplementary Figures 16AI, BI**).

As in other hematophages, several inhibitors are related to the insect feeding process, such as serine protease inhibitor proteins that participate by antagonizing coagulation and the complement system (Nevoa et al., 2018). Still, there is a predominance of several synthesizing enzymes composing the “other classes” group (**Figure 8**), whose transcripts are also present among those that are part of intrathecidal metabolic pathways (**Figure 12A**).

The peptidases identified here have an effect on the intestinal membrane, and their functions in triatomines still need to be better understood (Ribeiro et al., 2014; Ouali et al., 2020; Ouali et al., 2021). Noting that the action of peptidases, in general, is related to the process of protein breakdown, it is expected that the expression of enzymes in this group is still elevated after feeding. S10 peptidases are carboxypeptidases, a group of enzymes that promote the breakdown and removal of amino acids by the C-terminal portion (Aviles et al., 1993). However, S10s have already been identified in the gut of *R. prolixus* and possibly do not participate in the digestion process, but are important in the intestinal membrane (Ribeiro et al., 2014). On the other hand, although rarely described in triatomines, the S10 peptidase R4G841 has already been reported in the anterior and posterior guts of *R. prolixus* after hematophagy, which presumably participates in digestion and may be related to the activity of carboxypeptidases present in the midgut of triatomines. However, there are caveats for which it was found, whose physiological role still needs to be reviewed (Ouali et al., 2021). We identified an unknown S10 member that is most expressed after hematophagy in the gut of *R. neglectus*, and its role before the condition also needs to be investigated.

In addition to the S10 peptidase, two more unknown peptidases from the AA (which has aspartic residues) and S28 families were found to have their genes discretely more expressed in the feeding *R. neglectus* (**Figure 9A**). In *R. prolixus*, transcripts encoding aspartyl and cysteinyl proteases were found to have increased expression in the gut of fed insects (Ribeiro et al., 2014). The translational expression of some of these transcripts was later confirmed in the gut of *R. prolixus* after bloodmeal (Ouali et al., 2020).

The process of digestion in the insect requires high gene transcription for the synthesis of proteins that participate in blood metabolism (**Supplementary Figures 16BI, 17BI**) and to support the likely increase in epithelial cell division that begins after hematophagy (Ribeiro et al., 2014). Metabolic changes lead to alterations in post-transcriptional control, as is the case with iron metabolism (Hajdusek et al., 2009). In the *R. prolixus* INT there was also high expression of transcripts related to RNA processing genes after feeding, related to metabolic proteins being generated (Ribeiro et al., 2014). Putative proteins related to transcription processes and protein synthesis were also abundantly found in the midgut of *Triatoma infestans* (Buarque et al., 2011). Importantly, the highest overall diversity prevails in the INT (**Figure 8**), where higher metabolic activity occurs, than in the SGs after repast (Schwarz et al., 2014b).

Consistent with the transcriptional diversity of *R. neglectus* SGs and INT (**Figure 8**), the proteome of the gut of *R. prolixus* (Ouali et al., 2020) showed higher peptide abundance in the class of carbohydrate transport and metabolism related to the process of blood ingestion and digestion. Within this class, α-glucosidases are enzymes related to glycosidic metabolism and have already been related to the process of hemozoin formation, which is important for controlling the concentration of free heme that is released after hemoglobin digestion (Mury et al., 2009). The transcriptional expression of the secretory isoform of *R. neglectus* decreased by approximately half in INT after feeding (**Supplementary Figure 10B**), whereas the expression of the lysosomal isoform increased by 50% in INT and SGs (**Supplementary Table 2**). Within the glycolytic metabolic context, the increase in the precursor hormone neuroparsin 1 levels (**Figure 9A**) is related to changes in the developmental stages of insects and appears to be involved in insulin metabolism (Badisco et al., 2013). Thus, the increase in the levels of this hormone could explain the need for the increased activation of some metabolic pathways, but this hypothesis needs to be further investigated.

One of the molecules most transcriptionally expressed during this feeding period (**Figure 9A**) is Cyp6a14, which is associated with detoxification and resistance of the organism against insecticides (Mamidala et al., 2011), participates in the production of endogenous substances, such as some hormones (Feyereisen, 2006), and protects against oxygen reactants (Poupardin et al., 2010). As suggested by Ribeiro et al., the presence of these molecules in the INT may create a protective network against oxygen reactants that might be produced after hematophagy, as demonstrated for the species *R. prolixus* (Ribeiro et al., 2014). The increased expression of the Tat gene after hematophagy in *R. neglectus* (**Figure 9A**) was also observed in the *R. prolixus* INT (Ouali et al., 2021). This enzyme participates in the first reaction for the degradation of amino acids and is essential for tyrosine detoxification, an important process for survival in the repast of hematophagous arthropods, such as triatomines (Ribeiro et al., 2014; Sterkel et al., 2016).

The increase in p-myosuppressin levels, as seen transcriptionally in *R. neglectus* (**Figure 9A**), may be due to the physiological and endocrine changes that occur during this phase, as already seen in other triatomines (Ons et al., 2009; Ons et al., 2011; Lee et al., 2012; Ons, 2017). This suggests that high levels of this molecule may be involved in reduced intestinal and cardiac muscle contraction (Lee et al., 2012), which plays a role in maintaining physiological homeostasis in triatomines during the digestive process.

Digestion is also important for nutrient storage and supply to the future offspring during formation, and vitellogenin transcripts were the most highly expressed in SGs during this process (**Supplementary Figure 4A**). Vitellogenins have already been discovered at this site in *T. infestans* and *R. neglectus* 21 days after hematophagy (Santiago et al., 2020). However, the production of other molecules during this period is important for the proper and recurrent occurrence of hematophagy, such as lipocalins and nitrophorins, which are essential to facilitate blood flow from the vertebrate host to the insect at the bite site, and their expression is discussed in Section 4.6.

For the whole digestive and absorption processes to occur more effectively, it is known that SG and INT play important roles in insect humoral immunity, where reactive oxygen species, antiparasitic melanization, and antimicrobial peptides are produced (Ribeiro et al., 2014). Lysozymes and defensins have already been found in the transcriptomes of the gut of *R. prolixus* (Ribeiro et al., 2016), *T. infestans* (Buarque et al., 2013), and in the fat body and SG of *P. lignarius* (Nevoa et al., 2018). Thus, the highest transcriptional expression in the *R. neglectus* INT occurred in the fed insects, indicating that the microbiota could be under control and may did not overconsume the insect’s available nutrients (**Supplementary Figure 10A**). This also occurs in SGs (**Supplementary Figure 4A**), which could make it difficult for new microorganisms to enter the intestinal lumen. When ingested, this would result in a higher concentration of lysozymes in the food, which is important for the population control of bacteria that arrive with the bloodmeal.

### Transcriptional Expression in Infected Triatomines Is Regulated in Response to Survival of Tc

Transcriptional expression in infected triatomines is temporarily regulated in response to survival of Tc (**Supplementary Figures 4**, **10**), considerably in SGs for a short period (day 2) and in INT for a long period (until day 9). Among the several hypotheses to be mentioned hereafter that may favor the survival and transmissibility of Tc, the reduction of lysozyme concentration in the SGs may facilitate the contraction of new individuals and strains of Tc by transmission from the vertebrate host to the vector and favor nutritional competition in favor of the protozoan at INT. The initial reduction with subsequent re-establishment of lysozyme transcriptional expression suggests that the negative modulation is short-term and the insect feeding state on day 9 seems to be the only reason for the observed increase in lysozyme expression in FE + Tc, since lysozyme overexpression has already been evidenced in uninfected *R. prolixus*, even 9 and 12 days after blood feeding (Ribeiro et al., 2014). Lysozyme 1 was among the 20 most expressed transcripts on day 9 (**Supplementary Table 1**). However, in *T. infestans*, lysozyme 1 is transcriptionally expressed in infected INT as early as 1 day after infection, with a suggestive role in modulating Tc (Buarque et al., 2013).

This also appears to occur with the transcriptional expression of defensin C (**Figure 9B**), a molecule seen in *P. lignarius* SGs (Nevoa et al., 2018), *R. prolixus* INTs (Ribeiro et al., 2014), and *T. infestans* (Buarque et al., 2013), which are already known to participate in microbial control, both in ingested blood and at the site of the bite (Lopez et al., 2003; Ribeiro et al., 2014). In some portions of the INT, *T. cruzi* is able to positively regulate the expression of defensins, while in others, it does not occur (Waniek et al., 2011), showing a certain resistance of the insect against infection (Castro et al., 2012; Diaz et al., 2016).

It has been shown that different strains can interfere with the production of antimicrobial compounds and as a consequence, favor infectivity in the vector. This is the case for the DM28c strain (TcI) of *T. cruzi* that successfully developed in *R. prolixus*, while the Y strain (TcII) does not complete its life cycle. This is because the DM28c strain is able to control the local microbiota, activating the production of antibacterial compounds by the insect, especially against *Serratia marcescens*, a common bacterium in the gut of triatomines with a trypanolytic effect. On the other hand, strain Y does not achieve the same feat and ends up being the victim of the cytotoxic activity of the bacteria (Vieira et al., 2016). Studies related to the Colombian strain with defensin gene expression have not been found in the literature so far, and our results have led to the emergence of new investigations in this direction.

Pacifastin belongs to the family of serine protease inhibitors. Although identified in the SGs of *R. neglectus* after hematophagy, its activity has not yet been studied in this organ, but it is suggested to be involved in the immunity of the insect (Santiago et al., 2016). The increase in pacifastin levels in the INT of FE + Tc may indicate an attempted response of the vector to local infection, probably trying to be modulated by parasite colonization. The description of this protein in the *R. neglectus* gut also appeared for the first time in our study (**Supplementary Figure 10A**).

Kazal domain peptide Pr13a also showed increased transcriptional expression in SGs after feeding but was decreased in the presence of Tc (**Supplementary Figure 4A**). Proteins from these families are commonly associated with anticoagulation, vasodilation, and antimicrobial activities (Santiago et al., 2016). In contrast, in the *R. prolixus* INT, a Kazal-like inhibitor (RpTI) had gene overexpression after 3 h of *T. cruzi* infection with blood feeding (Soares et al., 2015). In *R. neglectus*, we observed that many protein genes behaved similarly to the Kazal domain peptide Pr13a in SGs, such as an I1 unassigned peptidase inhibitor and a venon protein kinase 1-like protein, which could also be explained by short-term interference from the parasite.

In INT, the increase in acetyl CoA synthetase levels (**Supplementary Figure 10A**) appears to be influenced by bacterial acetylation (Liimatta et al., 2018). This enzyme converts large amounts of acetate during bacterial growth into acetyl-CoA, which is an alternative carbon source for these microorganisms (Wolfe, 2005; Liimatta et al., 2018). After the hematophagy, it is common for flora to grow, which can impair Tc development. In this regard, the discrete transcriptional increase in Pp23a and diptericin levels (**Supplementary Figure 10A**) may assist in controlling microbial growth, as seen with serrulin in scorpion, a glycine-rich bioactive peptide with antimicrobial activity (Oliveira et al., 2018). Diptericin, a member of a family of glycine-rich antibacterials (Cudic et al., 1999) is present in dipteran hemolymph (Wu et al., 2018). However, Pp23a has not yet been observed in the *R. neglectus* INT and the transcriptional increase in these proteins observed here (**Supplementary Figure 10A**), for a short period, may be triggered by the presence of the parasite, possibly trying to control the development of the environment.

Given the presence of free radicals resulting from digestion, it is possible that the increase in the transcriptional levels of Sult1c4-like (**Figure 9B**) is also related to some process performed by the parasite to eliminate harmful compounds and be able to survive in the insect INT. This protein has been found previously in the *R. prolixus* INT, and Sult family proteins are able to metabolize nitrate compounds, favoring removal (Ribeiro et al., 2014). On the other hand, perhaps as a defense mechanism of *R. neglectus* when trying to keep the environment more oxidizable, increased transcriptional expression of a yet unknown T3 peptidase occurs (**Figure 9B**). T3 peptidases are fundamental in the regulation of metamorphosis (Wu and Lu, 2008), insecticide metabolism (Pickett and Lu, 1989), and in the degradation of glutathione, an important antioxidant for the cell and for Tc itself (Comini et al., 2005; Krauth-Siegel and Comini, 2008).

However, the flexibility of Tc in adapting to new adverse conditions is evident. The inorganic phosphate cotransporter similar isoform X1 apparently acts in nucleic acid and phospholipid synthesis, signal transduction, and energy metabolism (Dick et al., 2012) and is more transcriptionally expressed in the presence of Tc (**Figure 9B**). The growth of *Trypanosoma rangeli* is strongly dependent on the presence of Pi in the culture medium (Dick et al., 2010). Given the physiomorphological similarity between this parasite and Tc and the high identity (~ 98%) and high similarity (~ 99%) between their phosphate transporters (Dick et al., 2012), this may justify the need for increased Pi transport when the parasite is present in triatomine cells in order to meet the demands of both organisms.

In addition to behaving as a nutrient opportunist, Tc disrupts key enzymes and molecules in the digestive process, which would lead to nutrient availability in the favor of the triatomine. For example, it is possible that the presence of Tc promotes a reduction in the expression of the platelet antiaggregator (Ouali et al., 2021), Antigen-5 precursor (**Supplementary Figure 10A**), Obp-like, and cathepsin D in the INT of the vector (**Figure 9B**). This cathepsin has already been found in *P. lignarius* SGs (Nevoa et al., 2018) and *T. infestans* INT (Balczun et al., 2012), which are associated with protein metabolism and blood digestion. However, cathepsin D has already been shown to be transcriptionally positively regulated in INTs of infected *T. infestans* (Buarque et al., 2013) and *R. prolixus* (Borges et al., 2006), in contrast to that found in the *R. neglectus* SGs (**Supplementary Figure 4B**). This expression may differ depending on the triatomine species.

On the other hand, it appears that this process occurs in the short term. At times of food shortage arising from the final digestive process (day 9), perhaps disinhibition/reversal stimulus occurs, returning expression to higher levels, as is the case with cathepsin B (**Supplementary Table 2**). Proteases of this family are dominant in expression levels after blood ingestion (Ouali et al., 2020; Ouali et al.). However, this protein was among the 20 most expressed transcripts only in FE + Tc INT on day 9 (**Supplementary Table 1**). As in *R. neglectus*, there was also no difference in the expression of cathepsin B in the *R. prolixus* INT with or without Tc infection. However, this occurs 24 h after feeding (Ursic-Bedoya and Lowenberger, 2007).

The NPC1 (Niemann Pick C1) protein is evolutionarily well conserved among species and is related to cholesterol transport into cells, ensuring sufficient amounts for steroid hormone production (Huang et al., 2007; Danielsen et al., 2016). Lipid-carrying apolipophorins were identified in *R. prolixus* SG after 21 days of feeding (Santiago et al., 2020). In *R. neglectus*, the intestinal lipid metabolic pathway on day 2 was slightly more activated than in uninfected insects (**Supplementary Figures 16BI, BII**), which is perhaps important to ensure greater energy reserve availability to the parasite. However, the transcriptional expression of venom apolipophorin-like protein 1 and NPC1 lipid transporters was lower upon infection (**Supplementary Figure 10B**), which may impair the storage of this reserve to the vector.

Similar to cathepsins and lipid transporters, a similar process appears to occur with acid phosphatases (**Supplementary Figure 4A**) present in organs of high metabolism, such as in the SGs of *T. infestans*, *Panstrongylus megistus*, *R. neglectus*, and *R. prolixus*. They may be involved in compound secretion and rRNA transcription (Anhe et al., 2007), and these processes may be impaired in SGs.

After evaluating the transcripts of metabolic pathway subnetworks and systemic components, we observed that feeding can increase global gene expression even within the SGs, possibly by altering saliva production/composition (**Supplementary Figures 16**, **17**). However, Tc infection contributes to decreasing rather than increasing this expression, causing the functional impairment of the SGs during hematophagy. Perhaps this effect is reflected in the 12% lower intake of these triatomines. However, it should be further investigated, as different parasite concentrations and/or strains might better explain the blood volume ingested (Takano-Lee and Edman, 2002; Verly et al., 2020).

In INT, tissue repair processes occur with feeding and we observed increase in transcript levels similar to that of Venom cub domain protein 2 (Walker et al., 2017) during this period (**Figure 9A**). This protein has already been associated with developmental processes in insects (Bork and Beckmann, 1993). However, although the digestive component pathway appeared to be slightly more activated for a short time in the presence of Tc (**Supplementary Figure 17BII**), the aging pathway also behaved similarly, while the developmental, regenerative, and sensory pathways showed subtle decreases in activation. The impairment in *R. neglectus* intestinal repair and development in the presence of Tc may also be instigated due to the lower expression of two transcripts similar to proteins possibly related to cell replication processes, such as the H3v1-like protein and the X4 isoform of CREBB (**Figure 9B**). Histone H3v1 is highly conserved between species (Postberg et al., 2010), and in *Drosophila*, CREBB plays a critical role in early embryogenesis (Akimaru et al., 1997), although its physiological role remains unclear.

It appears that the presence of Tc altered the overall expression of proteins in both the INT and SG. Most transcripts with altered expression did not maintain the same expression profile over the long term (**Supplementary Figures 4-15**), as evidenced by the analyzed pathways (**Supplementary Figures 16**, **17**). The fact that, after a few hours, the trypomastigote turns into an epimastigote and thus becomes able to colonize the triatomine is an important factor that triggers physiological changes (Nogueira et al., 2015) and this may be responsible for the changes found here. Consistent with this, the reduced expression of p-myosuppressin upon infection (**Figure 9B**) suggests that intestinal contraction may be stimulated to release the infecting forms into the lumen and towards the external environment. In addition, the prolonged transcriptional increase in vitellogenin levels during infection (**Supplementary Figure 4A**) suggests increased nutrient availability to the vector progeny and even Tc. Moreover, a greater number of healthy offspring may transmit Tc to other vertebrates, maintaining the Chagas disease cycle.

### Tc Alters the Expression Synergism of Nitrophorin and Lipocalin Families, Potentially Impairing Hematophagy in *R. neglectus*

One group of proteins found abundantly in hematophage saliva is the kratagonist, which acts in a variety of ways to alter the enzymatic function of the protein to which they bind. The term is derived from the Greek Kratos, meaning to seize or bind, and they essentially function as agonist inhibitors (Ribeiro and Arcà, 2009; Andersen and Ribeiro, 2017). In this group, there are proteins of unique relevance in the transcriptomic study of triatomines, such as lipocalins and nitrophorins, as well as triabins, pallidipins, and procalins, their respective precursors, in addition to the yellow protein, found only in insects and bacteria and has been related to melanization. However, the functions of many of these molecules remain unknown (Ferguson et al., 2011; Kato et al., 2017b).

Both lipocalins and nitrophorins express moments near or during hematophagy and are involved in insect immunity (Santos et al., 2007; Ribeiro et al., 2014; Santiago et al., 2016; Nevoa et al., 2018; Ouali et al., 2021). They have activities related to vasodilation, anticoagulation, and platelet aggregation (Andersen et al., 2005). During feeding, they are abundant in the SGs, functioning as facilitators of this process, since they are injected into the host to optimize blood flow to the insect. In *R. neglectus*, we can highlight the evident increase in the transcriptional levels of lipocalin-like protein 4 as well as nitrophorins 7, 4B, 3A, and 1A precursors (**Figure 11A**).

The lipocalin group is numerous. In the SG and fat body of *P. lignarius*, 78 contigs encoding lipocalins have been found (Nevoa et al., 2018). In *R. prolixus*, 88 CDSs, including pallidipin and triabin, were found in this family (Ribeiro et al., 2014). In *T. dimidiata*, after 5, 12, and 24 days of fasting, lipocalins accounted for approximately 90% of the proteins found in the salivary proteome, and this diversity, even if apparently redundant, is key to the evolution of hematophagy (Santiago et al., 2018). In *R. neglectus* SGs, they have already been described and related to feeding success (Santiago et al., 2016). We found 83 relevant isoforms of lipocalin transcripts, in addition to 32 possible isoforms of nitrophorins and 5 of triabin transcripts in SGs using the multi-k-mer method (**Supplementary Figure 2A**).

Another molecule from the same family has already been identified in *R. neglectus* saliva (Santiago et al., 2016) is triabin. This molecule inhibits thrombin, an important serine protease in the coagulation cascade, leading to the inhibition of platelet aggregation and prolonging clotting time (Fuentes-Prior et al., 1997). On the other hand, pallidipin is related to the inhibition of platelet aggregation and works alongside other salivary anticoagulants (Noeske-Jungblut et al., 1994). Another molecule, procalin, is responsible for allergenic responses in saliva, as seen in *Triatoma protracta* (Paddock et al., 2001). However, its role in insects during blood feeding is still unknown (Ganfornina and Sanchez, 2013). Redundancy of proteins that perform similar or complementary functions is common in the saliva of hematophagous arthropods (Santiago et al., 2018) and this may also occur in *R. neglectus*. However, we found that the stimulation for the expression of pallidipin, procalin, and yellow-like proteins was lower in *R. neglectus* SGs and INT than the other kratagonists analyzed after feeding (**Figure 11**).

Nitrophorins have been reported in several triatomine sialotranscriptomes, including *R. prolixus* (Ribeiro et al., 2014; de Carvalho et al., 2017), *Triatoma rubida* (Ribeiro et al., 2012), *P. lignarius* (Nevoa et al., 2018), *P. megistus* (Ribeiro et al., 2015), and *R. neglectus* (Santiago et al., 2016). In INT, they can bind to nitric oxide (de Carvalho et al., 2017), prevent platelet aggregation (Zhang et al., 1998; Andersen et al., 2005), and modulate host-released defense molecules in the blood (Ribeiro and Walker, 1994).

In general, these kratagonists and their precursors demonstrate different expression patterns depending on the tissue, condition, and species evaluated. The transcriptional expression of several isoforms of these molecules in a distinct manner (**Figure 11**) suggests that feeding promotes an unknown protein synthesis pathway during hematophagy. For lipocalins, it is interesting to note that despite the term “precursor” used to certain molecules and curiously refer to a possible negative feedback between these proteins and those named without the suffix “precursor”, it is possible that there are interaction mechanisms between these proteins. These mechanisms cause one group of molecules to be more transcriptionally expressed at a given time, and the other group is more expressed, in order to maintain a certain function for a long period with the relay of expression of distinct molecules. It is possible that this occurs in the presence or absence of Tc, which also seems to impair this process.

Many molecules were less transcriptionally expressed in the presence of parasites. In the SGs (**Figure 11A**), this process occurred for a short period (day 2) with lipocalin-like 3, lipocalin-like 2, nitrorophorin 3A, and venom triabin-like 1 proteins. In INT (**Figure 11B**), this process occurred for a long period (until day 9) with all the kratagonists analyzed, except for the proteins pallidipin-like 1 precursor, procalin-like 1 precursor, triabin-like 1 precursor, triabin-like 3, which already presented low levels of transcriptional expression than the other molecules.

Interestingly, lipocalin 2 in humans is synthesized by immune cells when facing bacterial infections and acts to inhibit bacterial growth by disrupting the iron that the bacteria would use (Flo et al., 2004). Thus, if lipocalin-like 2 protein has the same function in triatomines, the reduction of iron in the *R. neglectus* INT would likely act in favor of Tc development at this site by inhibiting the growth of harmful or competing bacteria (**Figure 9B**). However, in SGs, the presence of Tc appears to have an inverse effect (**Figure 11A**).

However, some kratagonists in INT diverge from those found in the literature. In infected INT of *T. infestans* analyzed after 24 hours of feeding, there was an overexpression of transcripts for nitrophorins, while lipocalins did not show altered transcript expression after infection (Buarque et al., 2013). It is possible, therefore, that the downregulation of these molecules may occur from day 2 and not so briefly, since in the INT of uninfected *R. neglectus*, the transcriptional expression of lipocalin AI-4, 5, 6, and 7 precursors and nitrophorin 1, 2, and 3 precursors showed up to 64-fold increase on day 2 (**Figures 9B**, **11B**). Furthermore, it has already been observed that the protein levels of the triabin-like lipocalin 4 precursor increased significantly in the *R. prolixus* INT within 6 hours after hematophagy (Ouali et al., 2021).

The delay in reaching a higher level of expression is perhaps due to longer-lasting mechanisms of action, exemplified by nitrophorin 3 from SG, which is related to slower release of nitric oxide, especially at the end of feeding (Andersen et al., 2005). Lipocalin AI-5 is likely to have functional differences from the other lipocalins discovered so far (Andersen et al., 2005), but further research is needed to determine these activities, including their duration.

## Conclusions

The purpose of this study was to better understand the effects of feeding and Tc infection in INT and SGs on the physiology of *R. neglectus* by observing the expression of protein transcripts in these tissues. This is the first known study using triatomines that evaluated the permanence of transcriptional changes under such conditions, even days after feeding/infection. Considering the importance of these findings for the development of control strategies, the molecules found were discussed here according to their involvement in digestion and infection.

The numerous molecules present in the gut or in the SGs of hematophagous arthropods work in an integrated manner to promote feeding, adaptation to the environment, aggressive agents, and consequent survival of the species. Here, we sought to expand our knowledge of the proteins involved in different scenarios that alter the physiology of *R. neglectus*, such as starvation or blood feeding in the presence or absence of the pathogen *T. cruzi*.

Like other hematophagous arthropods, in triatomines of the species *R. neglectus*, lipocalins are among the most commonly found proteins in the SGs and INT under the conditions studied. Some proteins, on the other hand, have been reported for the first time in *R. neglectus*.

In general, the fasting state promotes the expression of many genes for proteins that maintain insects during the starvation period. On the other hand, the process triggered by the ingestion of blood also causes many modifications in the expression of genes in the SG and INT of insects. However, the presence of *T. cruzi* profoundly modified the gene expression pattern of the two different organs of *R. neglectus*.

We proposed some hypotheses that should be investigated further and serve as directions for future research to better understand the different conditions tested: fasting, blood feeding in the presence or absence of an arthropod-borne pathogen. As in other studies that showed an overview of new findings on the expression of molecules in various arthropod transcriptomes (Assumpcao et al., 2012; Ribeiro et al., 2012; de Carvalho et al., 2017; Kato et al., 2017a; Araujo et al., 2019; Coelho et al., 2021), future studies could analyze the up/downregulated targets of interest, their practical role, identify potential therapeutic targets for Chagas disease and determine if the transcriptional changes are correlated with protein expression *in vitro* and *in vivo*, stratified even in shorter periods of transcriptional observation in FA, FE and FE + Tc.

Given the high proportion of unknown/unmatched protein transcripts among the 20 most expressed (**Supplementary Table 1**) in both INT (31) and SG (22), a more detailed characterization of their functions and physiological roles in the context of feeding and infection with intratecidual Tc is essential, thus bringing information about the parasite-vector interaction process. The presence of Tc that leads to the impairment of cell development, metabolism, nutrition and immune response in SGs and INT, the decrease of defensin and lysozyme transcripts as well as lipocalin and nitrophorin isoforms point to investigate the interference in the expression of targets that may contribute to parasite resistance.

## Data Availability Statement

The raw sequencing data were deposited at the NCBI Sequence Read Archive under Bioproject No. PRJNA757456. ORF sequences and other related descriptions are in **Supplementary Material**.

## Ethics Statement

Under the number CAAE 80660417.1.0000.5154, the present work was approved by the Research Ethics Committee of the Federal University of Triângulo Mineiro.

## Author Contributions

All the authors were involved in the design of this study. TC-C, CO, and RT were involved in the study design, analyzed the data, and wrote the manuscript. TC-C, CB, JN, and GR performed the experiments. HF performed the RNA-seq. MM, MS, VR, CO, and SS participated in writing the manuscript. All authors commented on the manuscript and read and approved the final version of the manuscript.

## Funding

This work was supported by the Foundation for Research Support of the State of Minas Gerais (FAPEMIG) – Grant number RED-00313-16, National Institute of Science and Technology in Molecular Entomology (INCTEM) in partnership with National Council for Scientific and Technological Development (CNPq) – Grant number 465678/2014-9, and Coordination for the Improvement of Higher Education Personnel (CAPES; finance code 001).

## Conflict of Interest

The authors declare that the research was conducted in the absence of any commercial or financial relationships that could be construed as a potential conflict of interest.

The handling editor declared a past co-authorship with one of the authors CO.

## Publisher’s Note

All claims expressed in this article are solely those of the authors and do not necessarily represent those of their affiliated organizations, or those of the publisher, the editors and the reviewers. Any product that may be evaluated in this article, or claim that may be made by its manufacturer, is not guaranteed or endorsed by the publisher.
