# Supplementary material for: Salivary and Intestinal Transcriptomes Reveal Differential Gene Expression in Starving, Fed and Trypanosoma cruzi-Infected Rhodnius neglectus
Source: Front Cell Infect Microbiol. 2021 Dec 17;11:773357. doi: 10.3389/fcimb.2021.773357 (PMC8722679; doi:10.3389/fcimb.2021.773357)
Supplement: Supplementary file 13 [file DataSheet_13.pdf]

Supplementary Material

Supplementary Material

1 Supplementary Figures

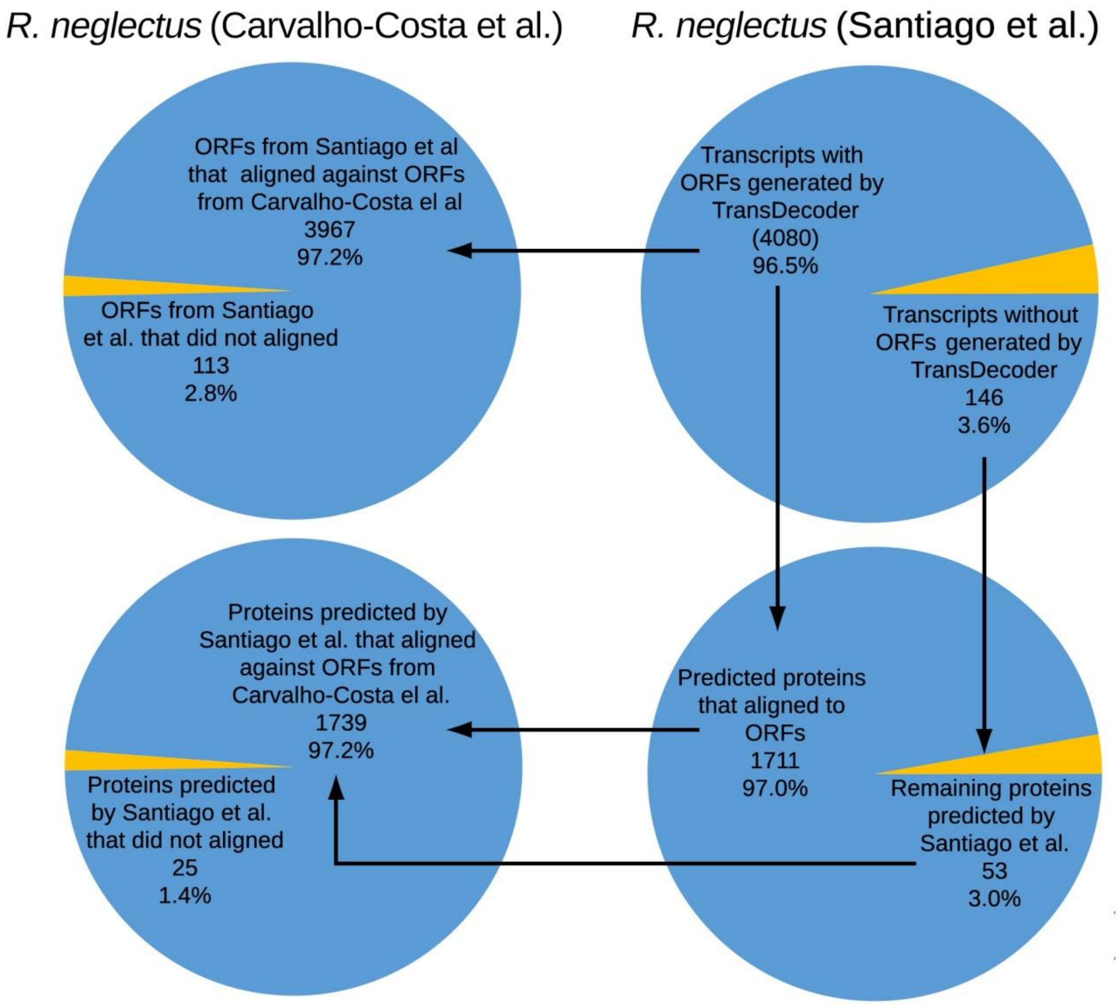

Supplementary Figure 1. Alignment between TransDecoder-generated ORFs and predicted protein sequences from *R. neglectus*. 4080 proteins were predicted by Santiago et al. (2016). Of these, 97.2% were also found in our work.

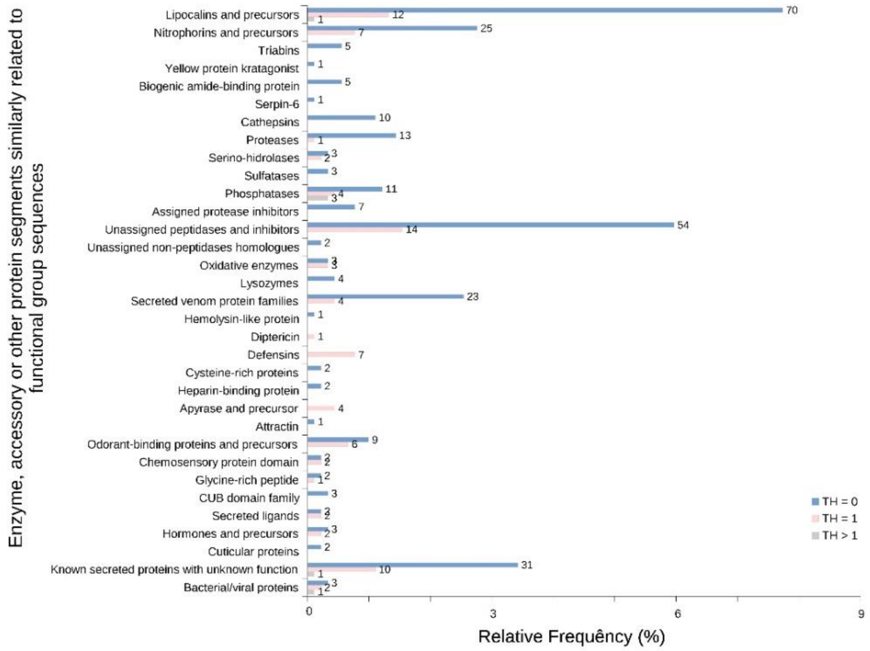

A

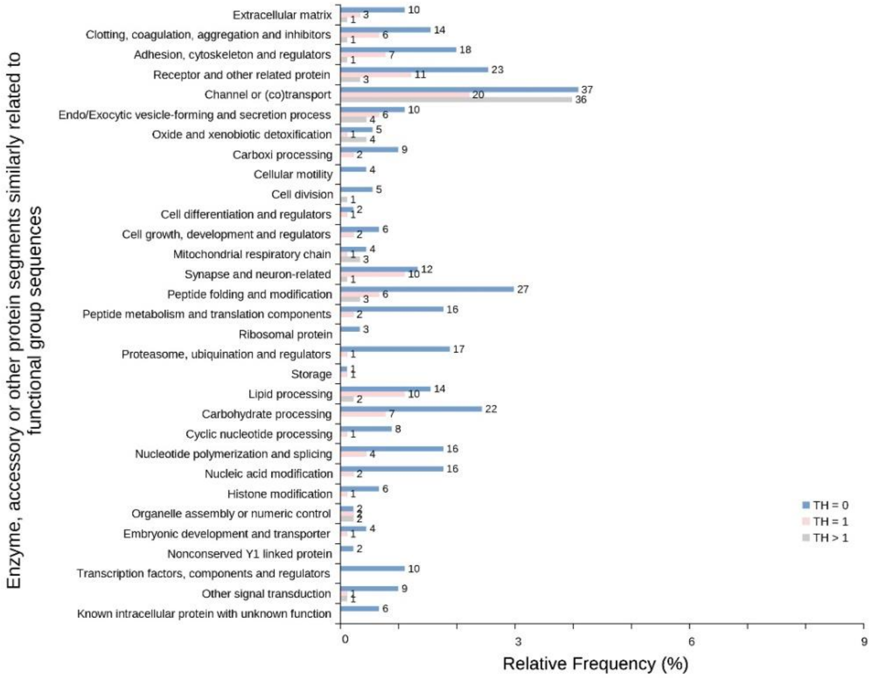

B

Supplementary Figure 2. Transcripts of known proteins SP+ in salivary gland secretomes and intestine. (**A**) Classes of proteins commonly present in arthropod secretomes. (**B**) Functional groups with probable secretable molecules (or of poorly specific prediction). *TH: number of transmembrane helices

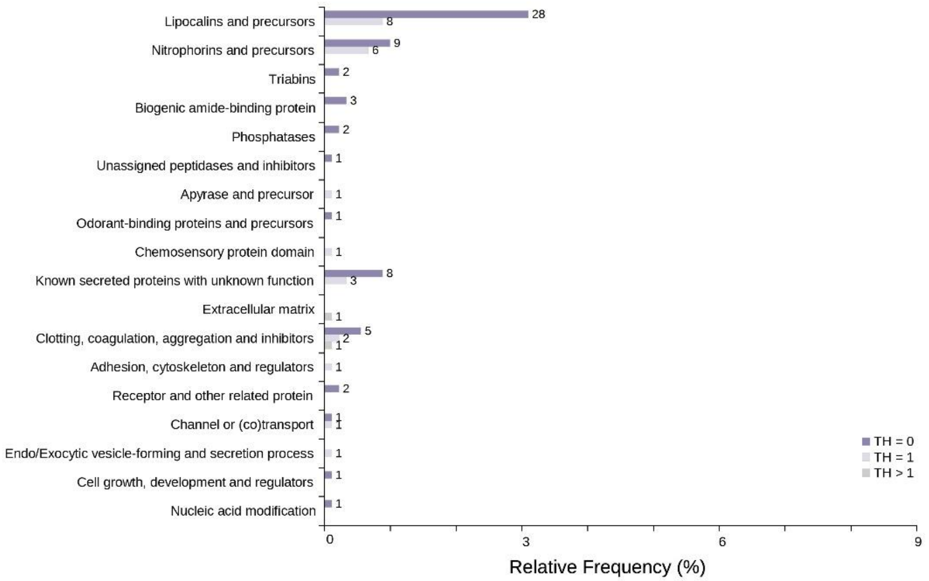

A

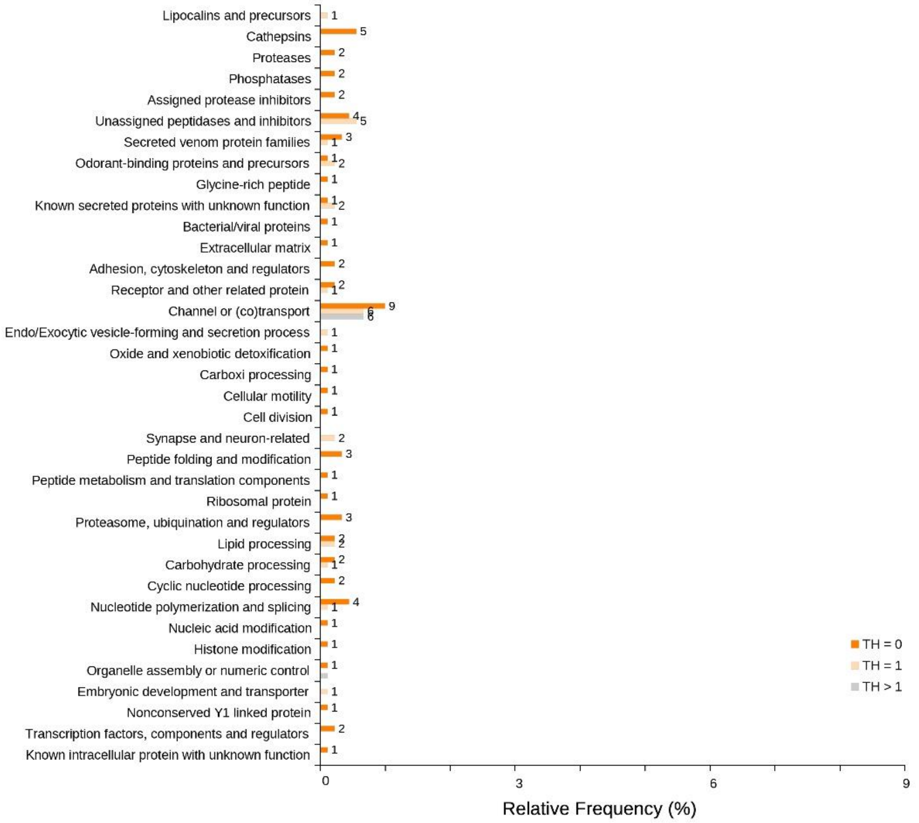

B

Supplementary Figure 3. Transcripts of known proteins SP+ present exclusively in each tissue type. (**A**) Present in the SGs and not in the INT. (**B**) Present in the INT and not in the SGs. *TH: number of transmembrane helices

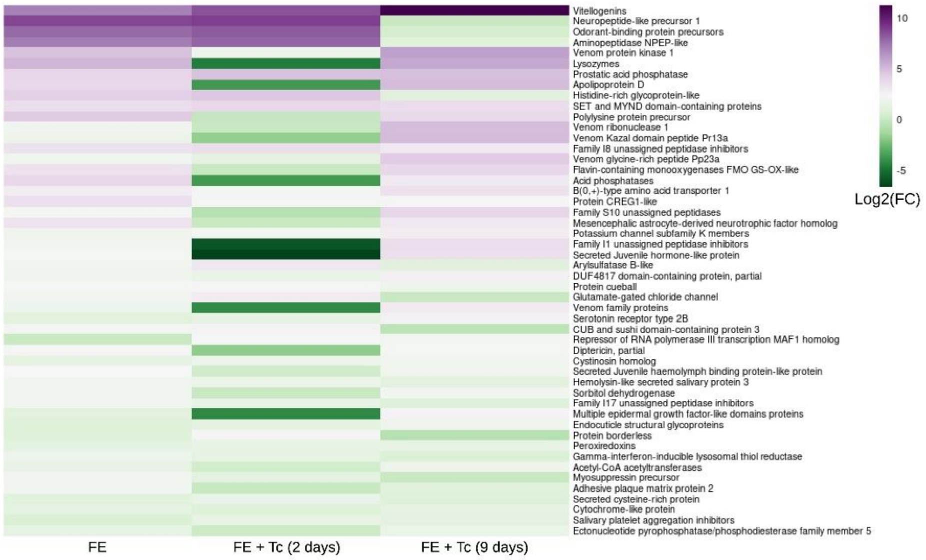

A

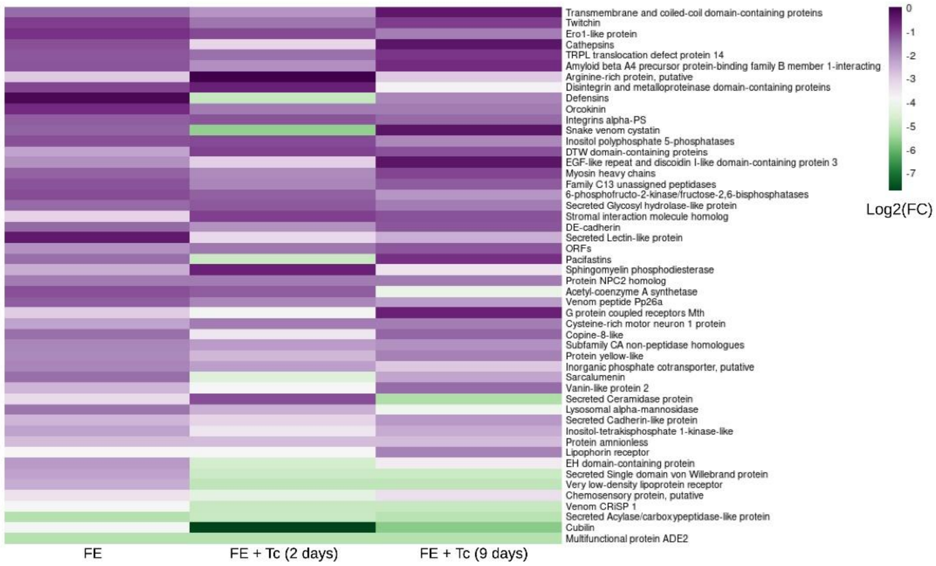

B

Supplementary Figure 4. Transcripts translatable into predicted protein as secreted in the salivary gland. (**A**) Top 50 upregulated transcript clusters per condition in relation to fasting; (**B**) Top 50 downregulated transcript clusters per condition regarding fasting. *FC: fold change.

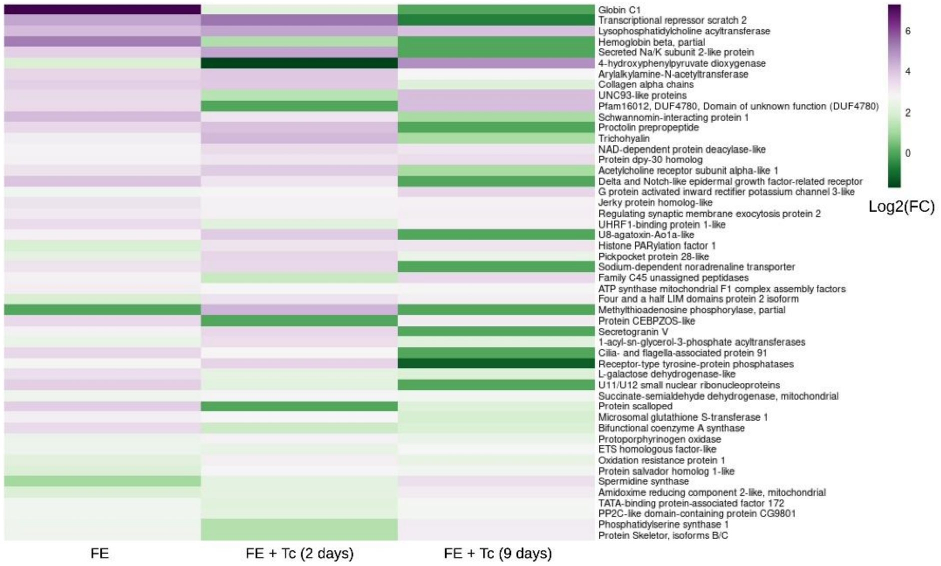

A

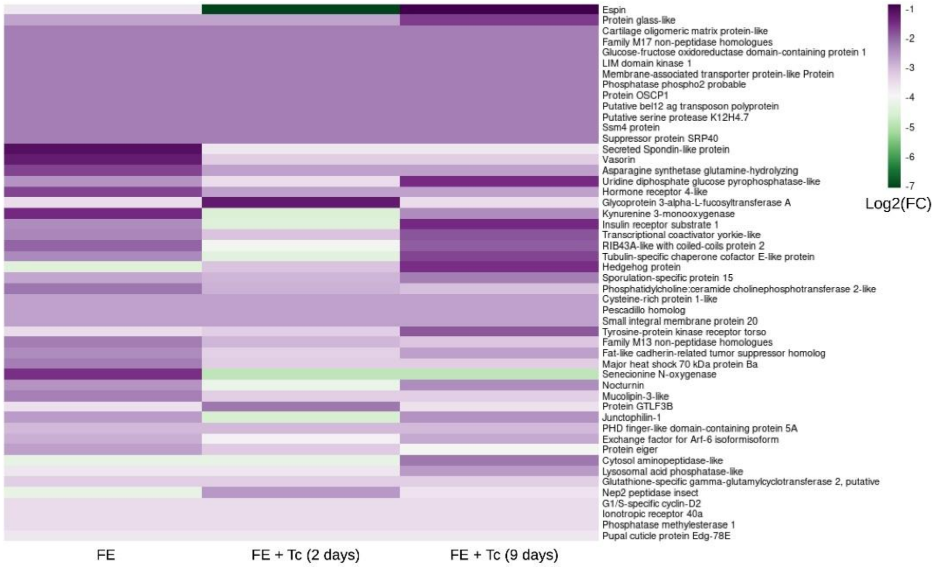

B

Supplementary Figure 5. Transcripts translatable into housekeeping proteins in salivary gland. (**A**) Top 50 upregulated transcript clusters per condition in relation to fasting; (**B**) Top 50 downregulated transcript clusters per condition in relation to fasting. *FC: fold change.

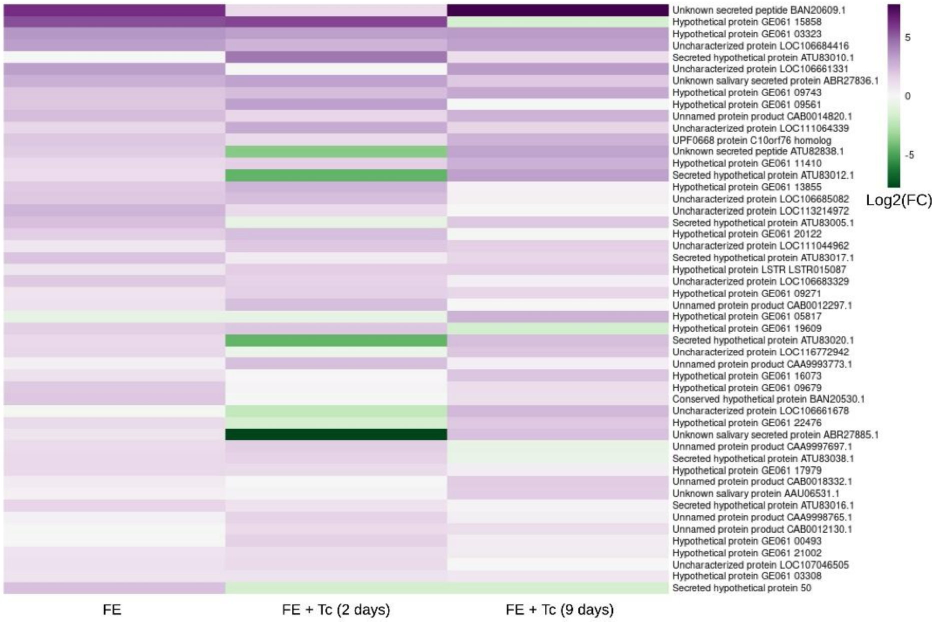

A

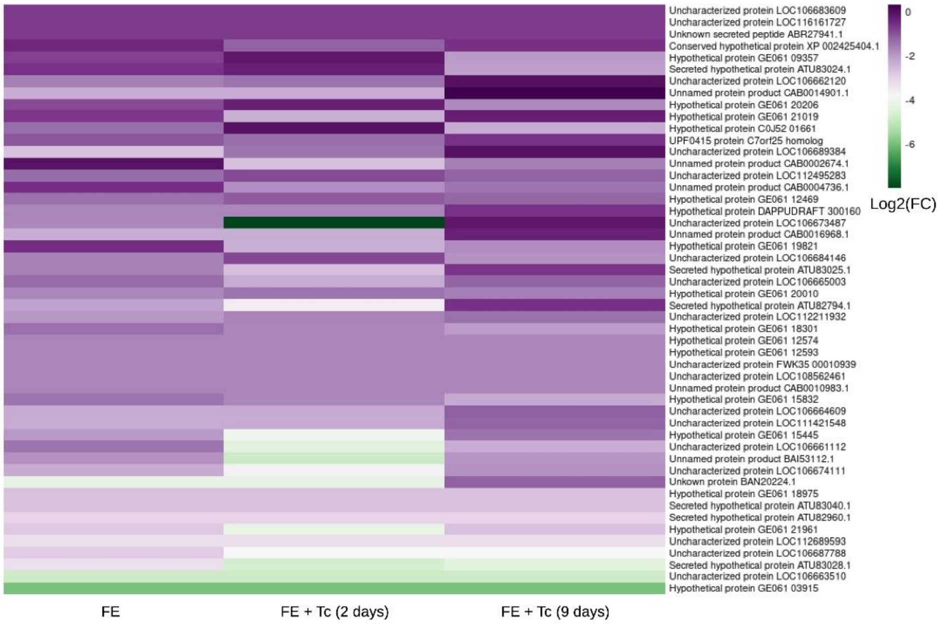

B

Supplementary Figure 6. Transcripts translatable into hypothetical/unknown secretable in the salivary gland. (**A**) Top 50 upregulated transcript clusters per condition in relation to fasting; (**B**) Top 50 downregulated transcript clusters per condition in relation to fasting. *FC: fold change.

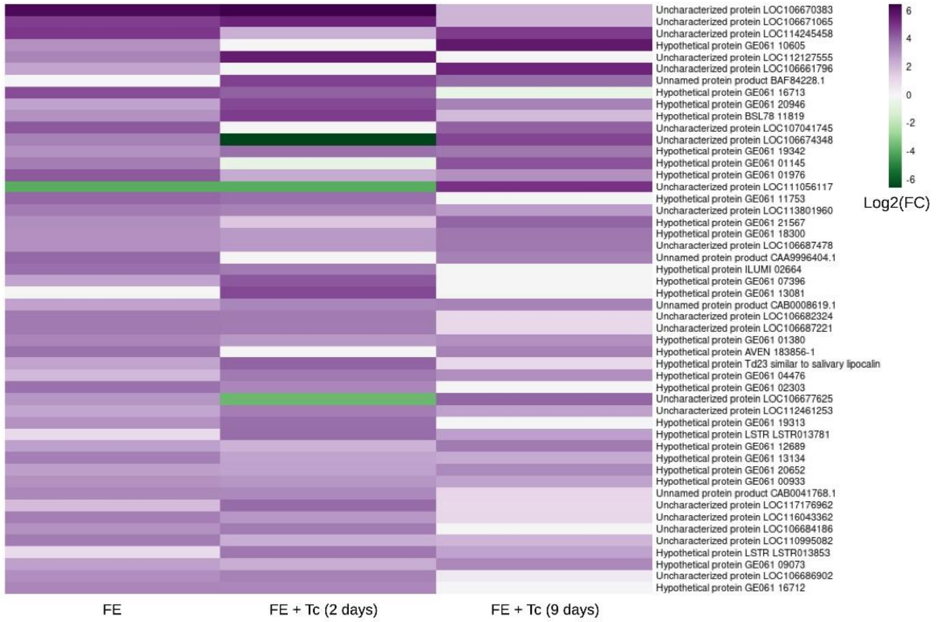

A

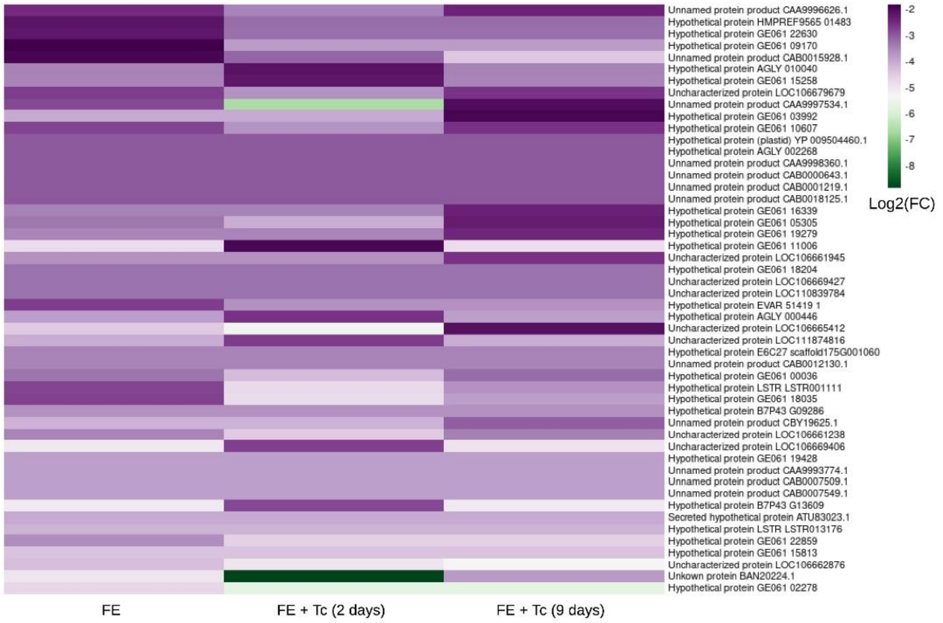

B

Supplementary Figure 7. Transcripts translatable into hypothetical/unknown non-secretable proteins in the salivary gland. (**A**) Top 50 upregulated transcript clusters per condition in relation to fasting; (**B**) Top 50 downregulated transcript clusters per condition in relation to fasting. *FC: fold change.

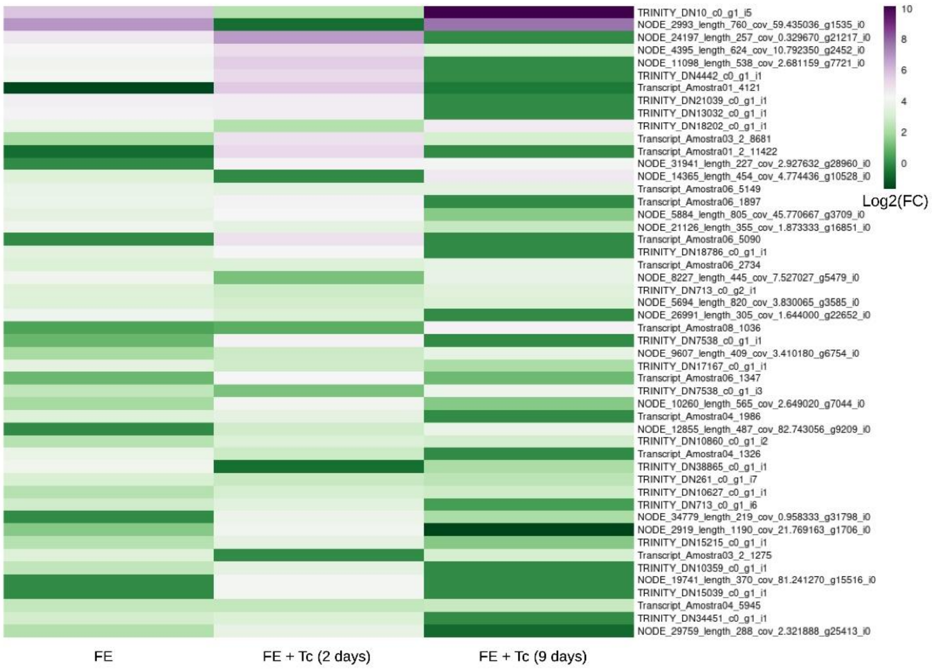

A

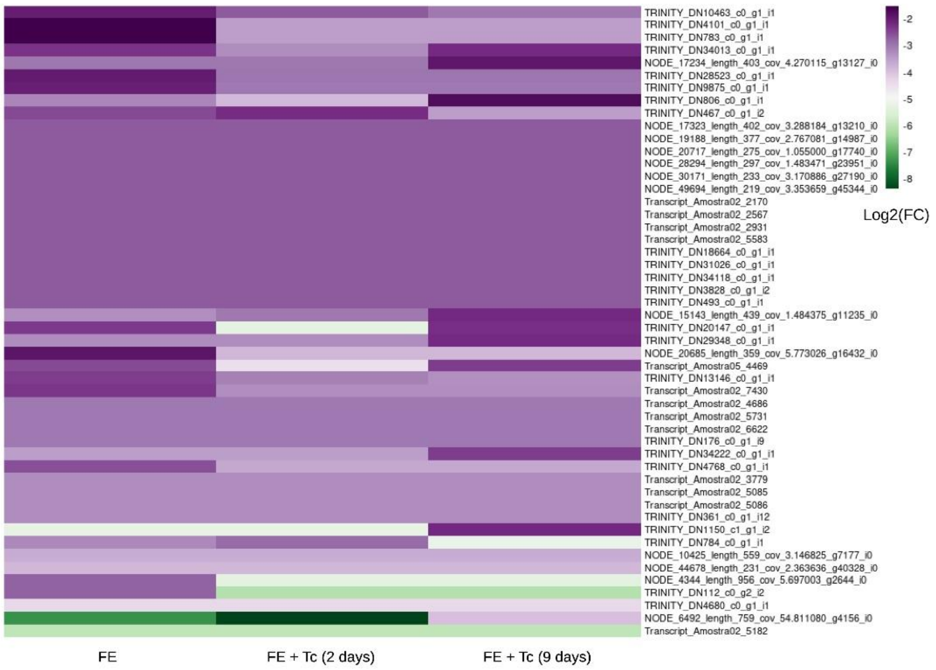

B

Supplementary Figure 8. Unmatched transcripts translatable into secretable proteins in the salivary gland. (**A**) Top 50 upregulated transcript clusters per condition in relation to fasting; (**B**) Top 50 downregulated transcript clusters per condition in relation to fasting. *FC: fold change.

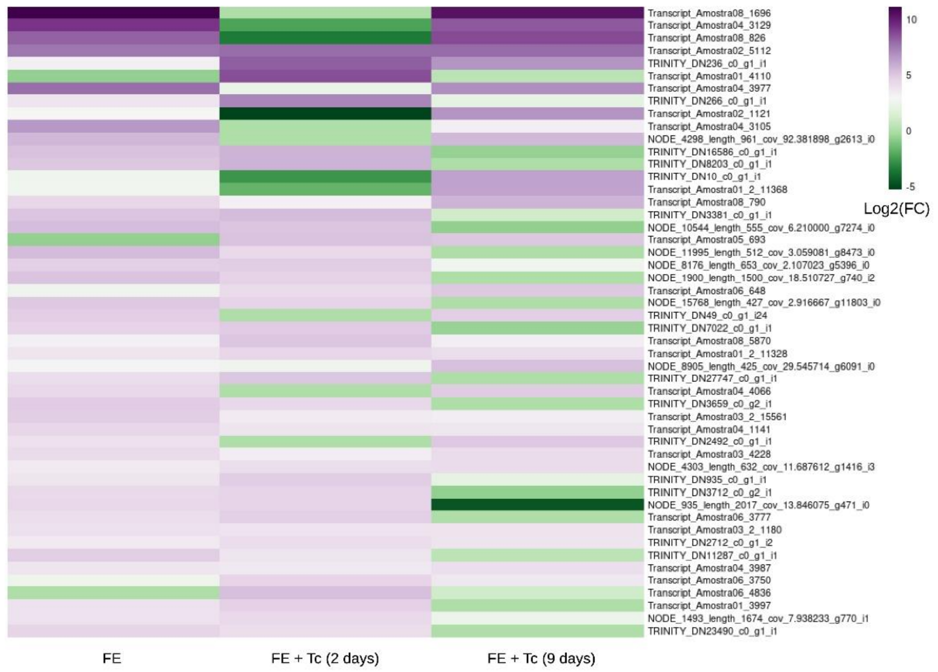

A

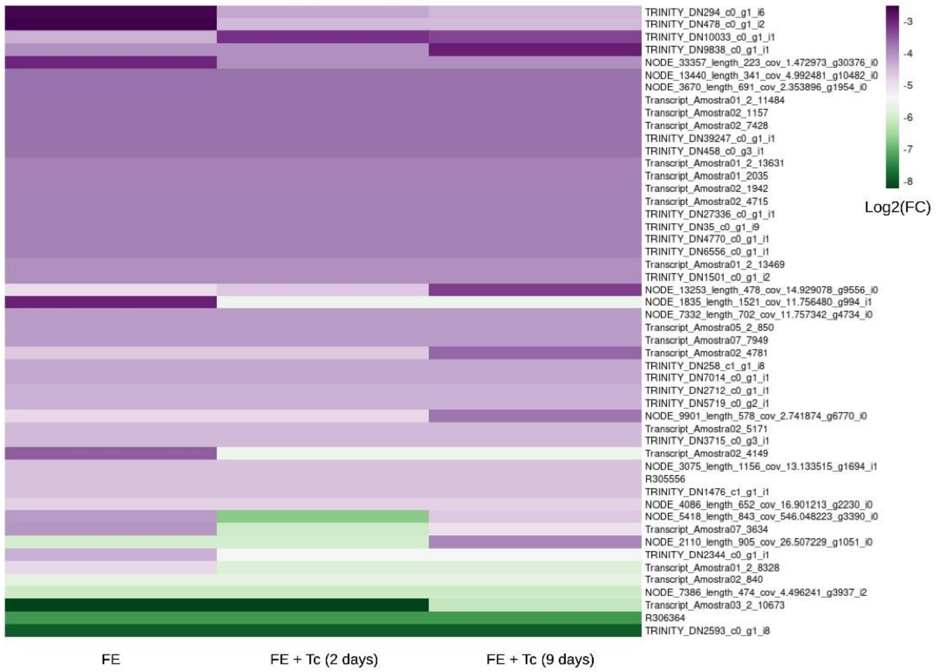

B

Supplementary Figure 9. Unmatched transcripts translatable into non-secretable proteins in the salivary gland. (**A**) Top 50 upregulated transcript clusters per condition in relation to fasting; (**B**) Top 50 downregulated transcript clusters per condition in relation to fasting. *FC: fold change.

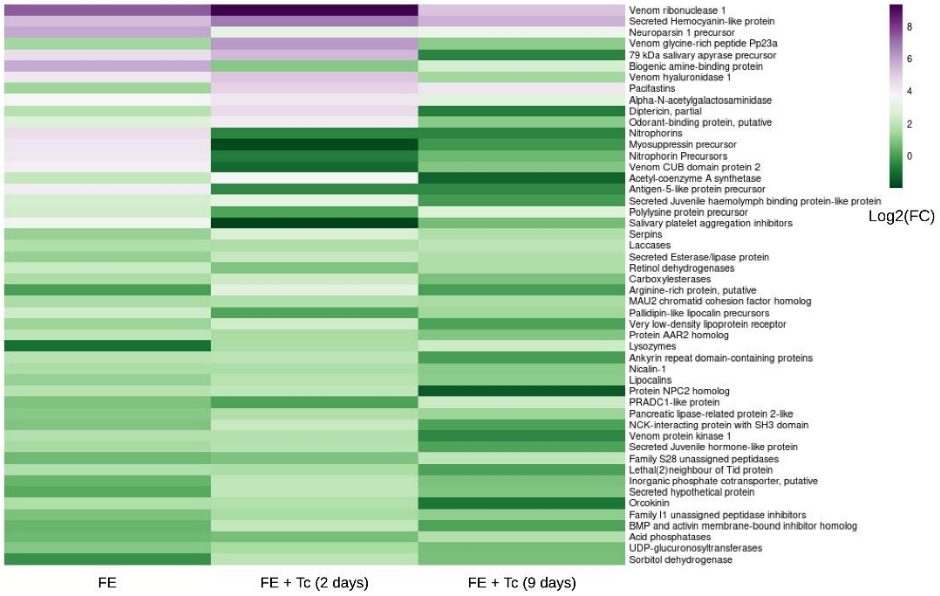

A

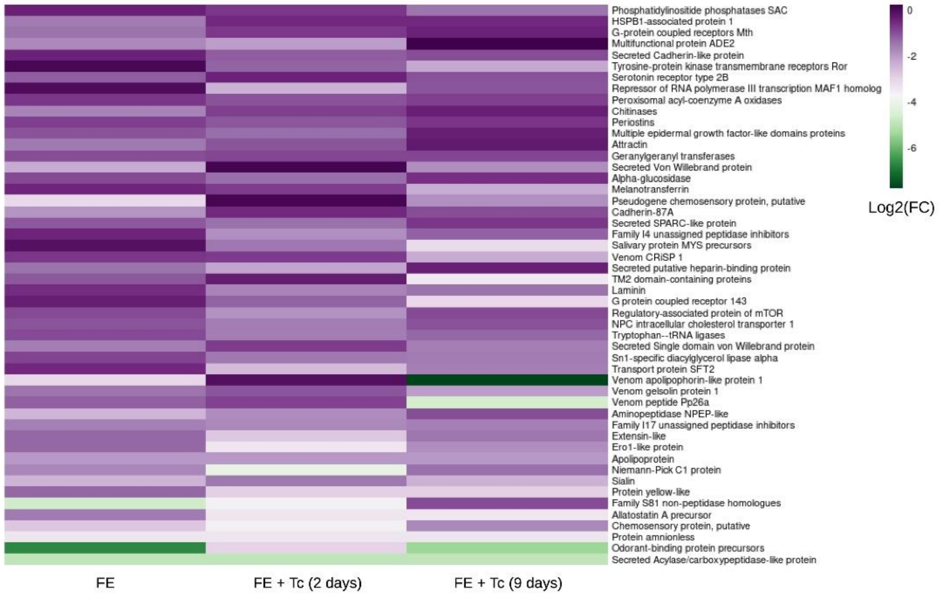

B

Supplementary Figure 10. Transcripts translatable into protein predicted as secreted in the intestine. (**A**) Top 50 upregulated transcript clusters per condition in relation to fasting; (**B**) Top 50 downregulated transcript clusters per condition in relation to fasting. *FC: fold change.

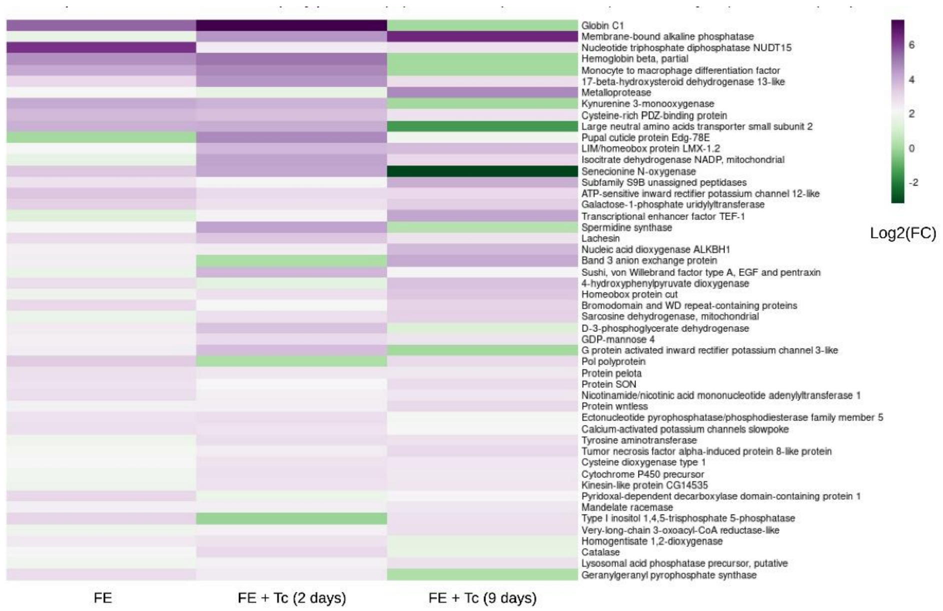

A

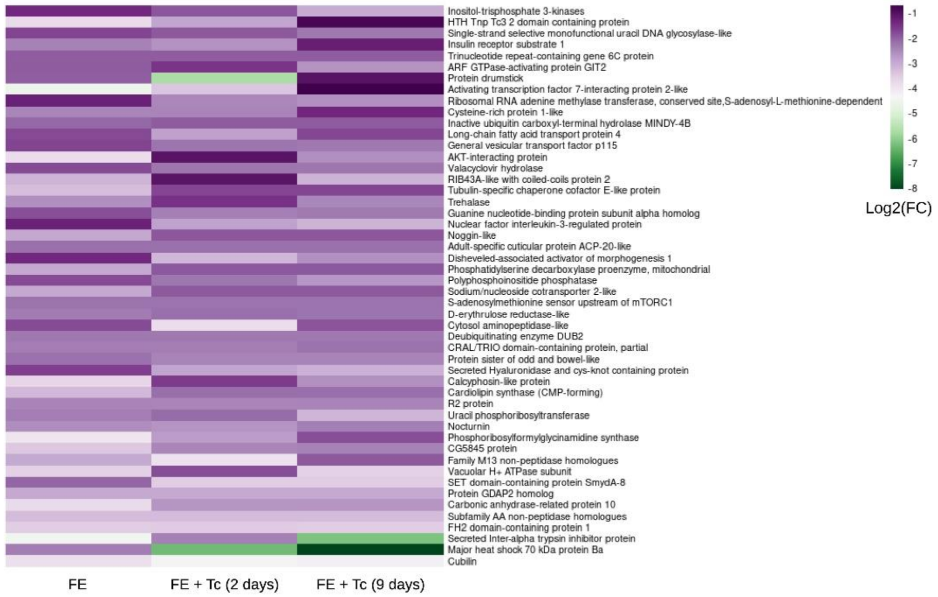

B

Supplementary Figure 11. Transcripts translatable into housekeeping proteins in intestine. (**A**) Top 50 upregulated transcript clusters per condition in relation to fasting; (**B**) Top 50 downregulated transcript clusters per condition in relation to fasting. *FC: fold change.

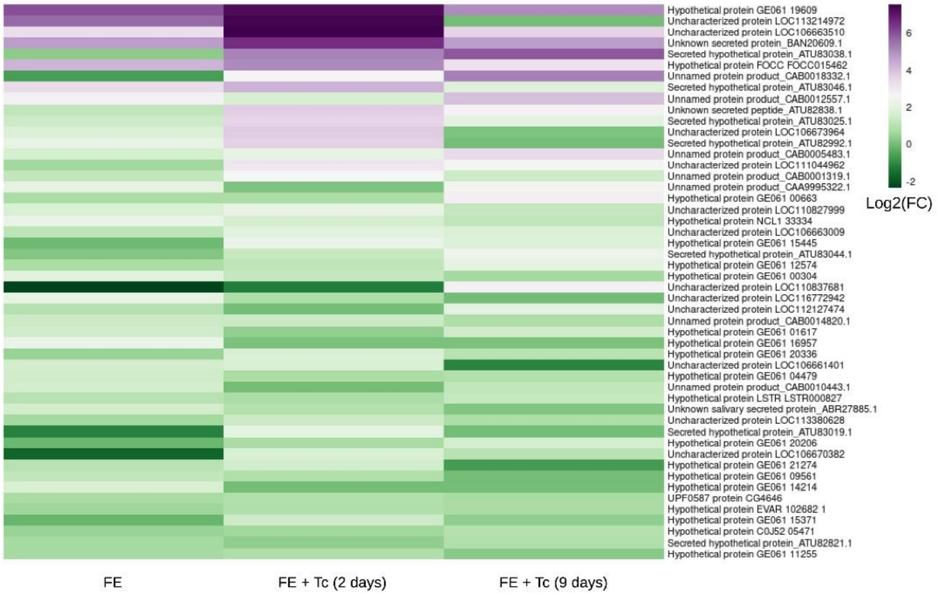

A

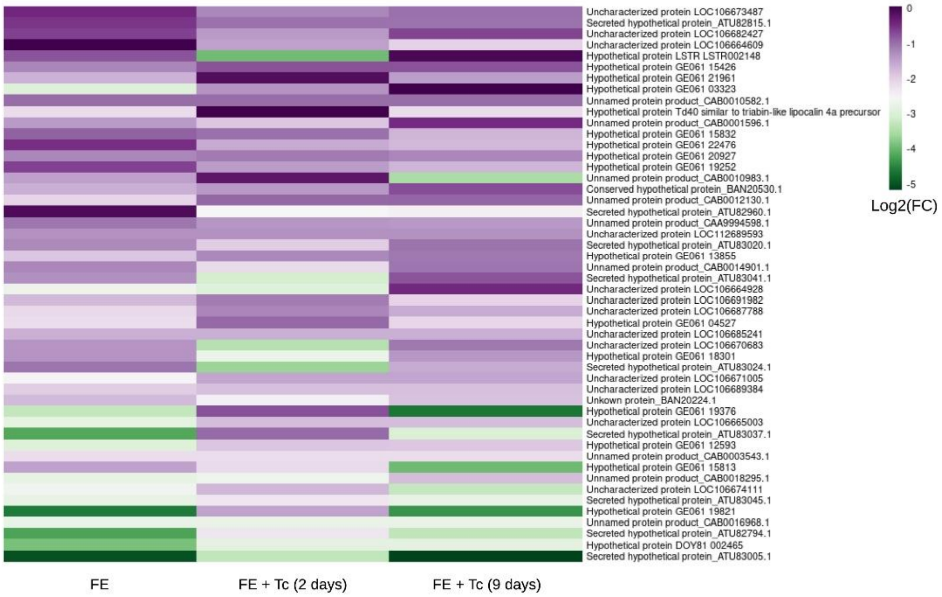

B

Supplementary Figure 12. Transcripts translatable into hypothetical/unknown secretable in the intestine. (**A**) Top 50 upregulated transcript clusters per condition in relation to fasting; (**B**) Top 50 downregulated transcript clusters per condition in relation to fasting. *FC: fold change.

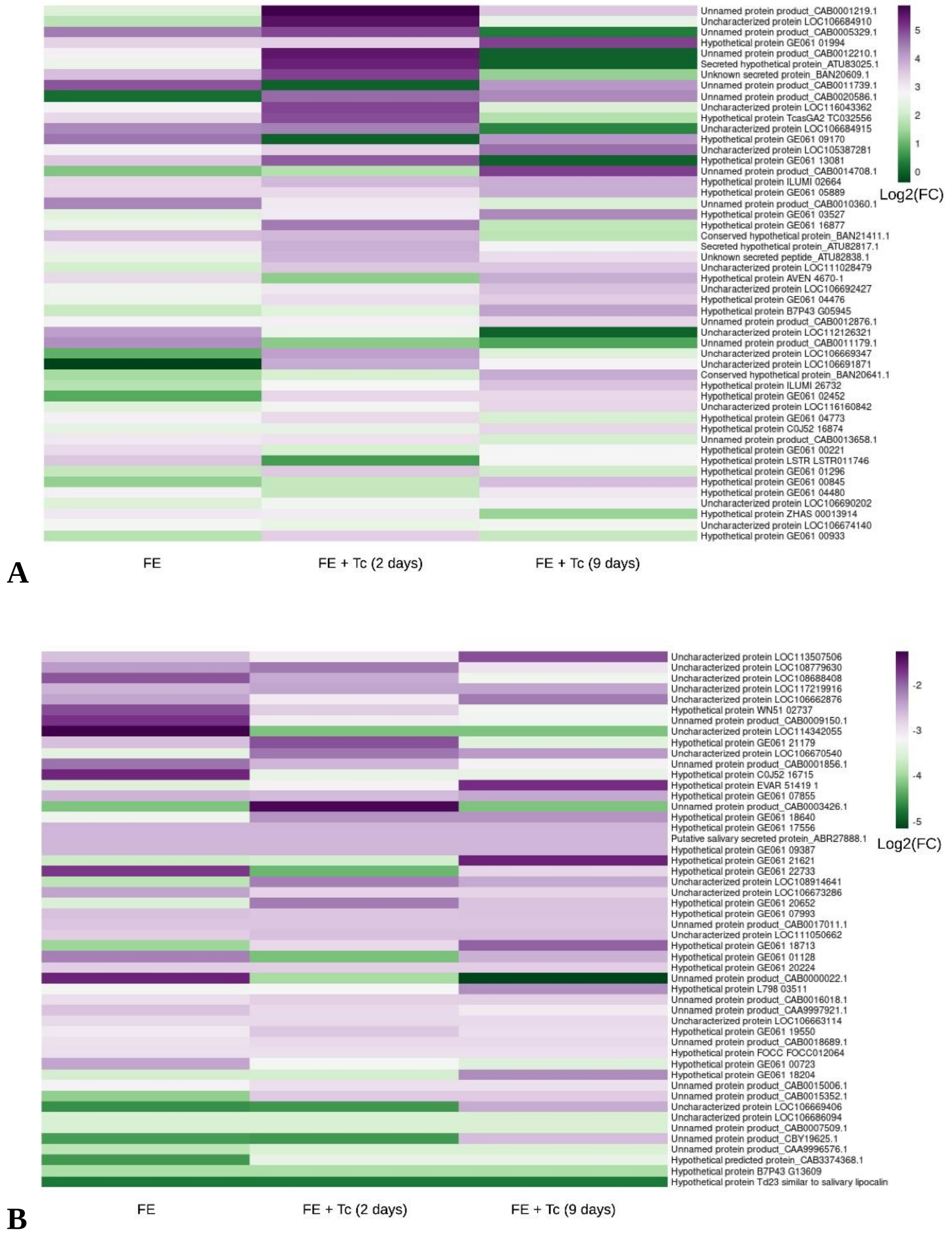

Supplementary Figure 13. Transcripts translatable in hypothetical/unknown non-secretable proteins in the intestine. (**A**) Top 50 upregulated transcript clusters per condition in relation to fasting; (**B**) Top 50 downregulated transcript clusters per condition in relation to fasting. *FC: fold change.

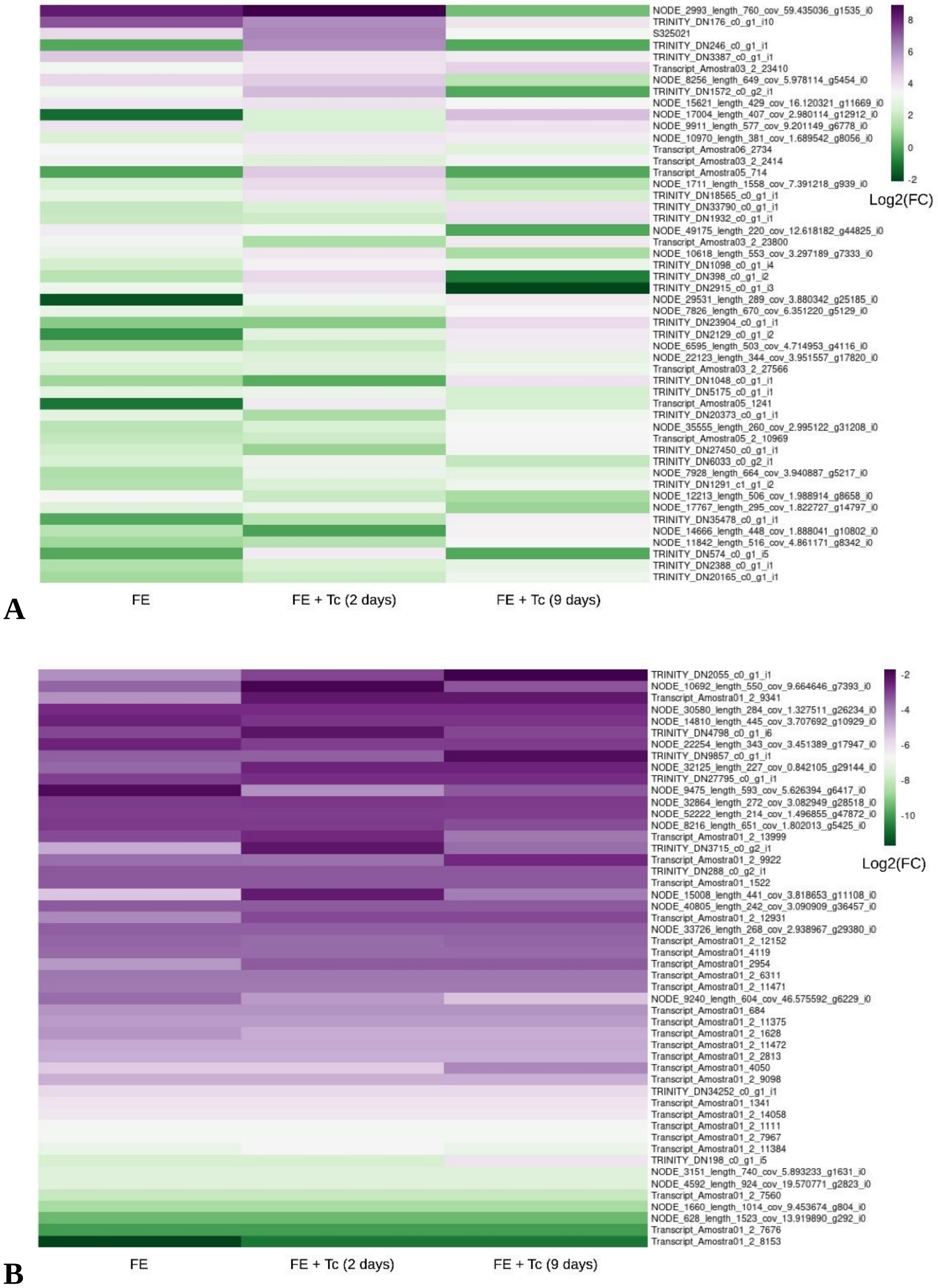

Supplementary Figure 14. Unmatched transcripts translatable in secretable proteins in the intestine. (**A**) Top 50 upregulated transcript clusters per condition in relation to fasting; (**B**) Top 50 downregulated transcript clusters per condition in relation to fasting. *FC: fold change.

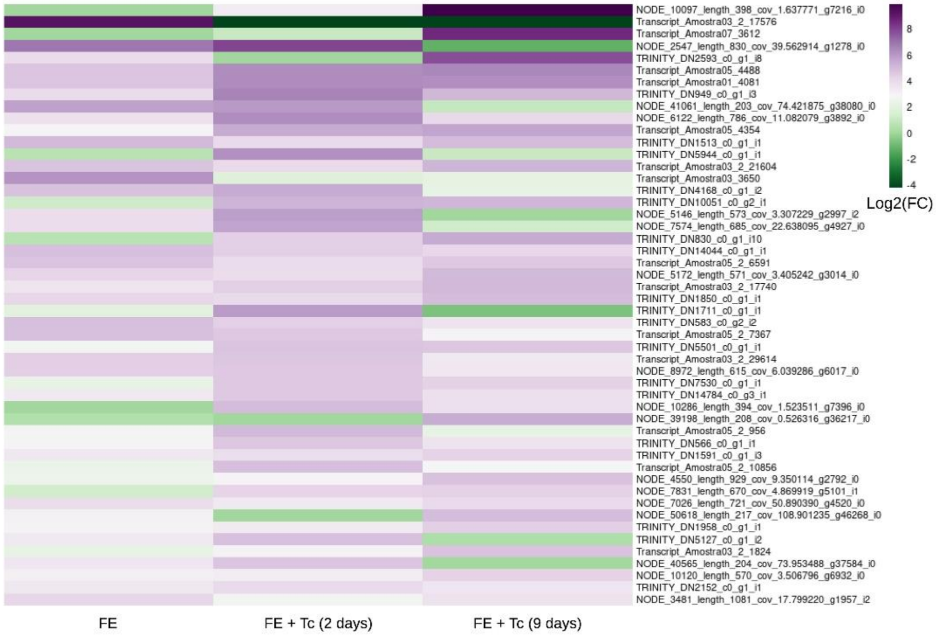

A

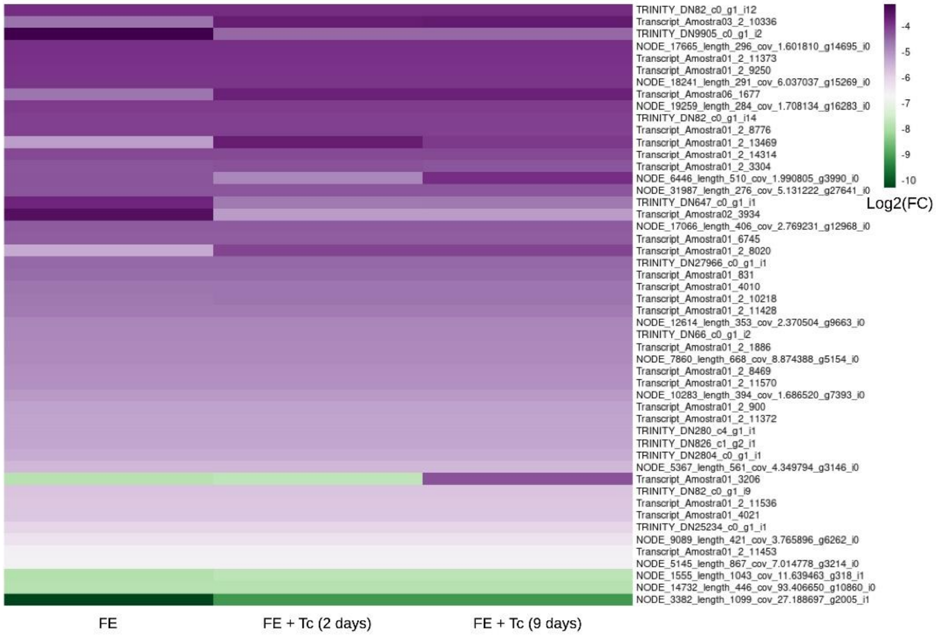

B

Supplementary Figure 15. Unmatched transcripts translatable in non-secretable proteins in the intestine. (**A**) Top 50 upregulated transcript clusters per condition in relation to fasting; (**B**) Top 50 downregulated transcript clusters per condition in relation to fasting. *FC: fold change.

AI

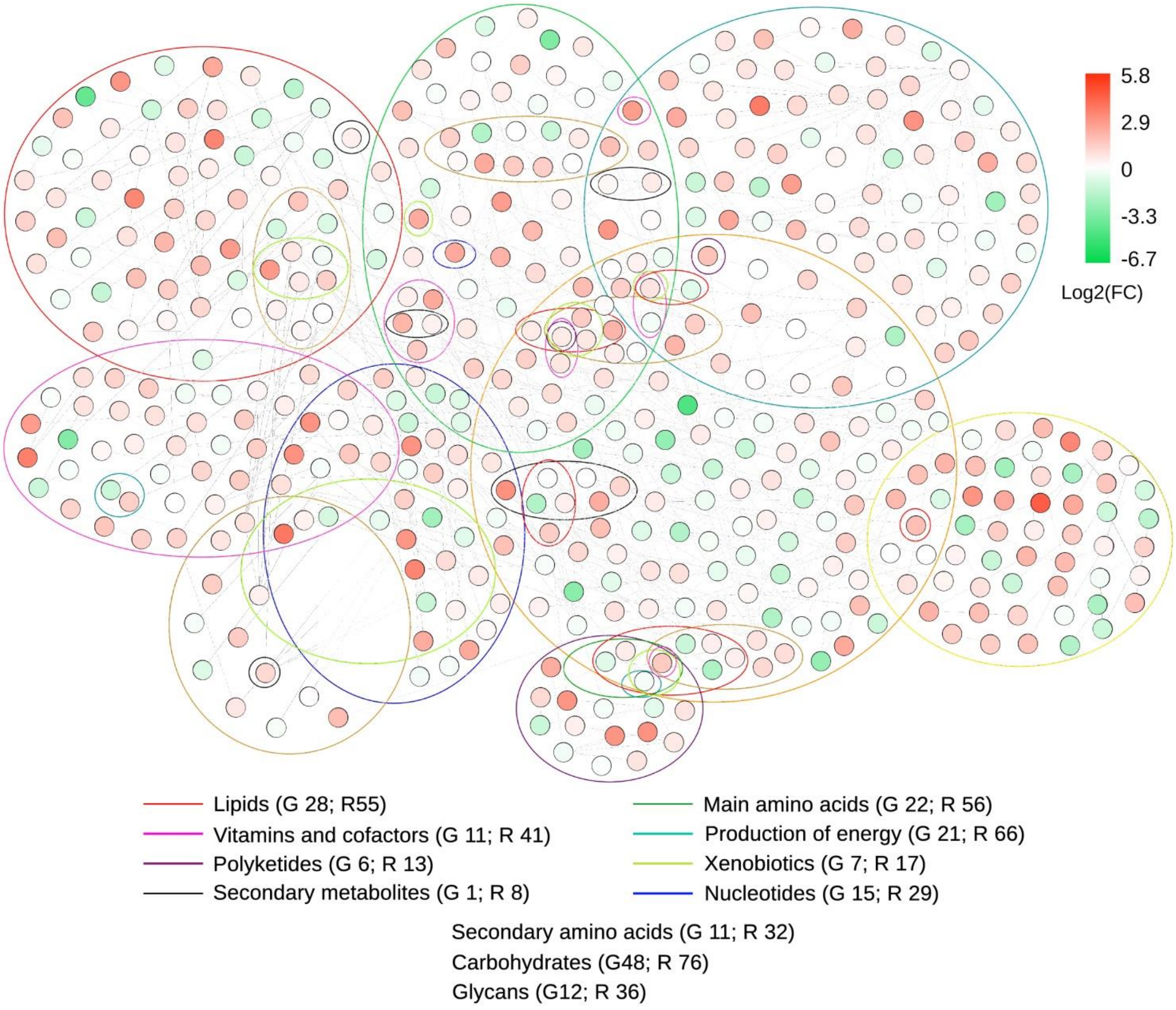
5.8
2.9
0
-3.3
-6.7
Log2(FC)
Lipids (G 28; R55)
Vitamins and cofactors (G 11; R 41)
Polyketides (G 6; R 13)
Secondary metabolites (G 1; R 8)
Main amino acids (G 22; R 56)
Production of energy (G 21; R 66)
Xenobiotics (G 7; R 17)
Nucleotides (G 15; R 29)
Secondary amino acids (G 11; R 32)
Carbohydrates (G48; R 76)
Glycans (G12; R 36)

AII

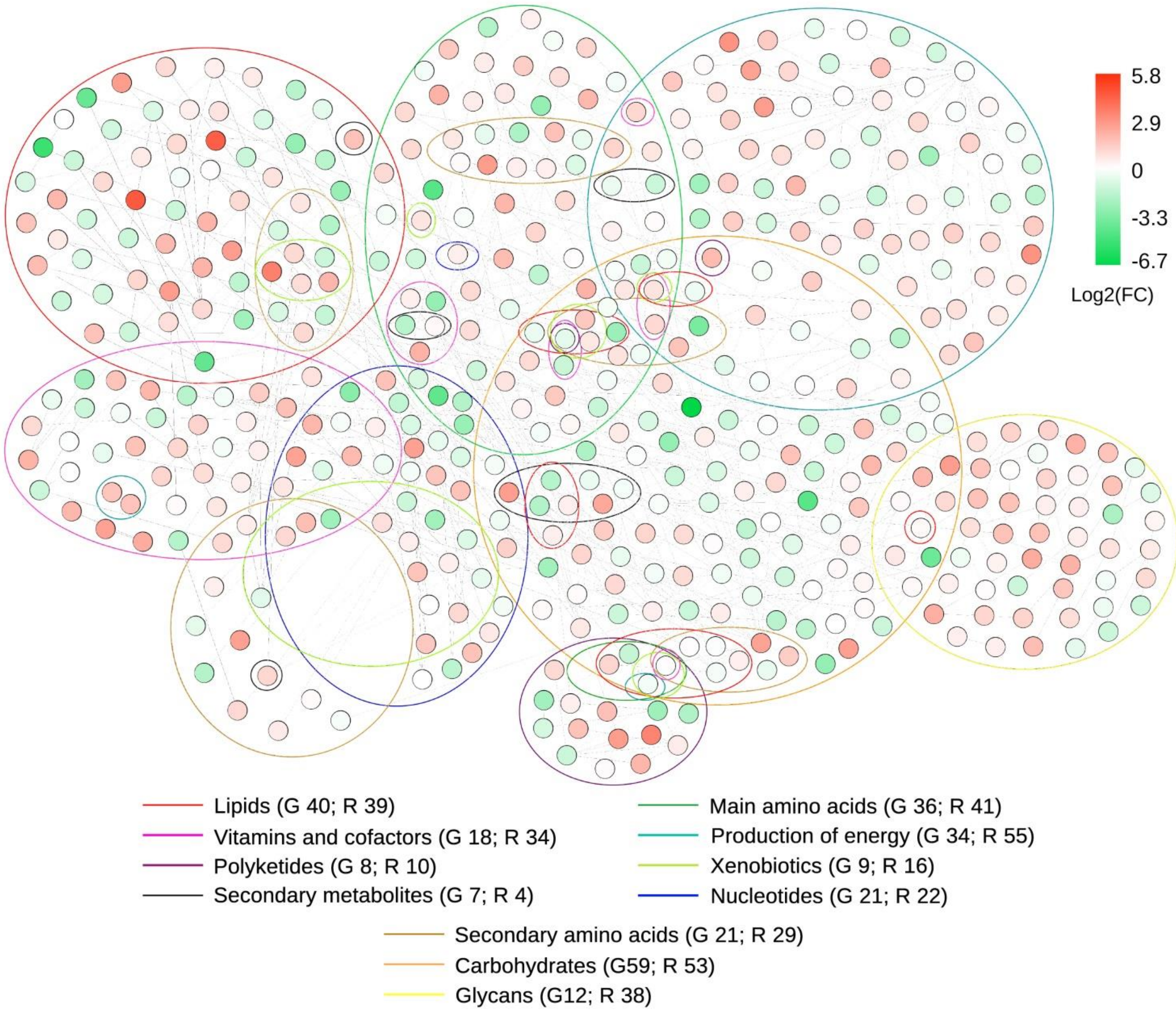

5.8
2.9
0
-3.3
-6.7
Log2(FC)
Lipids (G 40; R 39)
Vitamins and cofactors (G 18; R 34)
Polyketides (G 8; R 10)
Secondary metabolites (G 7; R 4)
Main amino acids (G 36; R 41)
Production of energy (G 34; R 55)
Xenobiotics (G 9; R 16)
Nucleotides (G 21; R 22)
Secondary amino acids (G 21; R 29)
Carbohydrates (G59; R 53)
Glycans (G12; R 38)

AIII

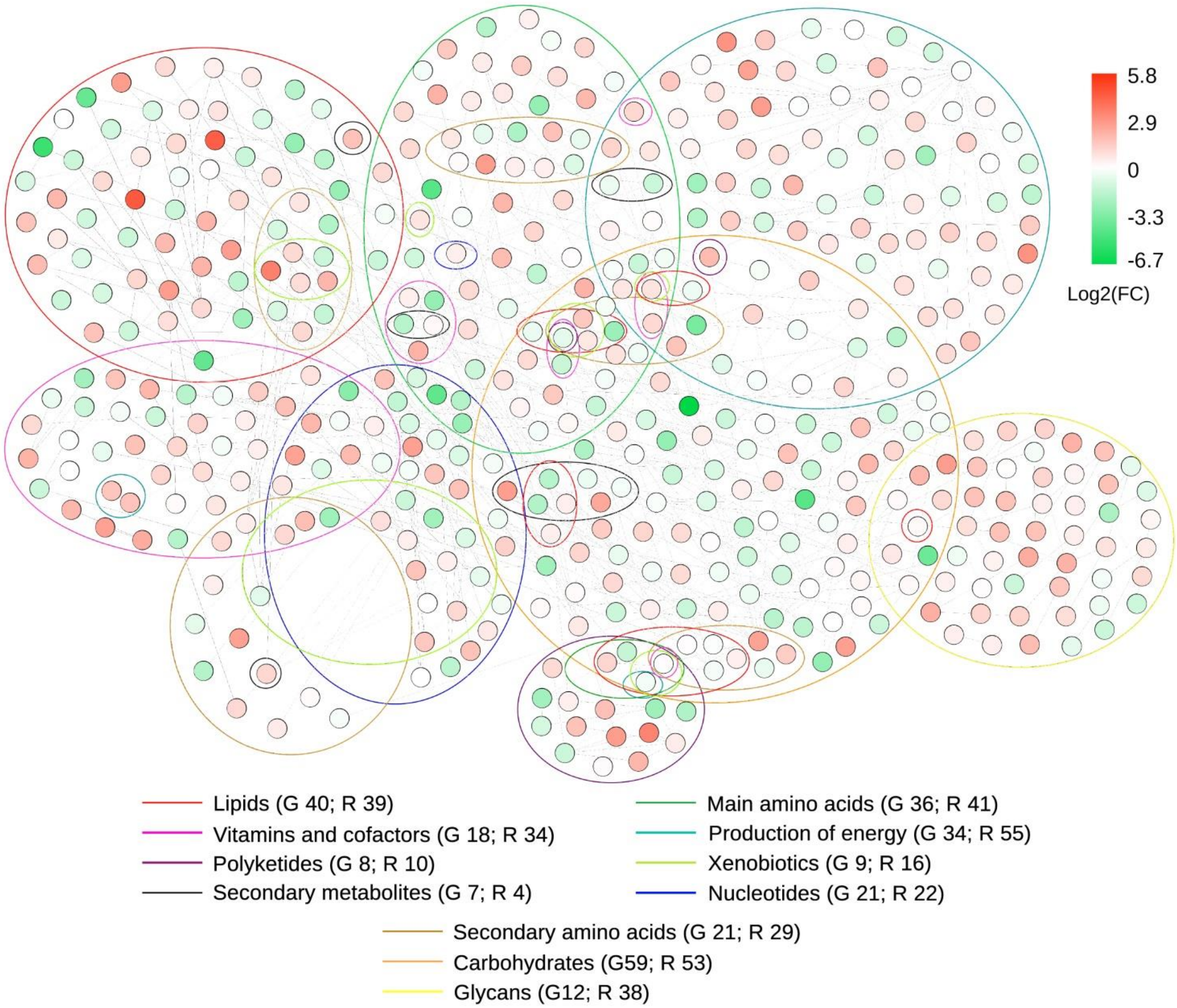
5.8
2.9
0
-3.3
-6.7
Log2(FC)
Lipids (G 40; R 39)
Vitamins and cofactors (G 18; R 34)
Polyketides (G 8; R 10)
Secondary metabolites (G 7; R 4)
Main amino acids (G 36; R 41)
Production of energy (G 34; R 55)
Xenobiotics (G 9; R 16)
Nucleotides (G 21; R 22)
Secondary amino acids (G 21; R 29)
Carbohydrates (G59; R 53)
Glycans (G12; R 38)

BI

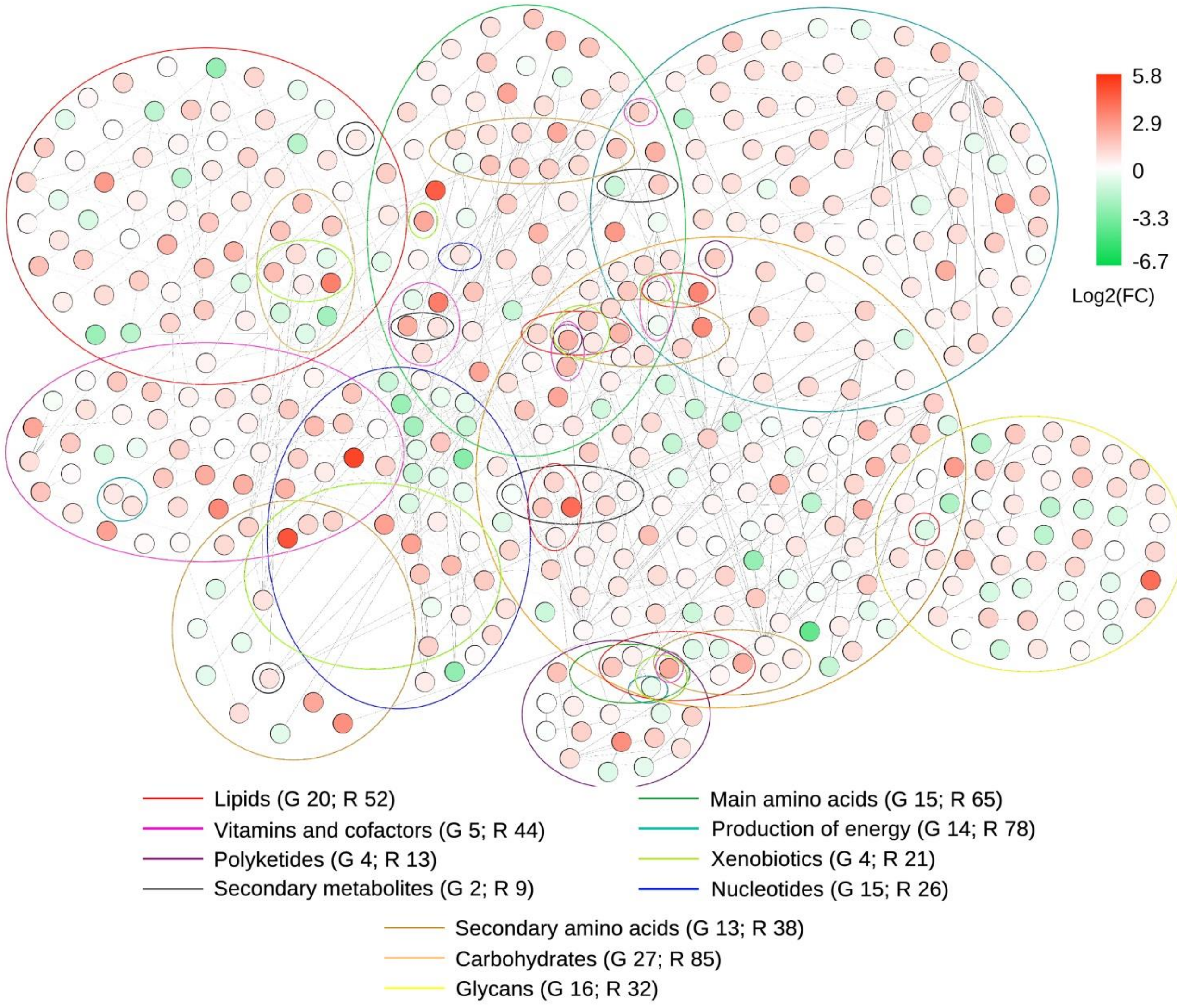
5.8
2.9
0
-3.3
-6.7
Log2(FC)
Lipids (G 20; R 52)
Vitamins and cofactors (G 5; R 44)
Polyketides (G 4; R 13)
Secondary metabolites (G 2; R 9)
Main amino acids (G 15; R 65)
Production of energy (G 14; R 78)
Xenobiotics (G 4; R 21)
Nucleotides (G 15; R 26)
Secondary amino acids (G 13; R 38)
Carbohydrates (G 27; R 85)
Glycans (G 16; R 32)

BII

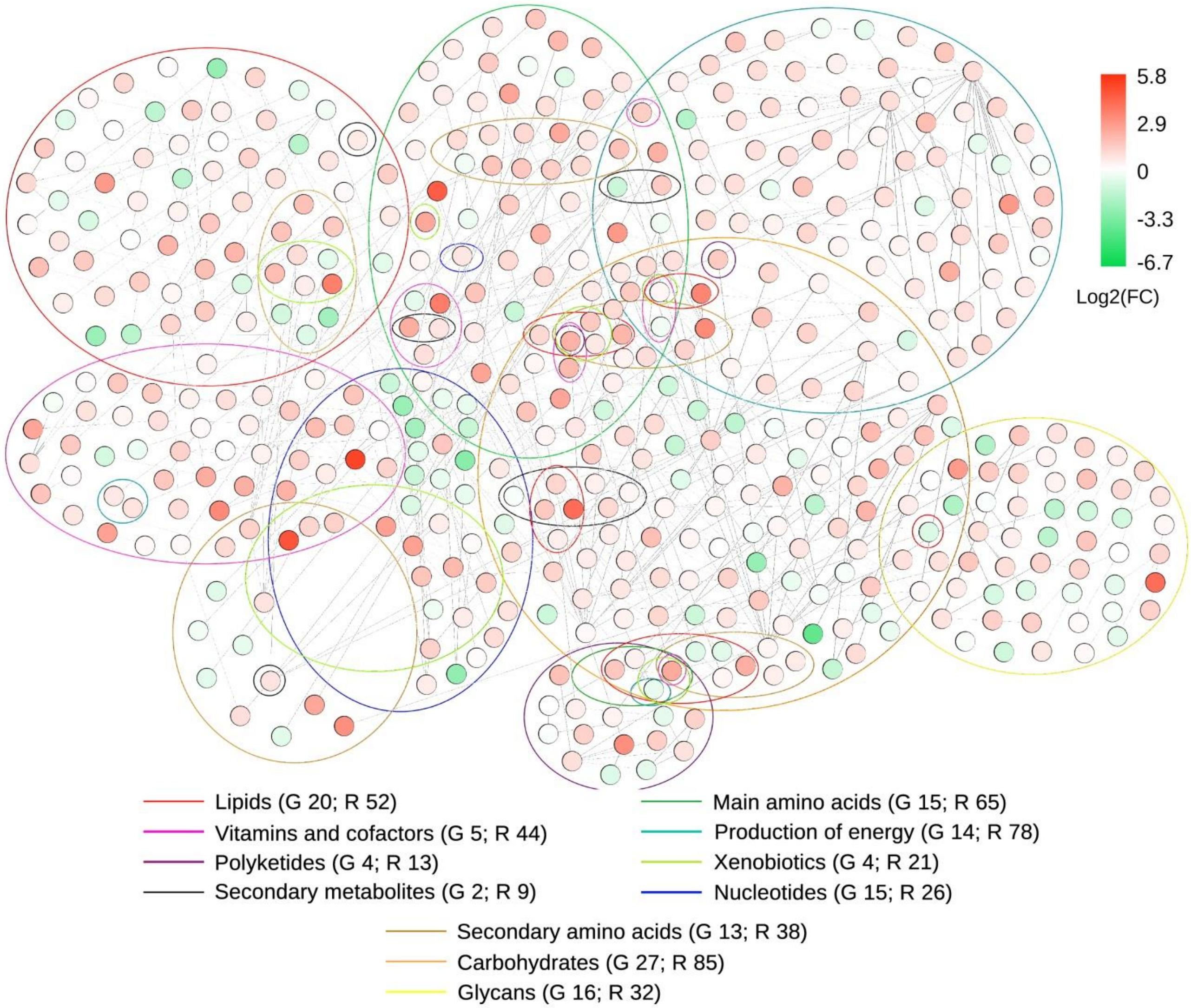
5.8
2.9
0
-3.3
-6.7
Log2(FC)
Lipids (G 20; R 52)
Vitamins and cofactors (G 5; R 44)
Polyketides (G 4; R 13)
Secondary metabolites (G 2; R 9)
Main amino acids (G 15; R 65)
Production of energy (G 14; R 78)
Xenobiotics (G 4; R 21)
Nucleotides (G 15; R 26)
Secondary amino acids (G 13; R 38)
Carbohydrates (G 27; R 85)
Glycans (G 16; R 32)

BIII

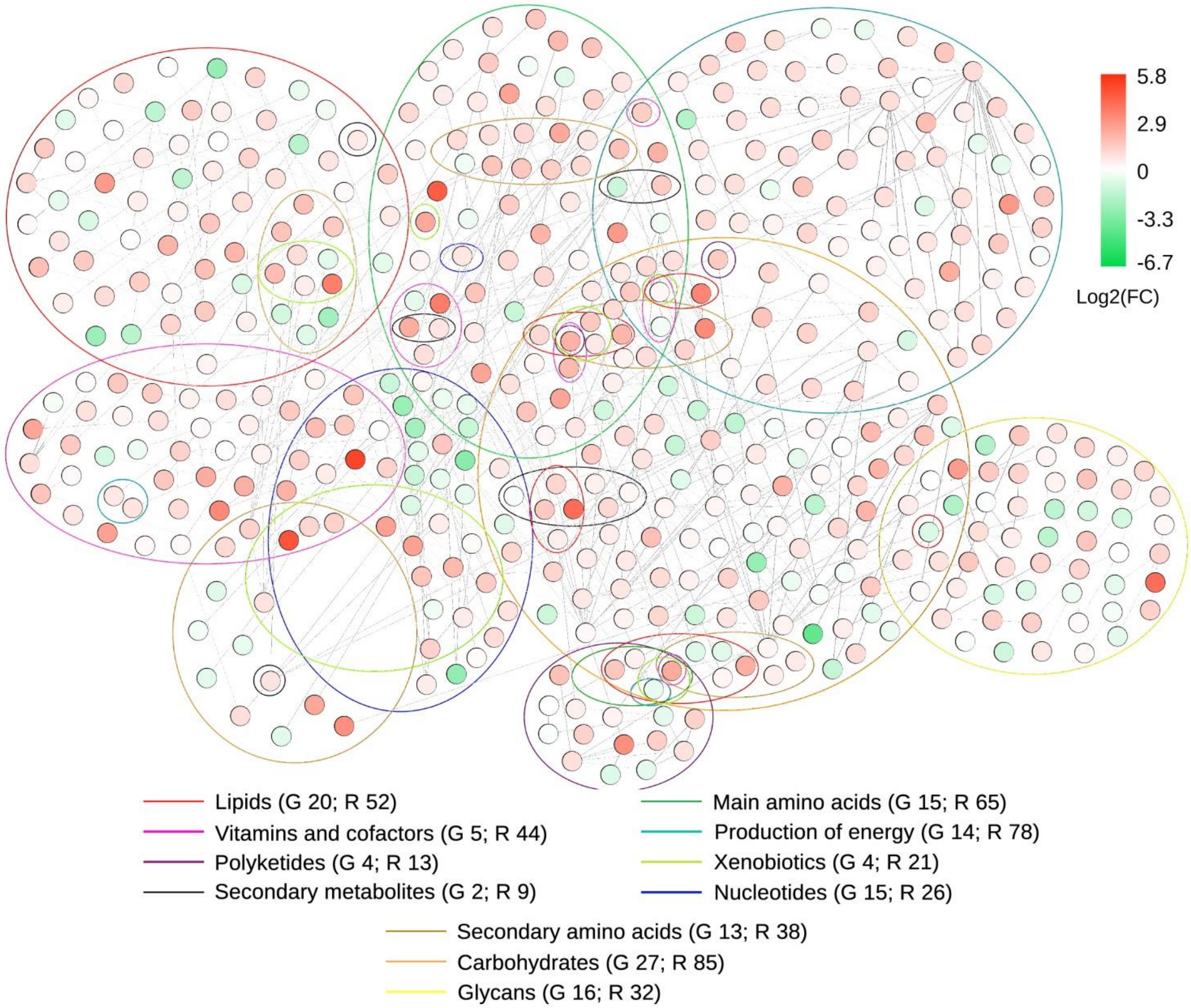

Supplementary Figure 16. Differential expression of the biosynthesis and metabolism pathways of the biological network components present in analyzed tissues of *R. neglectus.* Salivary gland: A-I. Fed / Fasting. A-II. Fed and infected 2 days/Fasting. A-III. Fed and infected 9 days/Fasting. Bowel: B-I. Fed / Fasting. B-II. Fed and infected 2 days/Fasting B-III. Fed and infected 9 days/Fasting. *G: green; A: red. FC: fold change. Identified homologous component clusters and with degree > 0.

AI

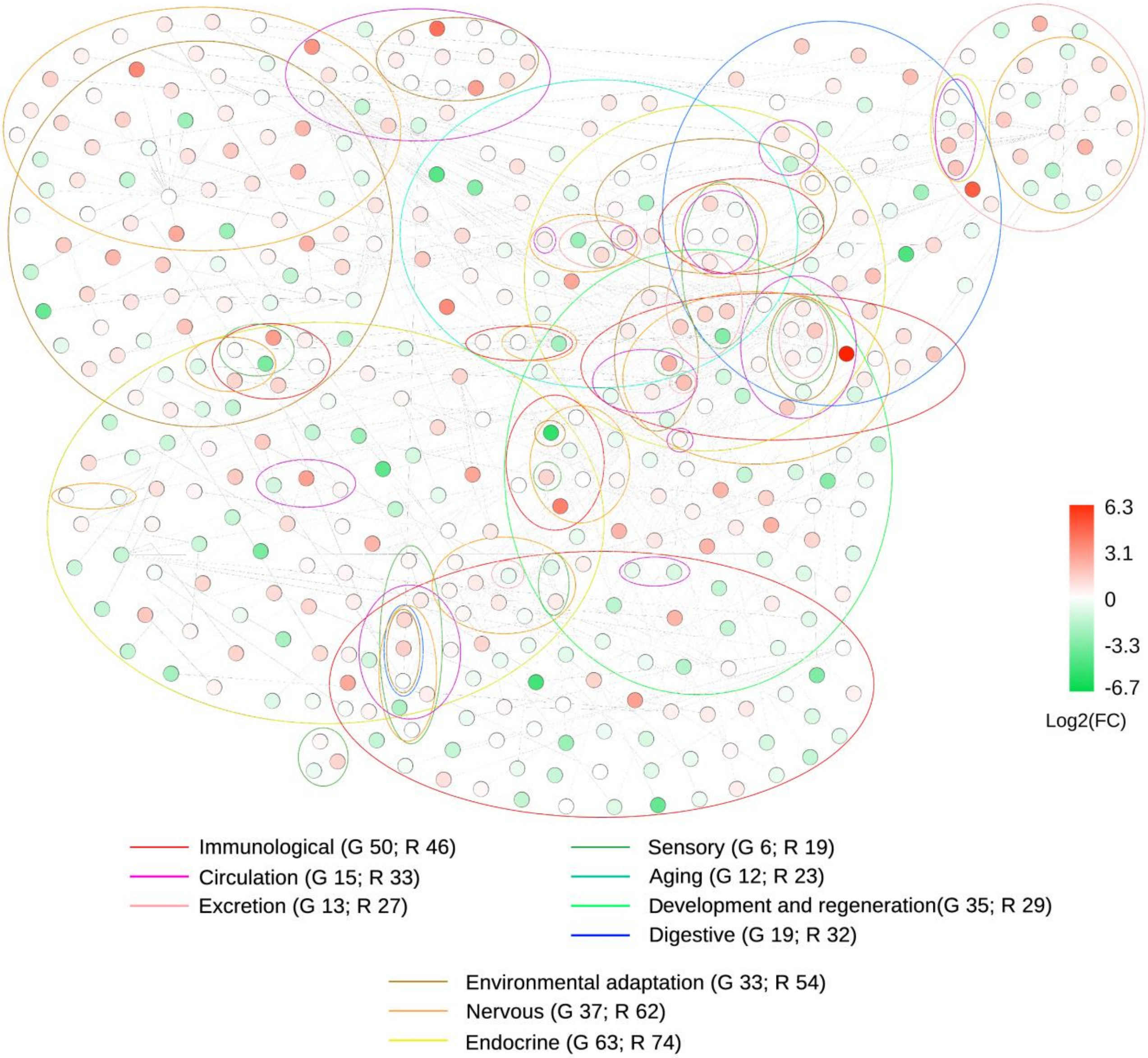
6.3
3.1
0
-3.3
-6.7
Log2(FC)
Immunological (G 50; R 46)
Circulation (G 15; R 33)
Excretion (G 13; R 27)
Sensory (G 6; R 19)
Aging (G 12; R 23)
Development and regeneration(G 35; R 29)
Digestive (G 19; R 32)
Environmental adaptation (G 33; R 54)
Nervous (G 37; R 62)
Endocrine (G 63; R 74)

AII

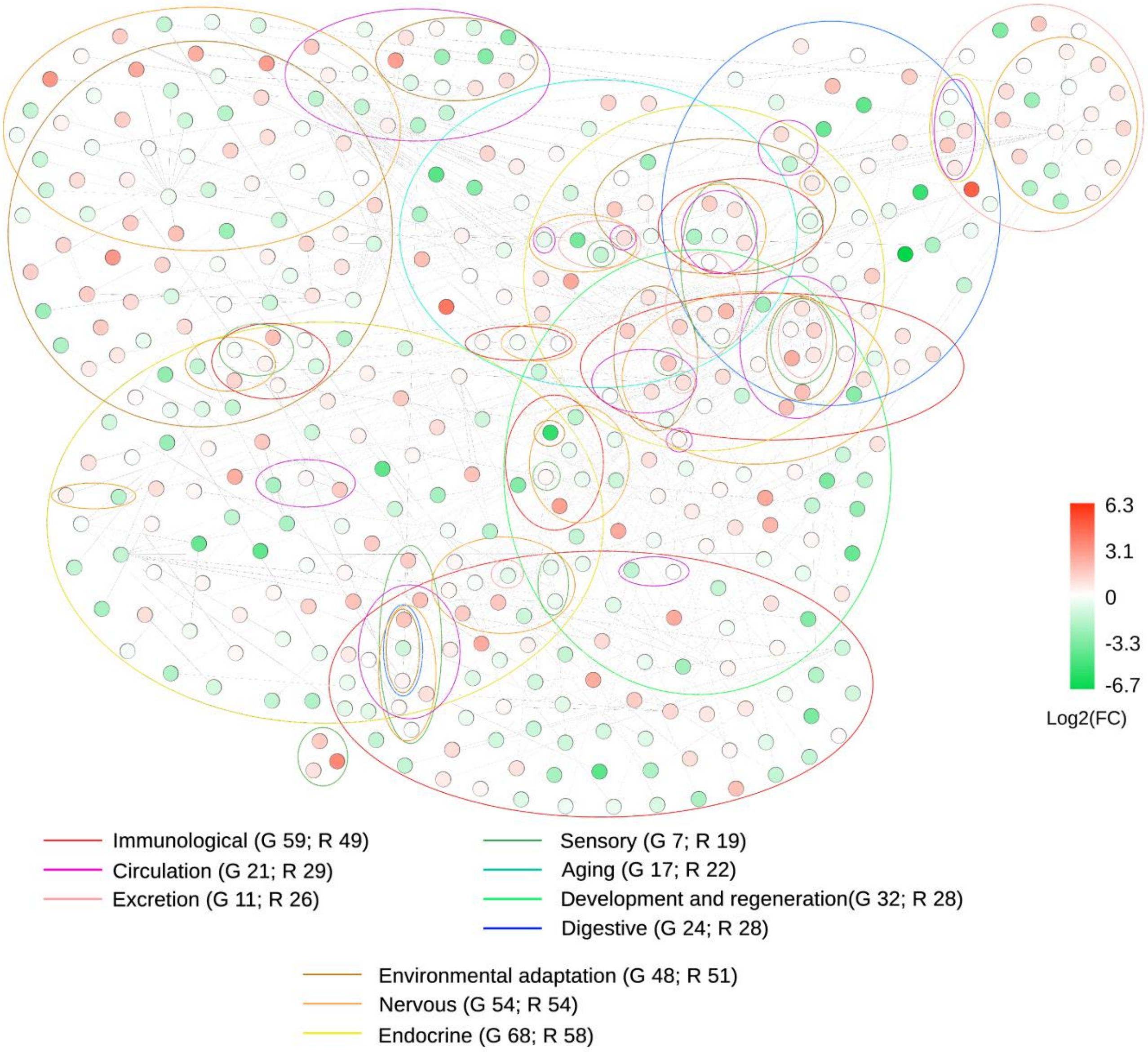

6.3
3.1
0
-3.3
-6.7
Log2(FC)
Immunological (G 59; R 49)
Circulation (G 21; R 29)
Excretion (G 11; R 26)
Sensory (G 7; R 19)
Aging (G 17; R 22)
Development and regeneration(G 32; R 28)
Digestive (G 24; R 28)
Environmental adaptation (G 48; R 51)
Nervous (G 54; R 54)
Endocrine (G 68; R 58)

AIII

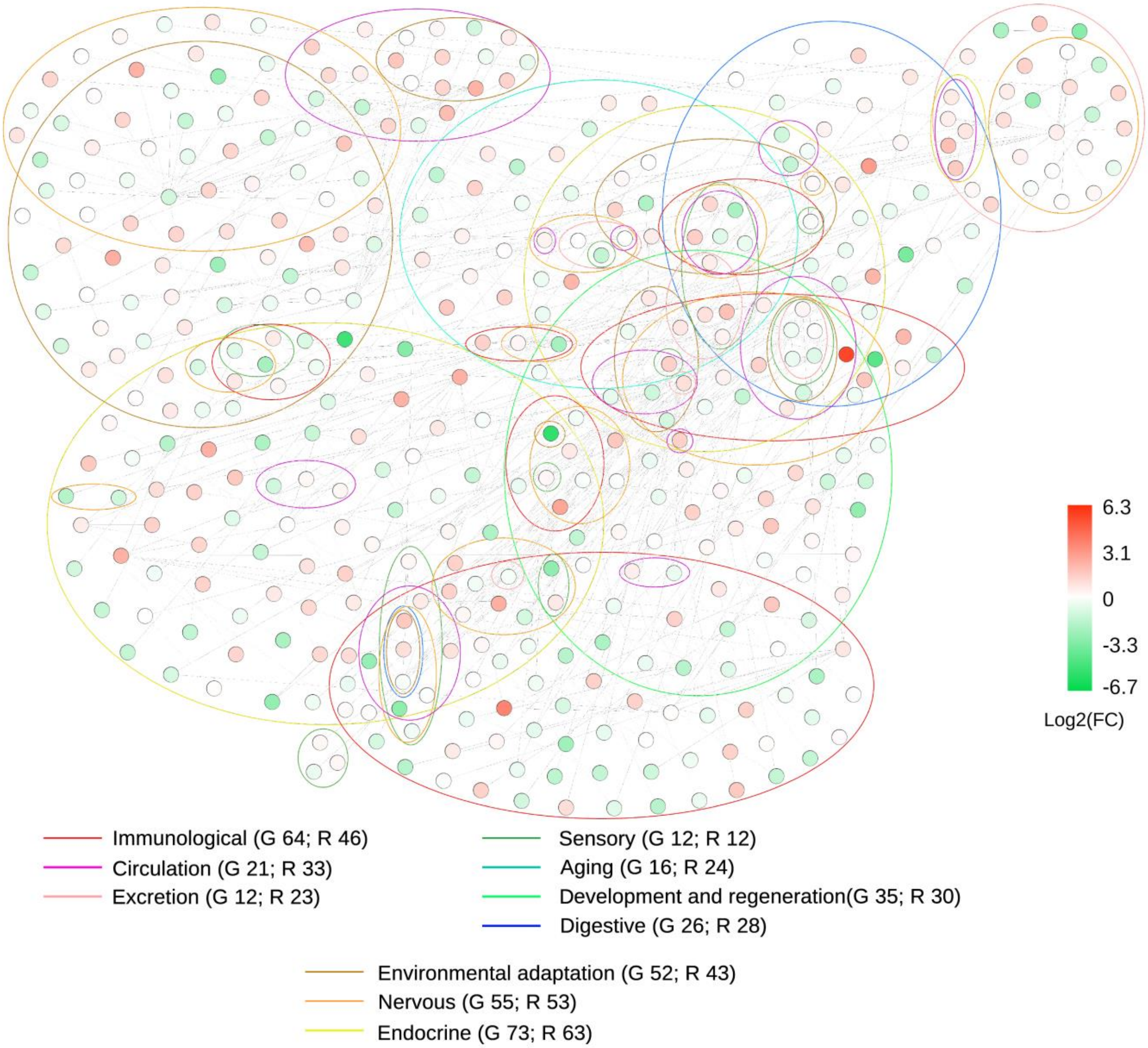
6.3
3.1
0
-3.3
-6.7
Log2(FC)
Immunological (G 64; R 46)
Circulation (G 21; R 33)
Excretion (G 12; R 23)
Sensory (G 12; R 12)
Aging (G 16; R 24)
Development and regeneration(G 35; R 30)
Digestive (G 26; R 28)
Environmental adaptation (G 52; R 43)
Nervous (G 55; R 53)
Endocrine (G 73; R 63)

BI

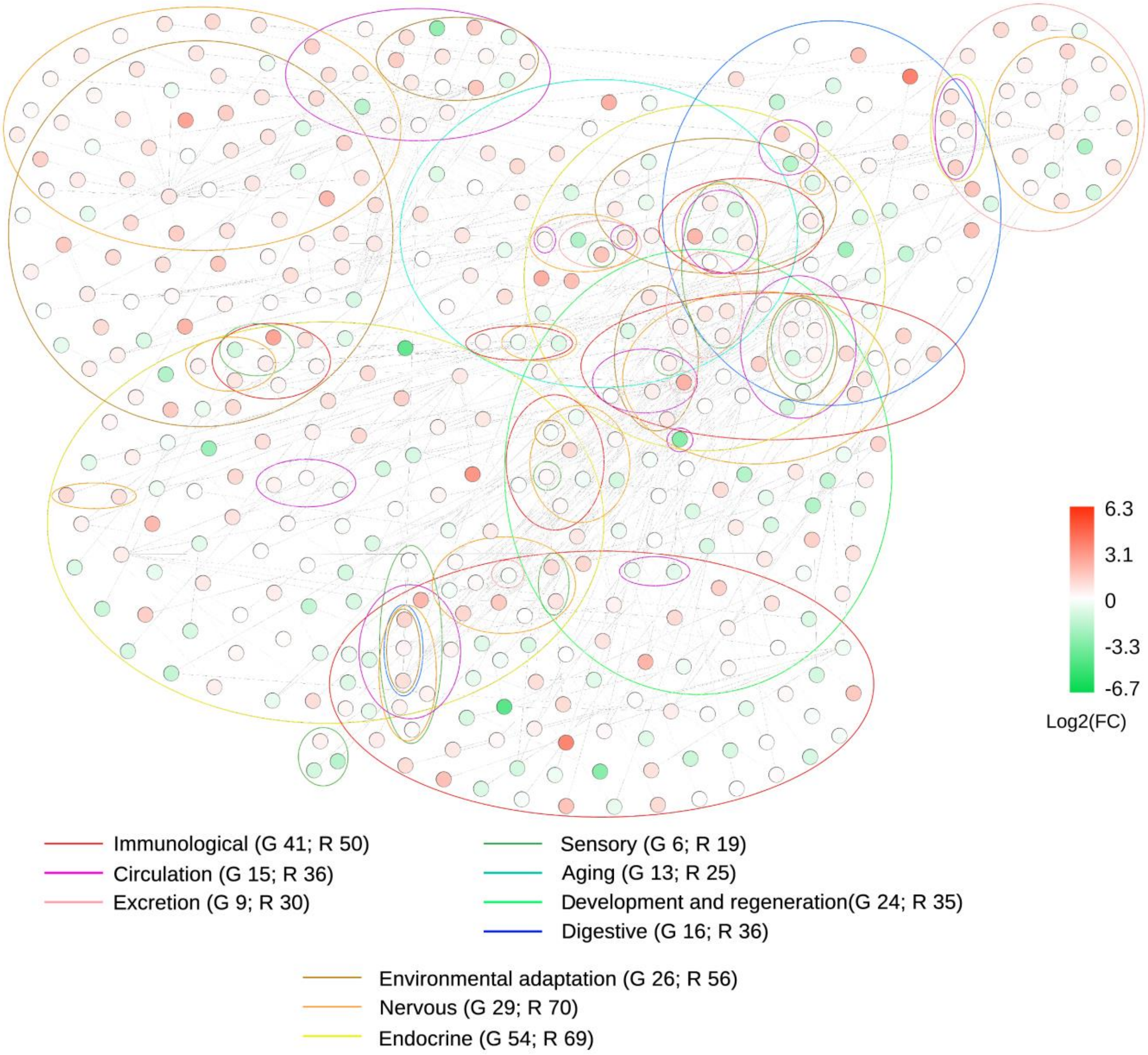
6.3
3.1
0
-3.3
-6.7
Log2(FC)
Immunological (G 41; R 50)
Circulation (G 15; R 36)
Excretion (G 9; R 30)
Sensory (G 6; R 19)
Aging (G 13; R 25)
Development and regeneration(G 24; R 35)
Digestive (G 16; R 36)
Environmental adaptation (G 26; R 56)
Nervous (G 29; R 70)
Endocrine (G 54; R 69)

BII

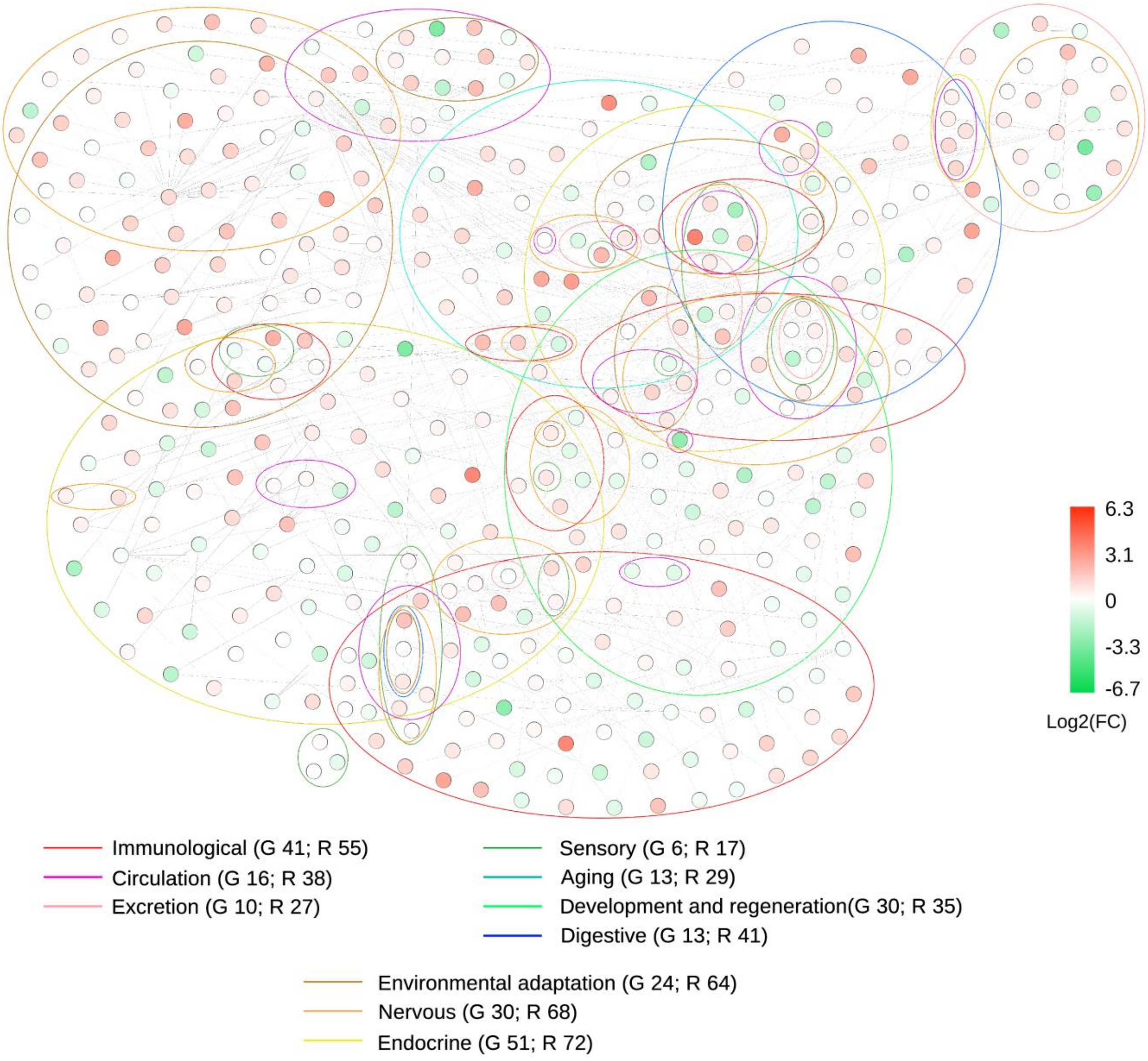
6.3
3.1
0
-3.3
-6.7
Log2(FC)
Immunological (G 41; R 55)
Circulation (G 16; R 38)
Excretion (G 10; R 27)
Sensory (G 6; R 17)
Aging (G 13; R 29)
Development and regeneration(G 30; R 35)
Digestive (G 13; R 41)
Environmental adaptation (G 24; R 64)
Nervous (G 30; R 68)
Endocrine (G 51; R 72)

BIII

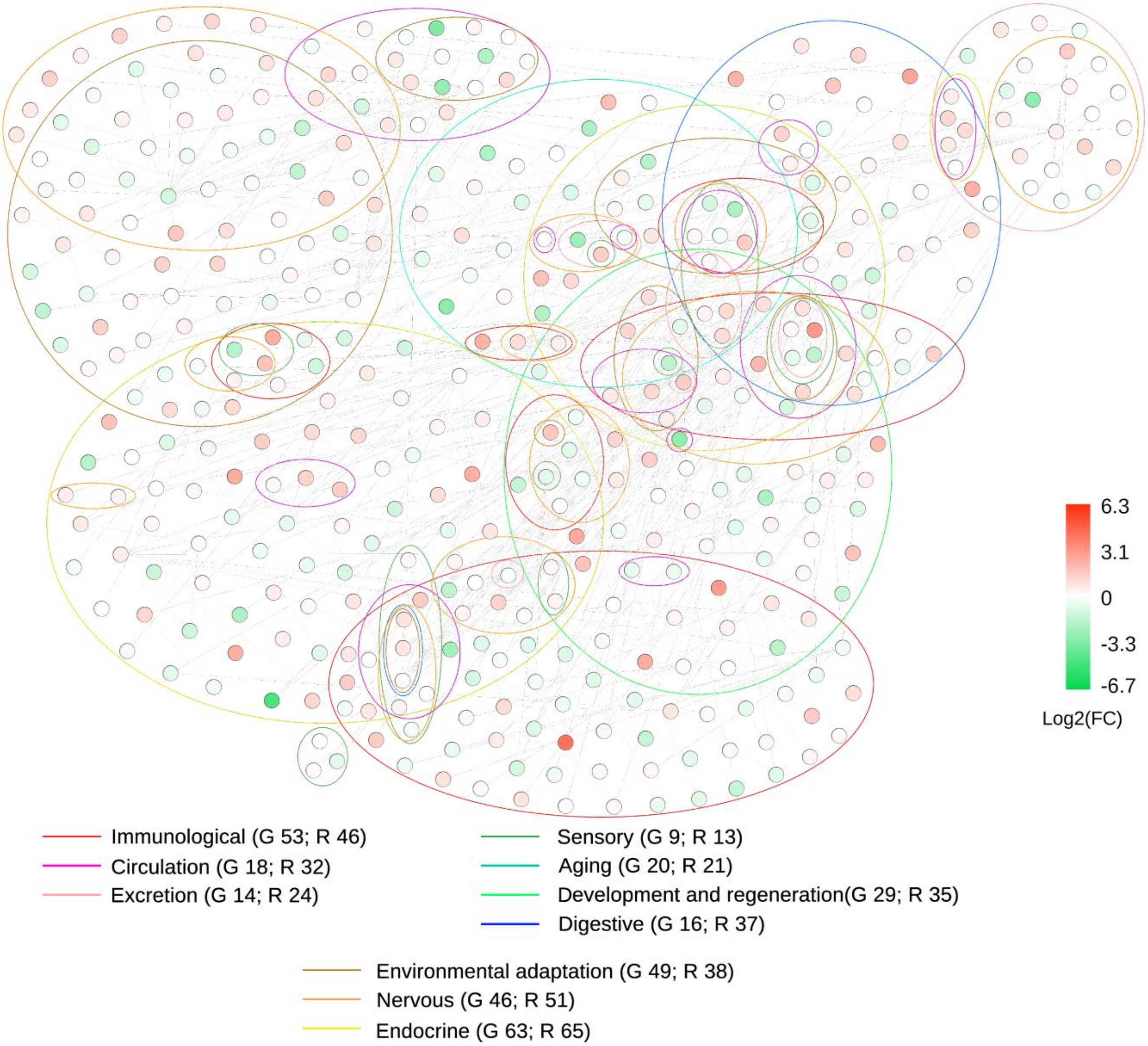

Supplementary Figure 17. Differential expression of the systemic pathways of components of the biological network present in the analyzed tissues of *R. neglectus*. Salivary gland: A-I. Fed / Fasting. A-II. Fed and infected 2 days/Fasting. A-III. Fed and infected 9 days/Fasting. Bowel: B-I. Fed / Fasting. B-II. Fed and infected 2 days/Fasting B-III. Fed and infected 9 days/Fasting. *G: green; A: red. FC: fold change. Identified homologous component clusters and with degree > 0.
